# Ru_n_Pd_1_ fully-exposed clusters for solvent-free hydrogenation of toluene toward hydrogen storage

**DOI:** 10.1038/s41467-026-75738-7

**Published:** 2026-08-24

**Authors:** Yue Wang, Hongqiu Chen, Jiawei Chen, Zhehan Ying, Lini Yang, Mi Peng, Jingwang Zhang, Yang Si, Jiangyong Diao, Geng Sun, Ding Ma, Hongyang Liu

**Affiliations:** 1 Department of Chemistry, Liaoning University, 66 Chongshan Road, Shenyang, Liaoning 110036 China; 2 Shenyang National Laboratory for Materials Science, Institute of Metal Research, Chinese Academy of Sciences, Shenyang, 110016 P. R. China; 3 Chongqing Key Laboratory of Chemical Theory and Mechanism, School of Chemistry and Chemical Engineering, Chongqing University, Chongqing, 401331 P. R. China; 4 School of Materials Science and Engineering, University of Science and Technology of China, Shenyang, 110016 P.R. China; 5 Materials Characterization and Preparation Facility (GZ), The Hong Kong University of Science and Technology (Guangzhou), Guangzhou, China; 6 Beijing National Laboratory for Molecular Sciences, College of Chemistry and Molecular Engineering, Peking University, Beijing, 100871 P. R. China

**Keywords:** Catalyst synthesis, Heterogeneous catalysis, Catalytic mechanisms

## Abstract

Liquid organic hydrogen carriers are promising for large-scale and long-distance hydrogen storage and transportation. Considering that renewable hydrogen sources are often decentralized and intermittent, designing a catalyst that is simultaneously cost-effective, room-temperature compatible, and environmentally-friendly remains a significant challenge. This contribution introduce a single Pd_1_ assisted fully exposed Ru clusters supported on defective graphene/nanodiamonds hybrid support (Ru_n_Pd_1_/ND@G) catalyzing efficient toluene hydrogenation under solvent-free conditions, in which the conversion of toluene could achieve 100% even at room temperature. Remarkably, Ru_n_Pd_1_ fully-exposed cluster delivers an exceptionally high turnover frequency of 35199.5 h^−1^ at the absence of solvent, which is 5.9 times higher than that of the monometallic Ru/ND@G catalyst. The catalyst exhibits maximized atomic efficiency, excellent recyclability and reaction scalability. Combing with theoretical calculations, it is revealed that Pd_1_ assisted fully-exposed Ru cluster catalysts promoted H_2_ activation and C-H formation as well as improved reactant (product) adsorption (desorption), which all contribute to the superior activity of Ru_n_Pd_1_ fully-exposed clusters. This work offers a practical strategy for efficient hydrogen energy utilization under solvent-free conditions.

## Abstract (web summary)

Liquid organic hydrogen carriers are promising for cost-effective, large-scale, and long-distance hydrogen storage and transportation. Here, the authors report a Pd1 assisted fully exposed Ru cluster supported on defective graphene/nanodiamonds hybrid support which catalyzes efficient toluene hydrogenation under solvent-free conditions.

## Introduction

Hydrogen, as a renewable and clean energy carrier, is widely regarded as one of the most promising alternatives to fossil fuels for future energy systems^1–3^. However, large-scale applications of hydrogen energy are limited by the challenges of safe storage and transportation. Decades of efforts have been devoted to overcoming these challenges, leading to the development of various hydrogen storage technologies, including metal hydrides, complex hydrides, chemical hydrides, and liquid organic hydrogen carriers (LOHCs)^4^. Among them, LOHCs have attracted much attention due to their low cost, excellent reversibility, high hydrogen storage capacity, as well as compatibility with existing gasoline infrastructure. Furthermore, the practicality of LOHCs for large-scale, long-distance hydrogen transportation and storage has been successfully validated in several countries^5^. The toluene/methylcyclohexane (TOL/MCH) system is considered a highly promising candidate among LOHCs for long-distance hydrogen storage and transport, owing to its high hydrogen storage capacity (6.2 wt%)^6,7^, low toxicity and liquid state under ambient conditions. However, the conversion of TOL to MCH typically requires catalysts consisting of expensive noble metals^8–10^, thus posing a key challenge for its large-scale application. Therefore, it is urgent to develop cost-effective and efficient catalysts to improve the economic feasibility of LOHC processes.

One strategy to improve the cost-effectiveness of noble-metal catalysts is to reduce their size. Many previously reported hydrogenation catalysts were typically metal nanoparticles larger than 2 nm in size, which limited the noble metal utilization and thereby increased their cost^11–15^. In recent years, fully exposed cluster catalysts (FECCs) have emerged as a type of catalyst have attracted growing attention as highly active species for various catalytic reactions^5,16^. The catalysts feature an ultrasmall size below 1 nm, which eliminates bulk atoms and reduce the average coordination number of metal atoms, as well as a small contact angle with the support that form a layered structure to enhance metal-support interaction and cluster stability^17,18^. Achieving an optimal cluster size offers a dual benefit: it retains the active-site ensemble required for multistep hydrogenation and enhances the utilization efficiency of precious noble metals. In addition, FECCs also provide advantages over single-atom catalysts with limited active sites, whose performance is typically suboptimal for multi-step hydrogenation. On the other hand, the introduction of the second metal element into nanoclusters offers an additional vector to modulate the activity of nanocluster catalysts^19,20^. Recent studies have demonstrated that bimetallic catalysts exhibit superior catalytic activity compared to their monometallic counterparts in multistep reactions, and such an improvement is primarily attributed to electronic **s**tructure modification^21–25^. Among various heterogeneous catalysis. For example, Tang^26^ et al. reported Ru-Pd alloy nanoparticles (RuPd/CN) catalyst, which demonstrated high activity for the hydrogenation of benzoic acid under mild conditions owing to a synergistic effect between the metals. Guo^27,28^et al. demonstrated the RuPd_5_/NH_2_-SiO_2_ nanoparticle catalyst in the cleavage of the diphenyl ether C-O bond and reported the Pd-Ru/C catalyze benzofuran hydrogenation, the enhanced catalytic performance of bimetallic Pd-Ru/C catalysts was attributed to the cooperative effect between Ru and Pd.

In order to retain the integrity of LOHCs as a clean energy source to the maximum extent, it is essential to integrate the principles of green chemistry across the entire LOHC process^29^. Considering that renewable hydrogen sources, such as biomass and wind- or hydropower-driven water electrolysis, are often decentralized and intermittent, it is crucial to design a catalyst that operates at room temperature, thereby eliminating the need for additional heating equipment. Additionally, solvent-free operation is another important requirement, as separation equipment and solvent disposal can pose environmental contamination risks and high energy consumption^30–33^. Therefore, there is a strong incentive to construct highly active catalyst systems that do not require the use of a solvent for the efficient hydrogenation of TOL to MCH using H_2_.

In this work, we reported a facile and reliable strategy to synthesize Pd_1_ assisted fully-exposed Ru cluster catalysts (Ru_n_Pd_1_/ND@G) on defective graphene/nanodiamonds (ND@G) hybrid support. The Ru_n_Pd_1_/ND@G catalyst exhibits excellent catalytic activity in the solvent-free hydrogenation of TOL to MCH, showing a turnover frequency (TOF) of 35199.5 h^−1^ under solvent-free reaction conditions, which is 5.9 times higher than that of the monometallic Ru/ND@G catalyst. Combined experimental and density functional theory (DFT) calculations demonstrated that the unique Ru_n_Pd_1_/ND@G catalyst structure modified the electronic structure of Ru by introducing Pd, thereby enhancing the adsorption of TOL. Furthermore, the synergistic interaction between Pd_1_ and fully exposed Ru cluster sites lowered the activation energy barrier for H_2_ dissociation and C-H bond formation, and facilitated MCH desorption, resulting in highly active, solvent-free TOL hydrogenation. This study provides a novel avenue for the design of fully exposed bimetallic cluster catalysts, demonstrating the potential for efficient catalytic hydrogenation in solvent-free industrial applications.

## Results

### Characterization

The graphene-coated nanodiamond (ND@G) was composed of a sp^3^-hybridized diamond core and highly defective sp^2^ graphene shells (Fig. S1). The unique surface of the carbon defect could anchor metal atoms via the M-C bond^34–36^. The Ru_n_Pd_1_/ND@G catalyst was prepared via a facile deposition precipitation method with loadings of Ru and Pd species determined to be 0.29 wt% and 0.048 wt%, respectively. As control catalysts, Ru/ND@G and Pd/ND@G catalysts were also synthesized with loadings of 0.30 wt% and 0.050 wt% for Ru and Pd, respectively, as determined by inductively coupled plasma optical emission spectrometry (ICP-OES) (Table S1). Meanwhile, the X-ray diffraction (XRD) pattern of the catalyst (Fig. S2) exhibited only the typical diffraction peaks of graphene and nanodiamond^37,38^, no diffractions associated with crystalline metal were observed, which indicated the Ru and Pd species were all highly dispersed in Ru_n_Pd_1_/ND@G, Ru/ND@G and Pd/ND@G catalysts. The N_2_ adsorption-desorption analysis showed that the structure of the ND@G support showed no significant difference after the deposition of Ru and Pd species, with the exception of the deposition active species (Table S1 and Fig. S3). Moreover, the structure of the catalysts was further characterized by Raman spectroscopy (Fig. S4), the *I*_D_/*I*_G_ value proved that there are still abundant defects after modification with active sites. To further characterize the morphology and structure of the catalysts, Ru_n_Pd_1_/ND@G catalyst was observed by the aberration-corrected high-angle annular darkfield scanning transmission electron microscopy (HAADF-STEM).

**Fig. 1:**
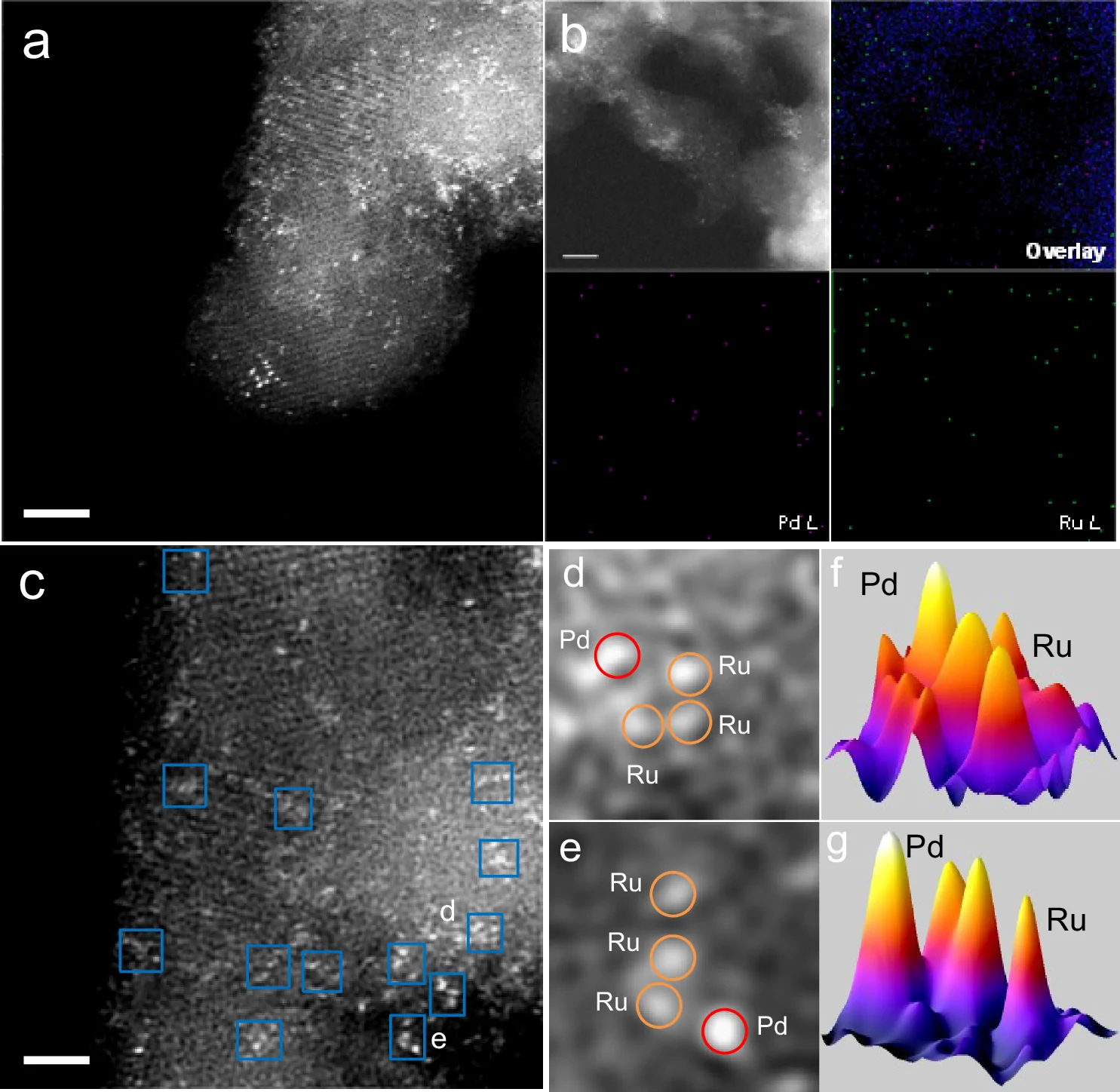
Microscopic characterizations of Ru_n_Pd_1_/ND@G. **a** HAADF-STEM image of Ru_n_Pd_1_/ND@G. **b** Energy-dispersive X-ray (EDX) mapping image of Ru_n_Pd_1_/ND@G. **c** The high resolution HAADF-STEM image of the Ru_n_Pd_1_/ND@G, where representative isolated Ru, Pd atoms and Pd_1_ assisted fully-exposed Ru cluster catalysts are highlighted by orange, red circles and dashed blue rectangles, respectively. **d**–**g** 3-D intensity surface plot shown for the dashed regions of the image in (**c**). Scale bars: **a** and **b**, 2 nm; **c**, 1 nm.

As shown in Fig. 1a, the Pd-Ru bimetallic clusters in Ru_n_Pd_1_/ND@G were well dispersed on the ND@G surface without any crystallized Ru or Pd nanoparticles^17^, which was consistent with the XRD results. Energy-dispersive X-ray spectroscopy (EDX) analysis in Fig. 1b confirmed that Ru and Pd elements within clusters were on the surface of ND@G. The magnified HAADF-STEM images (Fig. 1c-e) revealed fully exposed atomic clusters, highlighted by blue rectangles, owing to the Z-contrast between the metal atoms and the carbon support. The extensive HAADF-STEM images at different regions and magnifications were shown in Fig. S5. These clusters consisted of several compact spots packed together, corresponding to several Ru atoms (orange circles) and an adjacent Pd_1_ (red circles). The three-dimensional (3D) intensity surface plots (Fig. 1f, g) corresponding to the marked sites in Fig. 1c, distinctly distinguished between Ru and Pd based on their differing signal intensities, confirming the presence of Pd_1_ and fully-exposed Ru cluster. Additional HAADF-STEM images of Ru/ND@G and Pd/ND@G catalysts synthesized under identical conditions (Figs. S6-7), further demonstrated that both monometallic Ru and Pd species were highly dispersed on the ND@G surface.

**Fig. 2:**
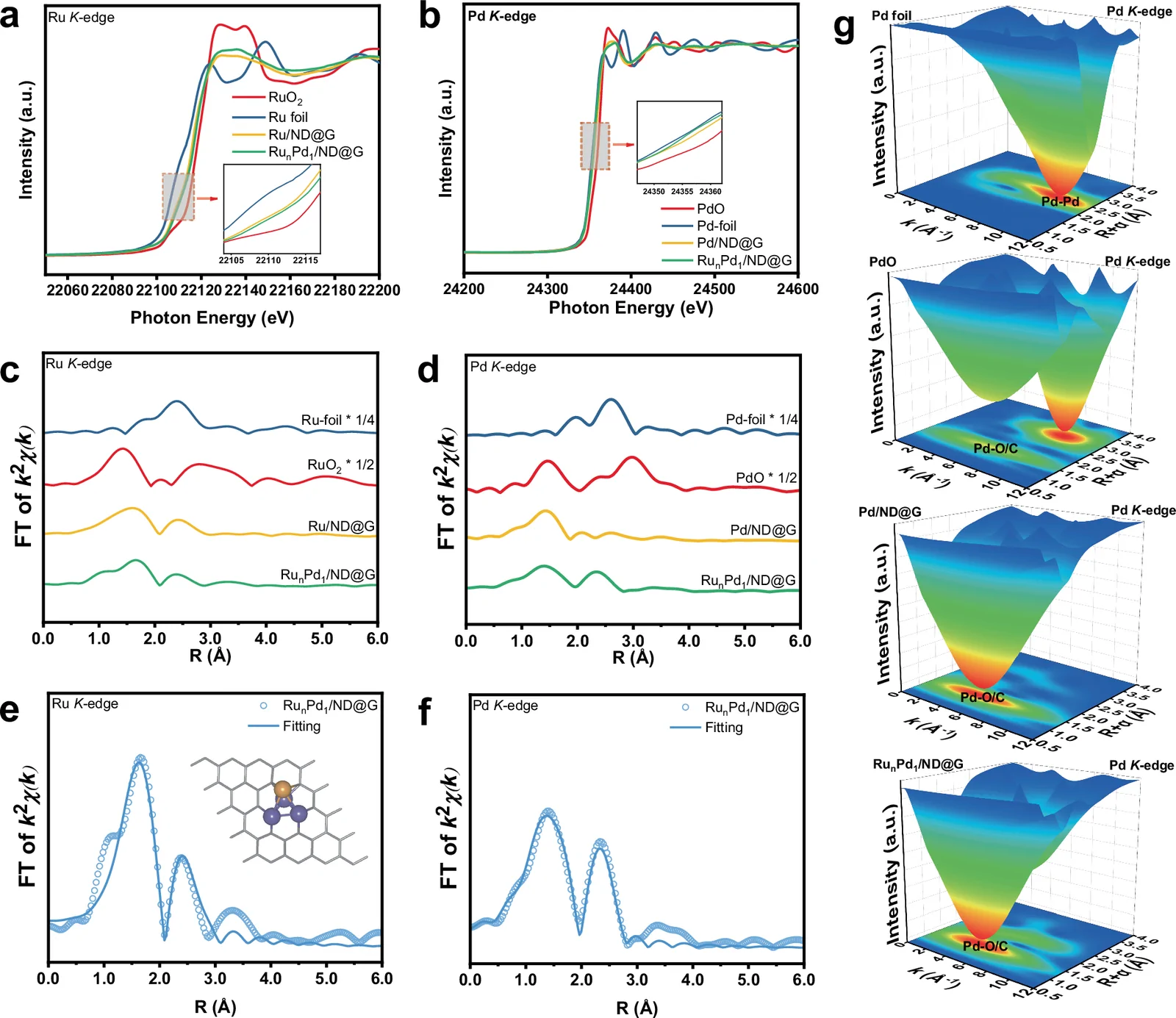
XAFS measurements of catalysts. **a** XANES and **c** EXAFS spectra of Ru/ND@G and Ru_n_Pd_1_/ND@G at the Ru *K*-edge. **b** XANES and **d** EXAFS spectra of Pd/ND@G and Ru_n_Pd_1_/ND@G at the Pd *K*-edge. **e, f** The corresponding Ru *K*-edge and Pd *K*-edge EXAFS fitting curves for Ru_n_Pd_1_/ND@G catalyst at R-space, respectively. **g** Wavelet transform (WT) analysis of Pd/ND@G and Ru_n_Pd_1_/ND@G at the Pd *K*-edge and the reference samples.

To investigate the electronic structure and coordination environment of the Pd_1_ and fully-exposed Ru clusters for Ru_n_Pd_1_/ND@G catalyst, the X-ray absorption fine structure (XAFS) spectra were collected at Ru *K*-edge and Pd *K*-edge. As depicted in X-ray absorption near-edge structure (XANES) spectra, the white line intensity of the Ru_n_Pd_1_/ND@G catalyst was positioned between those of Ru foil and RuO_2_ (Fig. 2a), indicating that the Ru species in Ru_n_Pd_1_/ND@G catalyst were in an oxidation valence state between 0 and +4. Moreover, the absorption edge of Ru_n_Pd_1_/ND@G catalyst shifted to a higher energy compared to that of Ru/ND@G catalyst, indicating that the Ru species in Ru_n_Pd_1_/ND@G catalyst were more electron-deficient and possessed higher positive valence states. As the differences in the Pd *K*-edge XANES are very small, the first-derivative XANES spectra were used to distinguish Pd valence states between the Ru_n_Pd_1_/ND@G and Pd/ND@G catalysts (Fig. S8). The Pd species in Pd/ND@G exhibited a state close to Pd^2+^ valence. In contrast, the Pd species in Ru_n_Pd_1_/ND@G displayed a lower valence state than that in Pd/ND@G. Additionally, Fourier-transform extended X-ray absorption fine structure (FT-EXAFS) spectra were employed to clarify the configurations of Ru atoms and Pd atoms in Ru_n_Pd_1_/ND@G catalyst (Fig. 2c, d). For Ru_n_Pd_1_/ND@G and Ru/ND@G catalyst, the distinct peaks near 1.6 Å and 2.5 Å at Ru *K*-edge were assigned Ru-O/C and Ru-Ru coordination, respectively. Besides, Ru_n_Pd_1_/ND@G and Pd/ND@G catalyst in the Pd *K*-edge EXAFS spectra, both observed the first Pd-O/C peak at about 1.5 Å. Notably, an obvious peak at about 2.3 Å was displayed in Ru_n_Pd_1_/ND@G catalyst, indicating the existence of Pd-Ru coordination. Then, the EXAFS fitting results of Ru_n_Pd_1_/ND@G catalyst (Fig. 2e, f, Fig. S9 and Table S2) showed the coordination number (CN) of Pd-Ru and Ru-Ru were 2.4 and 1.5.

X-ray photoelectron spectroscopy (XPS) was used to further confirm the electronic structure of Ru/ND@G and Ru_n_Pd_1_/ND@G catalysts. As shown in Fig. S10, the binding energy of Ru 3p in Ru_n_Pd_1_/ND@G catalyst shifted toward higher binding energy compared to that of Ru/ND@G catalyst, indicating a lower electron density and a higher valence states of Ru species. The shift in the binding energy of Ru 3p in Ru_n_Pd_1_/ND@G catalyst was in agreement with the XANES data, confirming potential electron transfer from Ru to Pd atoms. The wavelet transformation (WT) analysis of Pd EXAFS was carried out^39,40^. The maximum intensity of Pd/ND@G catalyst sample occurred at around 5.0 Å^−1^ in *k*-space and 1.5 Å in R-space, which could be attributed to the Pd-O/C path without the Pd-Pd path (*k* = 9.4 Å^−1^, r = 2.4 Å) (Fig. 2g), implying that the Pd single atom is coordinated only with carbon and/or oxygen atoms in monometallic Pd catalyst. In contrast, for the WT signal of Ru_n_Pd_1_/ND@G catalyst, the major peak at around 2.3 Å in R-space was exclusively observed, which was assigned to the Pd-Ru coordination.

### Catalytic performances

The hydrogenation of TOL was used to study the catalytic performance of Ru_n_Pd_1_/ND@G catalyst. First, the catalytic performance of Ru_n_Pd_1_/ND@G catalyst in the hydrogenation of TOL to MCH was initially performed under 1.5 MPa of H_2_ at 100 °C with addition of solvent (Fig. S11). Among various solvents, dodecane turned out to be the optimal solvent (Fig. S11a) and subsequent optimization of reaction temperature and hydrogen pressure (Fig. S11b, c) demonstrated that the reaction could be completed within 1 hour under the optimal conditions as shown in Fig. S11d (1.5 MPa H_2_, 100 °C). The Ru_n_Pd_1_/ND@G catalyst achieved complete conversion within 1 hour at 100 °C and 1.5 MPa H_2_, while the Ru/ND@G catalyst needed 6 h to reach full conversion under the same conditions (Fig. S12).

**Fig. 3:**
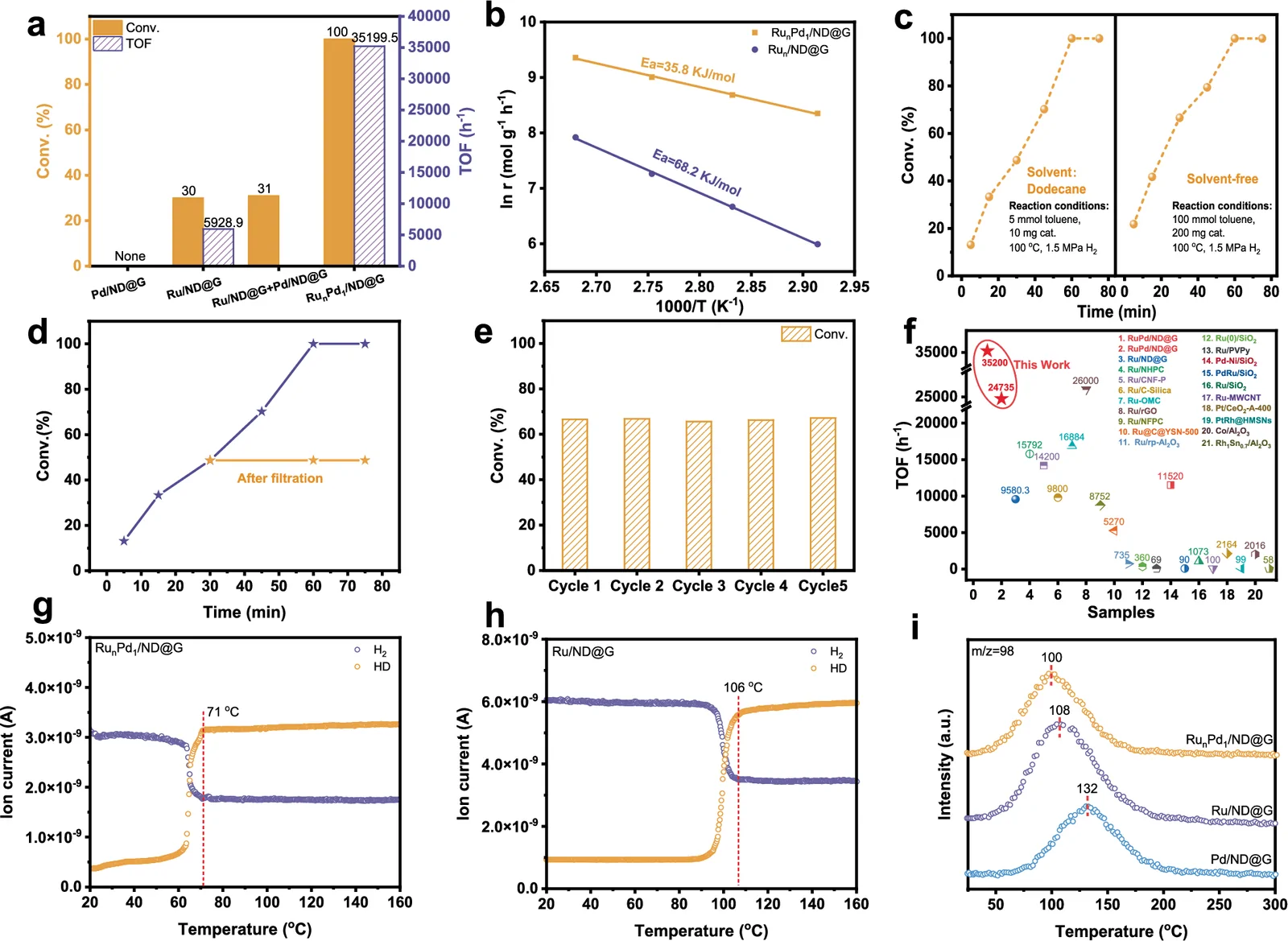
Catalytic performances and catalytic mechanism of catalysts. **a** TOF values and TOL conversion (1 h) of different catalysts under solvent-free conditions. (Reaction conditions: H_2_ pressure, 1.5 MPa; temperature, 100 °C; reaction time, 1 h.) **b** Apparent activation energies (*E*_a_) of different catalysts in TOL hydrogenation reaction. **c** Reaction scale tolerance of Ru_n_Pd_1_/ND@G for solvent-free hydrogenation of TOL reaction. **d** Hot filtration test of Ru_n_Pd_1_/ND@G in solvent-free hydrogenation of TOL. **e** Recyclability test of the Ru_n_Pd_1_/ND@G catalyst. **f** Comparison of reported TOF values over various catalysts for the hydrogenation of TOL. H_2_-D_2_ exchange experiments for **g** Ru_n_Pd_1_/ND@G and **h** Ru/ND@G. **i** MCH-TPD profiles of the Ru_n_Pd_1_/ND@G, Ru/ND@G and Pd/ND@G catalysts.

The catalytic performance of Ru_n_Pd_1_/ND@G catalyst for TOL hydrogenation was further evaluated under solvent-free conditions. We found that it exhibited remarkably high activity; the reaction yielded MCH with excellent conversion (100%) within 1 h under solvent-free conditions. We performed control experiments with monometallic catalysts and their physical mixture to exclude activity from isolated species. The Ru/ND@G catalyst exhibited a lower conversion of 30%, and the Pd/ND@G catalyst eventually showed negligible activity in TOL hydrogenation. The catalytic activity of the physical mixture was essentially consistent with that of monometallic Ru catalysts, confirming that the simple coexistence of both components did not generate the enhanced activity. The Ru_n_Pd_1_/ND@G catalyst achieved a TOF of 35199.5 h^−1^, which was 5.9 times higher than that of Ru/ND@G catalyst at the same solvent-free reaction condition (Fig. 3a). Additionally, Arrhenius plots showed that the apparent activation energy (*E*_*a*_) of the Ru_n_Pd_1_/ND@G catalyst (*E*_*a*_ = 35.8 kJ/mol) was significantly lower than that of Ru/ND@G (*E*_*a*_ = 68.2 kJ/mol), further indicating superior intrinsic activity and efficiency of Ru_n_Pd_1_/ND@G catalyst (Fig. 3b).

To verify the practicality of Ru_n_Pd_1_/ND@G catalyst for large-scale organic catalysis (substrate amount ≥10 mmol), the reaction was scaled up by 20 times relative to the laboratory-scale conditions. As shown in Fig. 3c and entries 3–5 in Table 1, the catalytic performance was nearly identical to that observed at the lab scale, highlighting the excellent reaction scale tolerance of Ru_n_Pd_1_/ND@G. Notably, catalysts used in industrial processes often suffer from loss of activity and stability due to metal leaching or sintering, which can be exacerbated by strong coordination of substrates or products with the metal surfaces^41–43^. In contrast, our catalyst maintained its performance under scaled-up conditions, underscoring its potential for practical applications. Notably, Ru_n_Pd_1_/ND@G catalyst exhibited excellent stability in solvent-free hydrogenation of TOL, as confirmed by hot filtration tests (as shown in Fig. 3d). Furthermore, after 5 cycles the Ru_n_Pd_1_/ND@G catalyst also exhibited stability without significant activity degradation (Fig. 3e). HAADF-STEM images revealed that the fully exposed bimetallic Pd-Ru clusters in the recycled catalyst remained highly dispersed on the ND@G surface without agglomeration (Fig. S13), indicating the excellent structural stability of Ru_n_Pd_1_/ND@G. Notably, the conversion of TOL maintained >99% even at room temperature and lower hydrogen pressure (1.0 MPa), demonstrating the high activity of Ru_n_Pd_1_/ND@G catalyst under milder conditions (entries 4-5 in Table 1). The solvent-free hydrogenation of TOL could reach 100% conversion even at room temperature, although longer reaction time was required, demonstrating the possibility of rendering the reaction at low temperature. In addition, the TOF values of Ru_n_Pd_1_/ND@G catalyst under solvent-free or addition of solvent reactions (Fig. 3f and Table S3) were higher than those of other catalysts reported in the literature^44–58^. The TOF of the other catalysts reported in the literature were obtained under different reaction conditions and solvents, as detailed in Table S3.

**Table 1:** Ru_n_Pd_1_/ND@G catalyzed the hydrogenation of toluene

Taba								
Entry	TOL	Cat.	T	P	Solvent	t	Conv.	TOF
	mmol	mg	^o^C	MPa		h	%	h^−1^
1	5	10	100	1.5	Dodecane	1	>99	24735.1
2	20	40	100	1.5	-	1	>99	35199.5
3	100	100	100	1.5	-	1	>99	35136.5
4	100	100	30	1.5	-	18	>99	2414.6
5	100	100	30	1.0	-	24	>99	2130.9

*TOL* toluene, *Cat.* catalyst, *Conv.* refers to the conversion of TOL in the products, *P* hydrogen pressure, *T* reaction temperature, *t* reaction time.

### Mechanism study

Considering that H_2_ activation/dissociation is one of the important elementary steps in hydrogenation^59,60^, the activation ability of the catalysts was conducted using hydrogen-deuterium (H_2_-D_2_) exchange experiments (Fig. 3g-h and Fig. S14). The HD signal of Ru_n_Pd_1_/ND@G was observed at 71 ^o^C, which was lower than that of Ru/ND@G (106 ^o^C). Although Pd/ND@G exhibited the lowest H_2_-D_2_ exchange temperature (63 ^o^C), the Ru_n_Pd_1_/ND@G showed no Pd-C/O or Pd-Pd bonds but contained Pd-Ru coordination. Furthermore, Pd/ND@G displayed negligible activity in toluene hydrogenation. Hence, the promotion of H_2_ dissociation is attributed to the incorporation of Pd and its synergistic interaction with Ru. The substrate and product adsorption was further investigated by temperature-programmed desorption (TPD). The results showed that the MCH desorption temperature on Ru_n_Pd_1_/ND@G catalyst (100 ^o^C) was lower than that on Ru/ND@G catalyst (108 ^o^C) and Pd/ND@G (132 ^o^C), which was shown in Fig. 3i. The lower desorption temperature indicates that MCH adsorption is facilitated, which contributed to the high reactivity of the Ru_n_Pd_1_/ND@G catalyst. Furthermore, TOL-TPD was performed to probe the adsorption ability of toluene on the three catalyst surfaces. The desorption temperature of toluene was 333°C on Ru_n_Pd_1_/ND@G and 303°C on Ru/ND@G (Fig. S15), indicating the enhanced adsorption of toluene on Ru-Pd sites. The weakened desorption of MCH and the enhanced adsorption of TOL synergistically contribute to the high reactivity of the Ru_n_Pd_1_/ND@G catalyst.

### DFT calculations

**Fig. 4:**
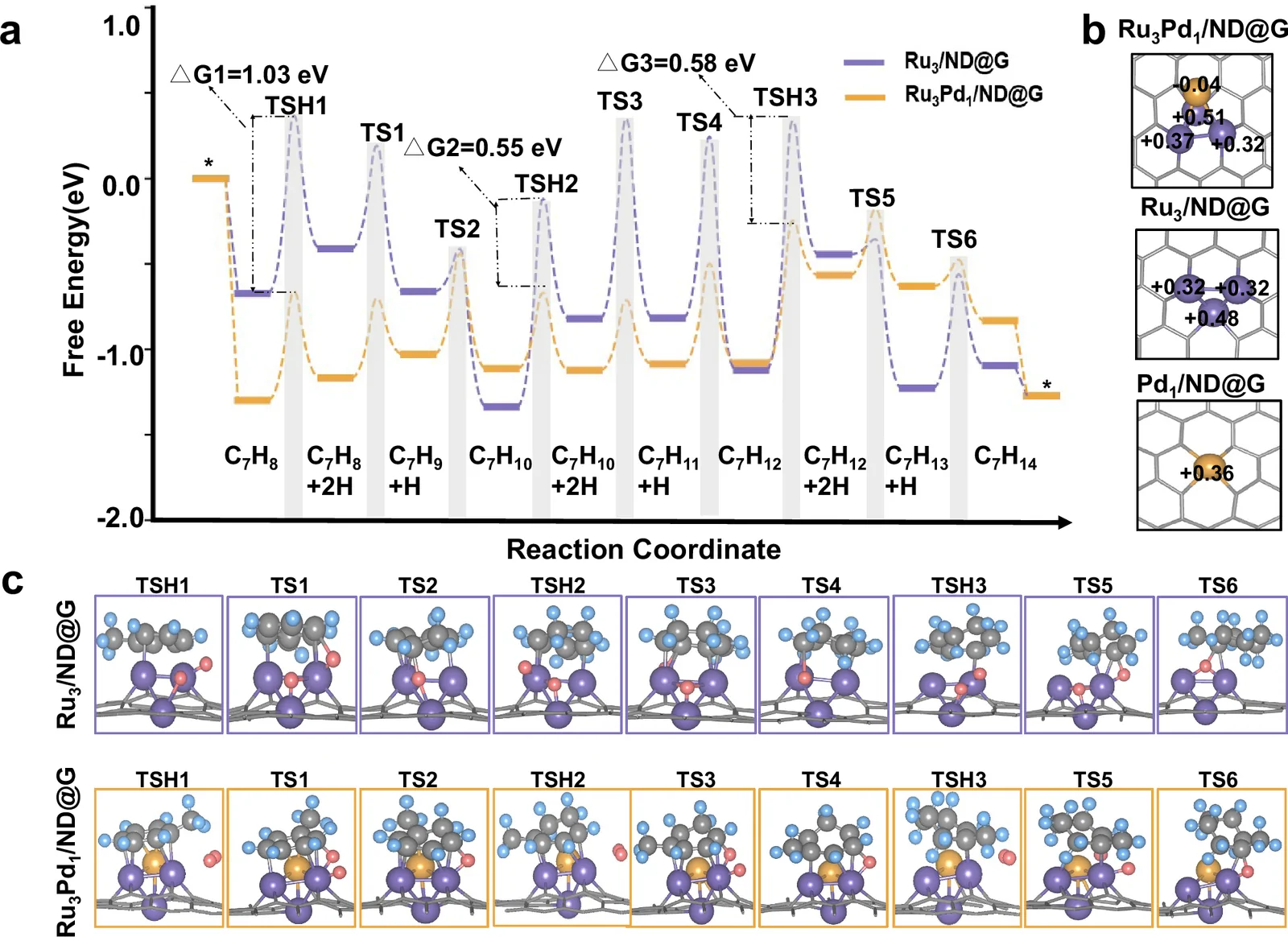
DFT calculations. **a** Free energy profiles for TOL hydrogenation to MCH on Ru_3_Pd_1_/ND@G and Ru_3_/ND@G (Reaction conditions: temperature 373.15 K, the partial pressure of H_2_ is 1.5 MPa, and the concentrations of the TOL and MCH in the solution are assumed as 0.11 in terms of its mole fraction). The gray shaded patches depict the transition states for clarity purposes. **b** Top views of DFT models for Ru_3_Pd_1_/ND@G, Ru_3_/ND@G and Pd_1_/ND@G, respectively, with Bader charges of metals shown. **c** Transition state structures for both H_2_ activation and hydrogenation on Ru_3_/ND@G (in purple box) and Ru_3_Pd_1_/ND@G (in orange box), in which the reacting hydrogen atoms are illustrated by red balls while other hydrogens in C-H bonds are shown with blue balls. Carbon atoms in the reactant are shown with gray balls, and carbon atoms on the graphene are only indicated by thin lines for simplicity. Ru atoms are shown with purple spheres, and Pd atoms are shown with yellow spheres.

To thoroughly investigate the remarkable enhancement in catalytic performance observed for the Ru_n_Pd_1_/ND@G catalyst under solvent-free conditions, we conducted density functional theory (DFT) calculations using the Vienna Ab initio Simulation Package (VASP 6.3)^61,62^. We constructed Ru_3_/ND@G, Pd_1_/ND@G and Ru_3_Pd_1_/ND@G models to represent Ru/ND@G (Fig. 4b and Fig. S16), Pd/ND@G (Fig. 4b and Fig. S17) and Ru_n_Pd_1_/ND@G (Fig. 4b and Fig. S18), respectively. The fully-exposed bimetallic cluster catalysts with different numbers of Ru atoms (Ru_2_ to Ru_4_) were investigated, as shown in Figs. S19−23, and according to the XAFS experiments, the Ru_3_Pd_1_ was adopted as the model. The varying electronic structures of Ru_3_/ND@G, Pd_1_/ND@G and Ru_3_Pd_1_/ND@G catalysts (Fig. 4b) also matched the white line positions from XANES (Fig. 2a). In particular, the Ru is positively charged in Ru_3_Pd_1_/ND@G with an average Bader charge of 0.4 $\left|e\right|$, being slightly larger than that in Ru_3_/ND@G 0.37 $\left|e\right|$, and this is in line with the slight right shifting of the white line positions of the Ru *K*-edge XANES (Fig. 2a). Conversely, the Bader charge of Pd in Ru_3_Pd_1_/ND@G was 0.04 $\left|e\right|$, significantly lower than that of in Pd_1_/ND@G 0.36 $\left|e\right|$. The results confirmed the enhanced Pd metallicity upon formation of Ru_3_Pd_1_/ND@G.

DFT calculations were exploited to investigate the reactivity of the proposed catalysts in TOL hydrogenation. The adsorption of TOL on Ru_3_Pd_1_/ND@G (adsorption energy was −2.48 eV) was stronger than that on Ru_3_/ND@G (−1.86 eV) (Fig. S24) and the desorption energy of MCH decreased from 1.01 eV to 0.74 eV by introducing Pd (Fig. S25). Those calculated adsorption energies agreed well with the MCH-TPD experiment (Fig. 3i and Fig. S15). The results indicated that the Ru_3_Pd_1_/ND@G catalyst delivers better reactant adsorption and product desorption kinetics, which potentially promoted the overall reaction kinetics. The detailed free energy profiles for reaction pathways towards TOL hydrogenation on Ru_3_/ND@G and Ru_3_Pd_1_/ND@G catalysts were shown in Fig. 4a. The transition state (TS) structures for H_2_ activation on Ru_3_/ND@G and Ru_3_Pd_1_/ND@G along with the TS structures for each hydrogenation reactions were shown in Fig. 4c. The first distinction between the two catalysts lied in the different capability for activating hydrogen molecules at the presence of co-adsorbates (TOL and subsequent hydrogenation intermediates) on the catalysts. We can see that the free energies of transition states for H_2_ activation on the Ru_3_/ND@G catalyst is 0.55−1.03 eV higher than on the Ru_3_Pd_1_/ND@G catalyst. In addition, the second key difference manifests in the hydrogenation of unsaturated C-C bonds in TOL, the maximum free energy heights of the transition state structures across the six-step hydrogenation reaction are 0.37 eV and −0.25 eV on Ru_3_/ND@G and Ru_3_Pd_1_/ND@G catalysts, respectively, showing that the hydrogenation reactions are much easier on Ru_3_Pd_1_/ND@G. Those results are consistent with the trend of the experimental determined reactivities (Fig. 3b).

Theoretical calculations revealed that the excellent performance of Ru_n_Pd_1_/ND@G catalyst originated from its unique electronic structures and the interactions between fully exposed Ru clusters and Pd single atoms. The introduction of Pd not only adjusted the electronic structure of the active sites, promoting H_2_ dissociation and C-H formation but also facilitated the desorption of MCH. The computational results well accounted for the exceptional multi-step hydrogenation activity of Ru_n_Pd_1_/ND@G compared to Ru/ND@G, consistent with experimental results.

## Discussion

In summary, Ru_n_Pd_1_ fully-exposed cluster catalyst (Ru_n_Pd_1_/ND@G) was successfully synthesized via a facile deposition-precipitation method. The structure analysis suggested Ru_n_Pd_1_ fully-exposed clusters were fabricated as active sites. The Ru_n_Pd_1_/ND@G catalyst exhibited exceptional catalytic performance in the solvent-free hydrogenation of toluene, superior to reported catalysts, while maintaining good stability. The solvent-free hydrogenation of TOL also could reach 100% conversion even at room temperature. Meanwhile, combined experiments and DFT calculations revealed that the introduction of Pd optimizes toluene adsorption, facilitates H_2_ activation, and enhances MCH desorption, resulting in the robust performance in the solvent-free hydrogenation process. This work not only paves a synthetic strategy for designing highly efficient bimetallic catalysts but also provides insights into the solvent-free hydrogenation reactions under mild conditions for industrial applications.

## Methods

### Materials. Nanodiamond (ND)

powders were procured from Beijing Grish Hitech Co., Ltd. Ruthenium (Ⅲ) chloride (RuCl_3_) was purchased from Aladin Chemical Reagent Inc. Palladium nitrate (Pd(NO_3_)_2_) was purchased from Aladin Chemical Reagent Inc.

### Synthesis of ND@G

The ND powder was annealed at a temperature 1100 ^o^C for 4 h in continuous Ar flow with a rate of 100 mL min^−1^. The powder was purified with concentrated HCl, repeatedly washed with deionized water, and finally vacuum-dried at 80 °C for 48 h to obtain ND@G.

### Preparation of Ru_n_Pd_1_/ND@G, Ru/ND@G and Pd/ND@G

The Ru_n_Pd_1_/ND@G samples were synthesized by a deposition precipitation method. Firstly, 200 mg ND@G was dispersed in 30 mL deionized water to achieve a homogeneous suspension under 30 min sonication. Then, the ND@G suspension was heated to 100 ^o^C in oil bath followed by the addition of 0.25 M Na_2_CO_3_ solution, which was adjusted to about 10. A solution of RuCl_3_ and Pd(NO_3_)_2_ was added dropwise into the suspension under continuous stirring for 1 h. Subsequently, the mixture was cooled to room temperature, collected by filtration, and then washed several times with deionized water to remove Na^+^ and CO_3_^2-^. Afterward, the powders were dried in a vacuum oven at 60 °C for 12 h. Finally, the powders were reduced in pure H_2_ (30 mL min^−1^) at 200 ^°^C for 1 h. The Ru/ND@G was prepared by the same method, but without the addition of Pd(NO_3_)_2_. For comparison, Pd/ND@G was also prepared using the same method without the addition of RuCl_3_. After drying under vacuum for 12 h, the samples were reduced in pure H_2_ (30 mL min^−1^) at 200  ^°^C for 1 h.

### Characterizations

High-resolution transmission electron microscopy (HRTEM) measurement was taken on FEI Tecnai G2 F20 working at 200 kV. HAADF-STEM images were recorded by a JEOL JEM ARM 200CF aberration-corrected cold field-emission scanning transmission electron microscope operating at 200 kV. X-ray absorption spectroscopy (XAS) was carried out at the BL14W1 station of Shanghai Synchrotron Radiation Facility (SSRF, 3.5 GeV, 250 mA in maximum, Si (111) double crystals). Wavelet transform (WT) of Ru *K*-edge EXAFS oscillations were implemented based on Morlet wavelets using HAMA Fortran. N_2_ physisorption was measured by the Micrometrics ASAP-2020 instrument. The specific surface area of samples was measured by the Brunauer-Emmett-Teller (BET) method and the pore size distribution was obtained from the Barrett-Joyner-Halenda (BJH) method from the data of the desorption branch. X-ray diffraction (XRD) patterns of supported Ru catalysts were collected by an X-ray diffractometer (D/MAX-2400) using a Cu Kα source at a scan rate of 2^°^/min. Ultraviolet Raman spectroscopy was performed on a powder sample using the HORIBA LabRam HR Raman spectrometer, with a 325nm laser source at 0.2 mW. The laser of λ = 325 nm used for Raman spectroscopy was calibrated with polytetrafluoroethylene. The X-ray photoelectron spectroscopy (XPS) was carried out at ESCALAB 250 instrument with Al Kα X-rays (1489.6 eV, 150 W, 50.0 eV pass energy).

H_2_-D_2_ exchange experiments were conducted in a quartz-bed flow reactor equipped with an online mass spectrometer (MS, Pfeiffer OMNIstarTM) to evaluate gas mixtures. Typically, 100 mg sample was reduced with pure H_2_ at 200 ^o^C for 1 h (flow rate = 20 mL min^−1^) and then cooled to room temperature in a He flow. Subsequently, the samples were further cooled in a He flow with an ice-water bath to 0 ^o^C. Then the mixture gas of 1 vol.% H_2_ and 0.5 vol.% D_2_ in a He flow (flow rate = 20 mL min^−1^) was introduced into the reactor. The signals of m/z values 2, 3 and 4 were recorded. After the signal of MS was stabilized, the sample was heated from 0 ^o^C with a heating rate of 2 ^o^C min^−1^, and the mass spectrum was continuously collected.

Temperature-programmed desorption (TPD) of MCH was carried out with an online mass spectrometer (MS, Pfeiffer OMNIstarTM) to evaluate gas mixtures. Initially, 100 mg of catalyst was reduced with pure H_2_ at 200 ^o^C for 1 h (flow rate = 20 mL min^−1^) and then cooled to room temperature in a He flow. Then TOL/MCH was introduced into the tube by passing helium (50 mL min^−1^) from the bubble reactor (maintaining the temperature of the bubble reactor at room temperature) and treated for 5 hours. Then, switching to pure He gas to obtain the stable signal of MS, the catalyst was heated with He from room temperature to 600 ^o^C with a heating rate of 2 ^o^C min^−1^, and the mass spectrum was continuously collected.

### Catalytic performance tests

An autoclave equipped with a magnetic stirrer was utilized for the hydrogenation reaction of toluene. For the reaction with solvents, 10mg of RunPd1/ND@G catalysts and 5mmol of toluene were mixed in 10 mL of solvent (dodecane). Subsequently, the autoclave was purged with Ar and H_2_ several times to remove the air in the autoclave, and then the reactor was heated to the reaction temperature with a magnetic stirrer (800 rpm). After reaching the reaction temperature, hydrogen was introduced and kept at the desired pressure. The corresponding organic compounds were extracted and analyzed by gas chromatography (GC) equipped with an HP-5 capillary column and a flame ionization detector. The qualitative analysis was performed by gas chromatography-mass spectrometry (GC 7890, Agilent). For the apparent activation energy measurement, all reactions were controlled to achieve low conversions (<20%) at each temperature.

For the reaction without solvents, 100 mmol of toluene and 200 mg Ru_n_Pd_1_/ND@G catalysts into the reactor. The autoclave was purged several times with Ar and H_2_.

The turnover frequency (TOF) values of the catalysts were determined at low conversion (<20%) of the substrate. The conversion and TOF are defined as following:

1 $${{\rm{Conversion}}}=\frac{{{\rm{mol}}}\; {{\rm{of}}}\; {{\rm{the}}}\; {{\rm{methylcyclohexane}}}}{{{\rm{mol}}}\; {{\rm{of}}}\; {{\rm{inlet}}}\; {{\rm{toluene}}}}\times 100\%$$

2 $${{\rm{TOF}}}=\frac{{{\rm{mol}}}\; {{\rm{of}}}\; {{\rm{converted}}}\; {{\rm{toluene}}}}{{{\rm{mol}}}\; {{\rm{of}}}\; {{\rm{total}}}\; {{\rm{noble}}}\; {{\rm{metal}}}\; {{\rm{atoms}}}\times {{\rm{reacton}}}\; {{\rm{time}}}}\times 100\%$$

### Stability test

The catalyst was reused for the hydrogenation reaction under identical conditions. After each run, the catalyst was recovered by filtration and washed sequentially with ethanol/deionized water to remove residual impurities. After that, the recycled catalyst was reduced in pure H_2_ (20 mL min^−1^) at 200 ^o^C for 1 h to remove the possibly adsorbed water and organic species and then reused in the next run.

### Computational Details

All density functional theory (DFT) calculations are carried out using the VASP 6.3^61,62^ (Vienna Ab initio Simulation Package). The Perdew-Burke-Ernzerhof (GGA-PBE) functional is adopted throughout this manuscript^63^. The total potential of the nucleus and core electrons is simulated by the projected augmented wave (PAW) approach, and the valence wave functions are expanded by plane waves up to a cutoff energy of 400 eV^64,65^. The Brillouin zone is sampled using a 2 × 2 × 1 k-point mesh for self-consistent calculations. To avoid artificial interactions between periodic images in the *z* direction, a 15 Å vacuum layer was applied along the *z* direction. A 5×5 supercell of graphene with vacancies was employed as the substrate for simulating the structure of ND@G support. Spin polarization calculations are always exploited. The dDsC method is used to account for the missing van der Waals interactions in PBE functional^66,67^. The self-consistent field (SCF) energy convergence threshold is set to 1.0 × 10^−5 ^eV. Structure optimizations are conducted until the maximum residual force is less than 0.03 eV/Å. Transition states are obtained by NEB method and then further refined with the DIMER method or CINEB method as implemented in ASE package^68–70^.

To evaluate the Gibbs energy of the catalytic system, we only considered the entropy contributions for gas-phase molecules, and for the adsorbed species on catalyst surfaces, it is assumed that $G\approx {E}^{{DFT}}$, in which ${E}^{{DFT}}$ is the electronic energy of the total system. For the gas phase molecules, the Gibbs free energy at finite temperature and pressure (${\mbox{G}}\left({\mbox{T}},{\mbox{P}}\right)$) was calculated as:

3 $${\mbox{G}}\,\left({\mbox{T}},{\mbox{P}}\right)={{\mbox{E}}}^{{\mbox{DFT}}}+{\triangle {\mbox{G}}}_{{\mbox{corr}}}$$

where ${{\mbox{E}}}^{{\mbox{DFT}}}$ is the electronic energy calculated by DFT and ${\triangle {\mbox{G}}}_{{\mbox{corr}}}$ is the thermodynamic correction term accounting for the translational and rotational entropy. Considering that current catalytic reactions take place in the liquid phase, to evaluate the Gibbs energy of solvated molecules, Raoult’s law is exploited. The saturated vapor pressure of reactant R in its pure state at temperature ${\mbox{T=}}373.15$ K is obtained from tabulated data, and it is labeled as ${{\mbox{p}}}^{*}({\mbox{R}})$. Since the concentration of the reactant R in the liquid solution could be readily calculated as mole fraction ${\mbox{x}}$, according to Raoul’s law and given that the solution is an ideal liquid mixture, the corresponding equilibrium vapor pressure ${\mbox{p}}\left({\mbox{R}}\right)$ of reactant R on the surface of the solution is calculated as

4 $${\mbox{p}}\left({\mbox{R}}\right)={{\mbox{p}}}^{*}\left({\mbox{R}}\right)\times {\mbox{x}}$$

Since the R in solution is in equilibrium with the R in the gas phase with partial pressure of ${\mbox{p}}\left({\mbox{R}}\right)$, the thermodynamic correction term could be expressed as

5 $$\triangle {{\mbox{G}}}_{{\mbox{corr}}}=\triangle {{\mbox{G}}}^{{\mbox{ideal}}-{\mbox{gas}}}({\mbox{T}},{\mbox{p}}\left({\mbox{R}}\right))$$

The right side of the above equation indicates the Gibbs energy (excluding the electronic energy) of molecule R in the temperature of T and partial pressure of ${\mbox{p}}\left({\mbox{R}}\right)$ In this contribution, the concentration of the TOL and MCH is assumed as x = 0.11, and the saturated vapor pressures of TOL and MCH in their pure state are obtained from Williamham, Taylor, et al. as ${{\mbox{p}}}^{*}\left({\mbox{TOL}}\right)=74175$ Pa. ${{\mbox{p}}}^{*}\left({\mbox{MCH}}\right)=98685$ Pa^71^.

### Reporting summary

Further information on research design is available in the Nature Portfolio Reporting Summary linked to this article.

## Supplementary information

Supplementary Information

Reporting Summary

Transparent Peer Review file

## Source data

Source Data

## Data Availability

The data supporting this study are available from the corresponding authors upon request. Source data are provided with this paper.
