# Supplementary material for: RunPd1 fully-exposed clusters for solvent-free hydrogenation of toluene toward hydrogen storage
Source: Nat Commun. 2026 Aug 24;17:10157. doi: 10.1038/s41467-026-75738-7 (PMC13601588; doi:10.1038/s41467-026-75738-7)
Supplement: Supplementary file 1 [file 41467_2026_75738_MOESM1_ESM.pdf]

Supplementary Information

Ru_nPd_1 fully-exposed clusters for solvent-free hydrogenation of toluene toward hydrogen storage

Yue Wang[#,1,2], Hongqiu Chen[#,3], Jiawei Chen[#,2,4], Zhehan Ying [#,5], Lini Yang[*,1], Mi Peng[6], Jingwang Zhang[1], Yang Si[2,4], Jiangyong Diao[2], Geng Sun[*,3], Ding Ma[*,6], and Hongyang Liu[*,2]

[1] Department of Chemistry, Liaoning University, Shenyang, Liaoning 110036, China. 66 Chongshan Road.

[2] Shenyang National Laboratory for Materials Science, Institute of Metal Research, Chinese Academy of Sciences, Shenyang 110016, P. R. China.

[3] Chongqing Key Laboratory of Chemical Theory and Mechanism, School of Chemistry and Chemical Engineering, Chongqing University, Chongqing 401331, P. R. China.

[4] School of Materials Science and Engineering, University of Science and Technology of China, Shenyang 110016, P.R. China.

[5] Materials Characterization and Preparation Facility (GZ), The Hong Kong University of Science and Technology (Guangzhou), Guangzhou, China.

[6] Beijing National Laboratory for Molecular Sciences, College of Chemistry and Molecular Engineering, Peking University, Beijing 100871, P. R. China.

[#] These authors contributed equally: Yue Wang, Hongqiu Chen, Jiawei Chen, and Zhehan Ying

Corresponding Author:

Hongyang Liu (liuhy@imr.ac.cn); Ding Ma (dma@pku.edu.cn); Geng Sun (sungengemail@cqu.edu.cn); Lini Yang (lnyang@lnu.edu.cn)

Supplement Figures and Tables

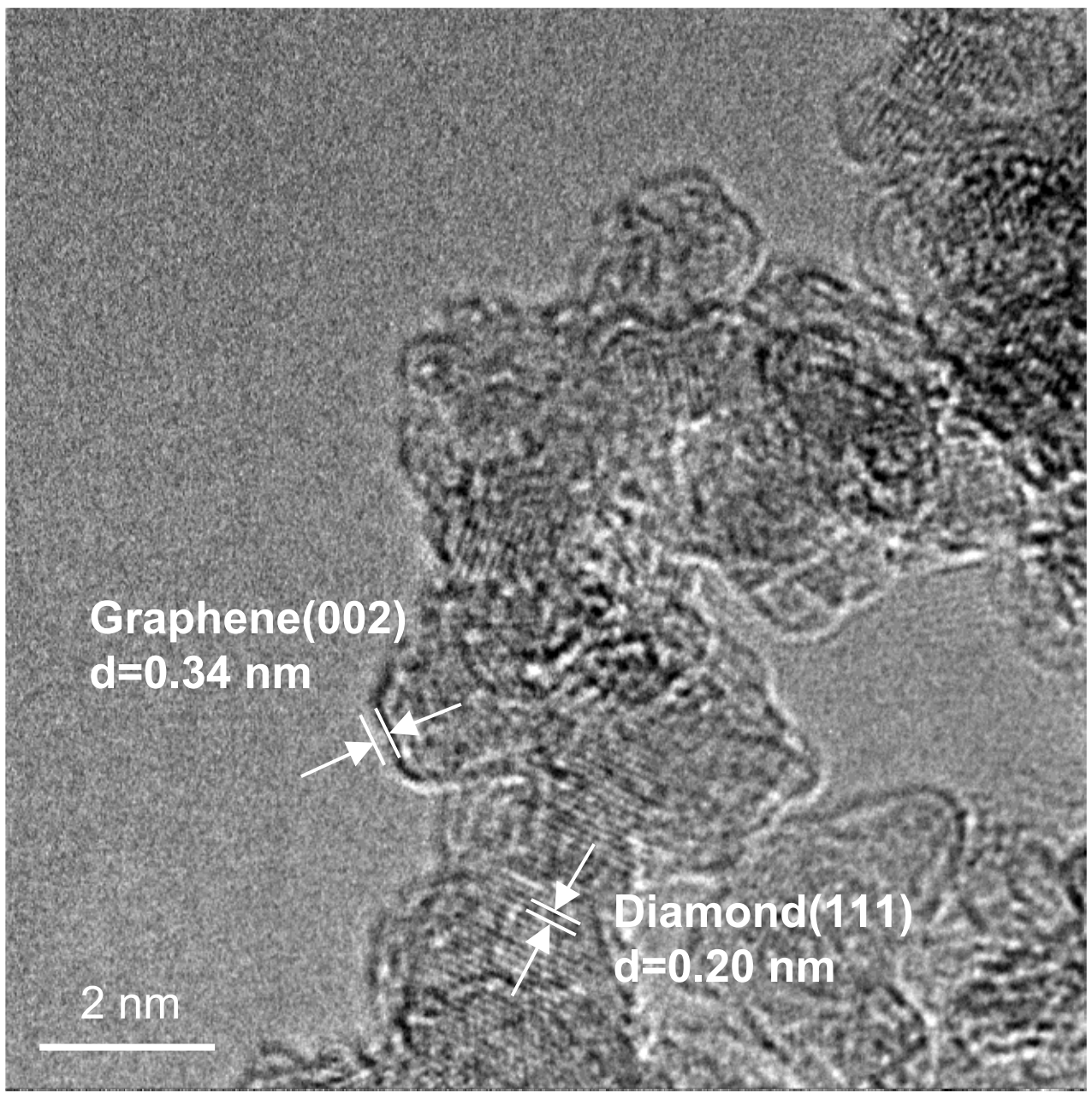

Figure S1. The HRTEM image of the ND@G.

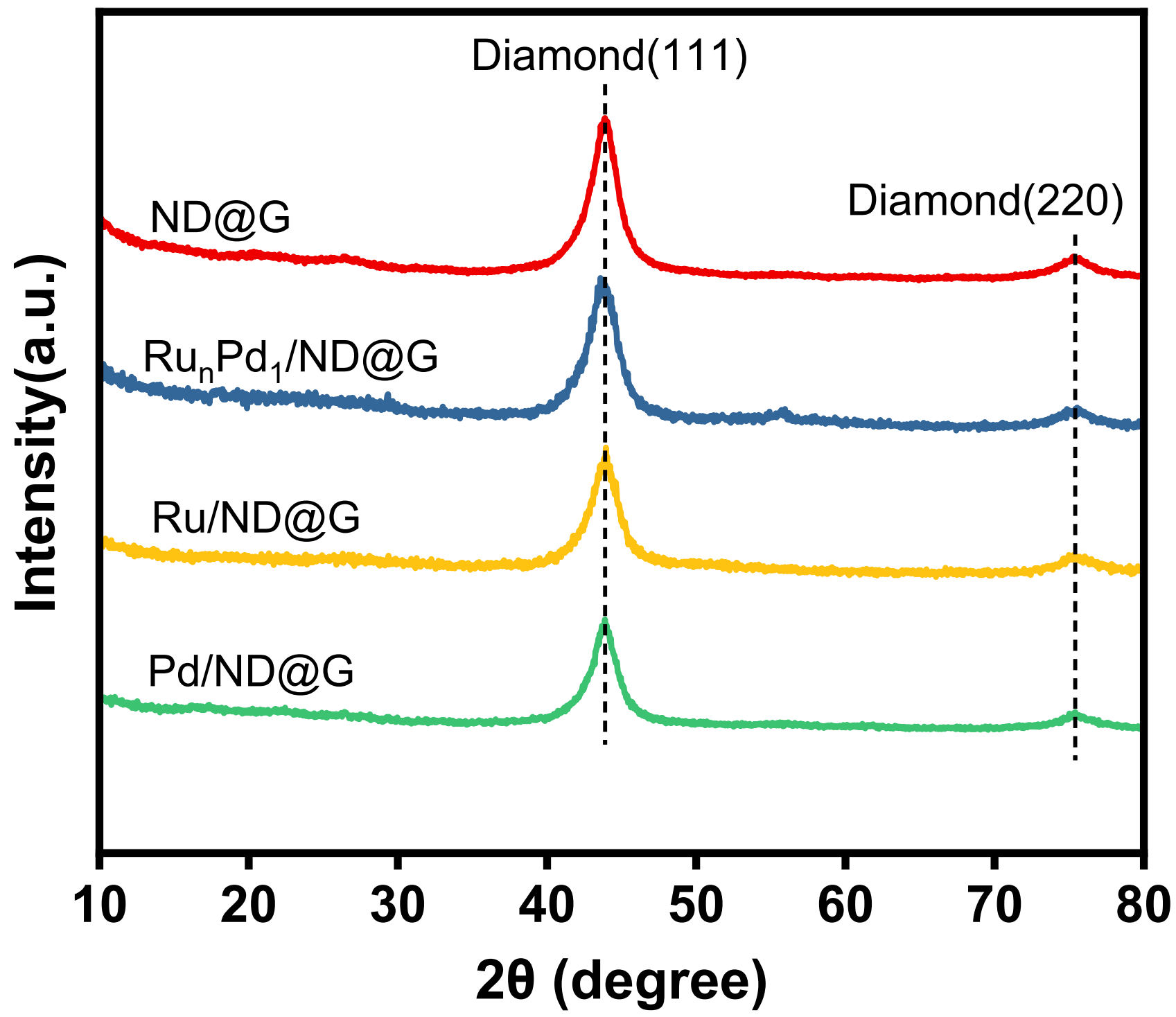

Figure S2. XRD spectra of ND@G, Ru_nPd_1ND@G, Ru/ND@G, Pd/ND@G catalysts.

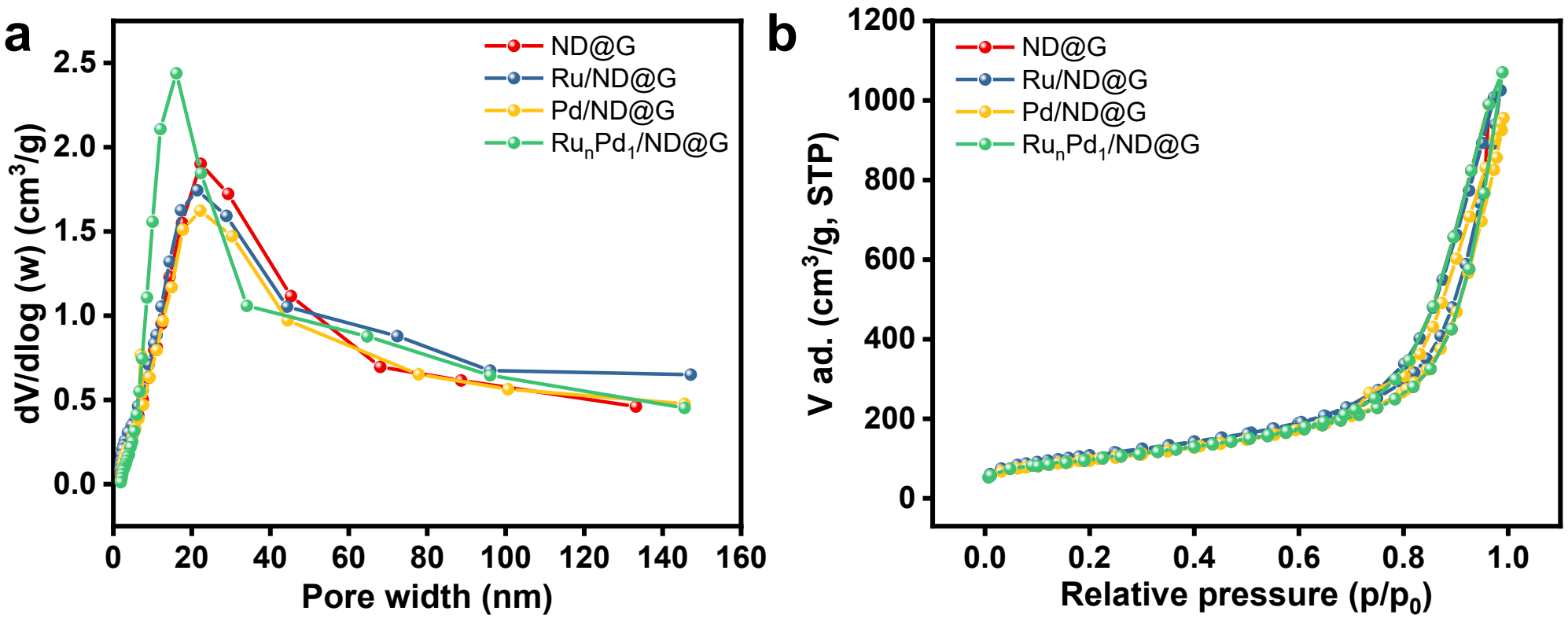

Figure S3. Physicochemical parameters of different samples. a N_2 adsorption/desorption isotherms of different catalysts. **b** Pore size distribution of different catalysts.

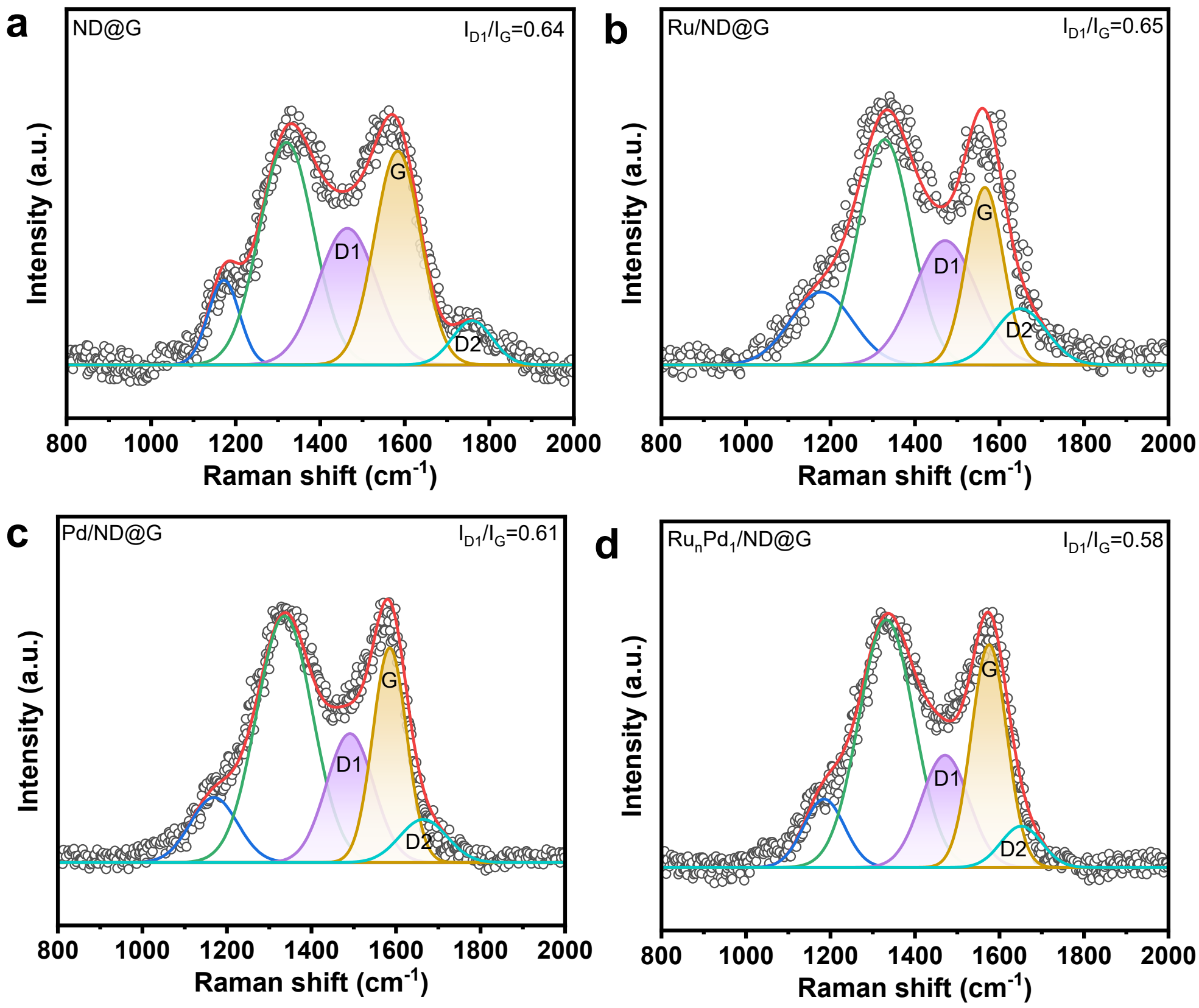

Figure S4. Raman characterizations. Raman spectra of **a** ND@G, **b** Ru/ND@G, **c** Pd/ND@G and **d** Ru_nPd_1ND@G catalysts.

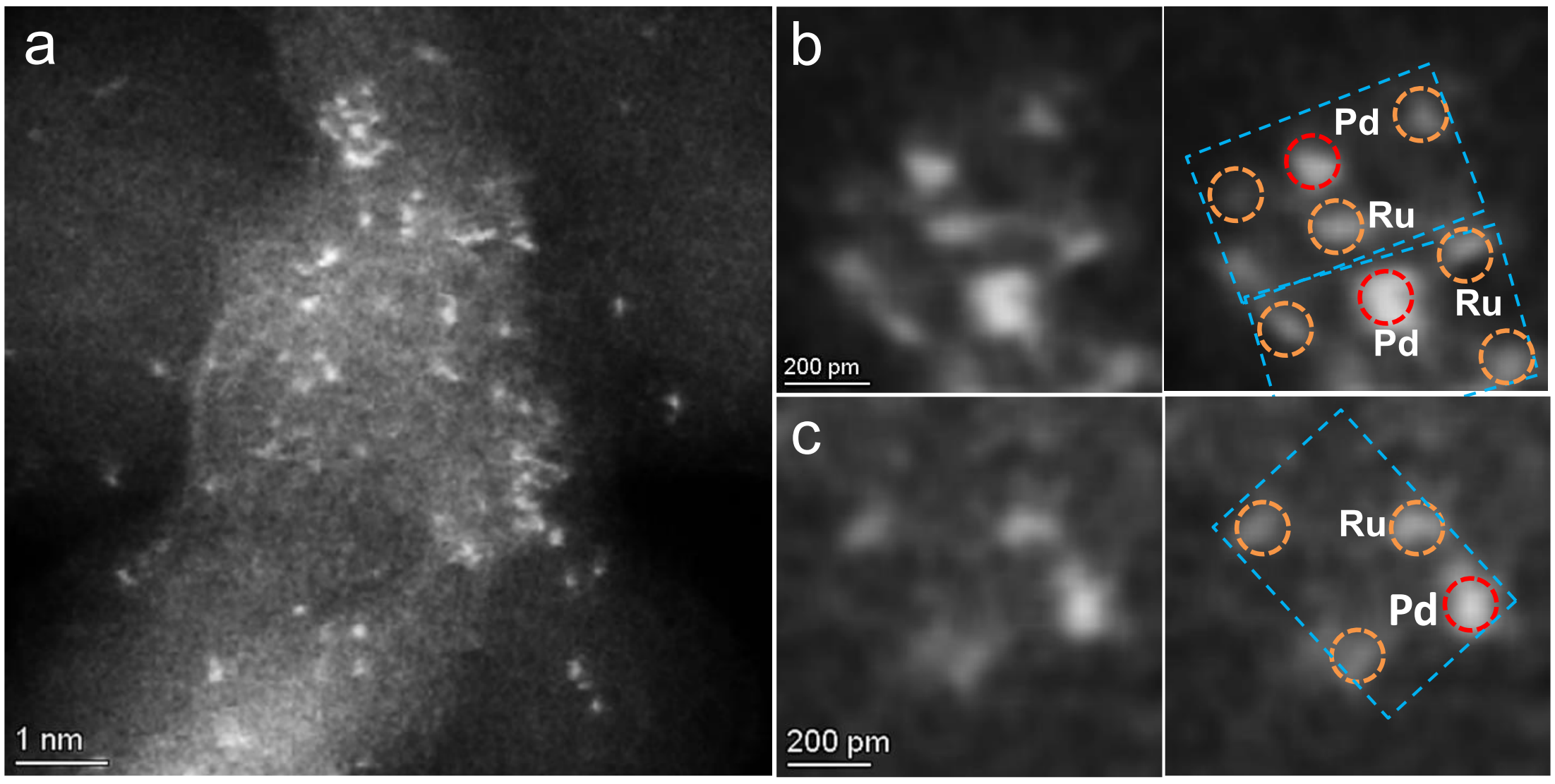

Figure S5. Additional HAADF-STEM image of the Ru_nPd_1/ND@G. **a-c** the high-resolution image of the Ru_nPd_1/ND@G where representative isolated Ru, Pd atoms and Pd_1 assisted fully-exposed Ru cluster catalysts are highlighted by orange, red circles and dashed blue rectangles, respectively.

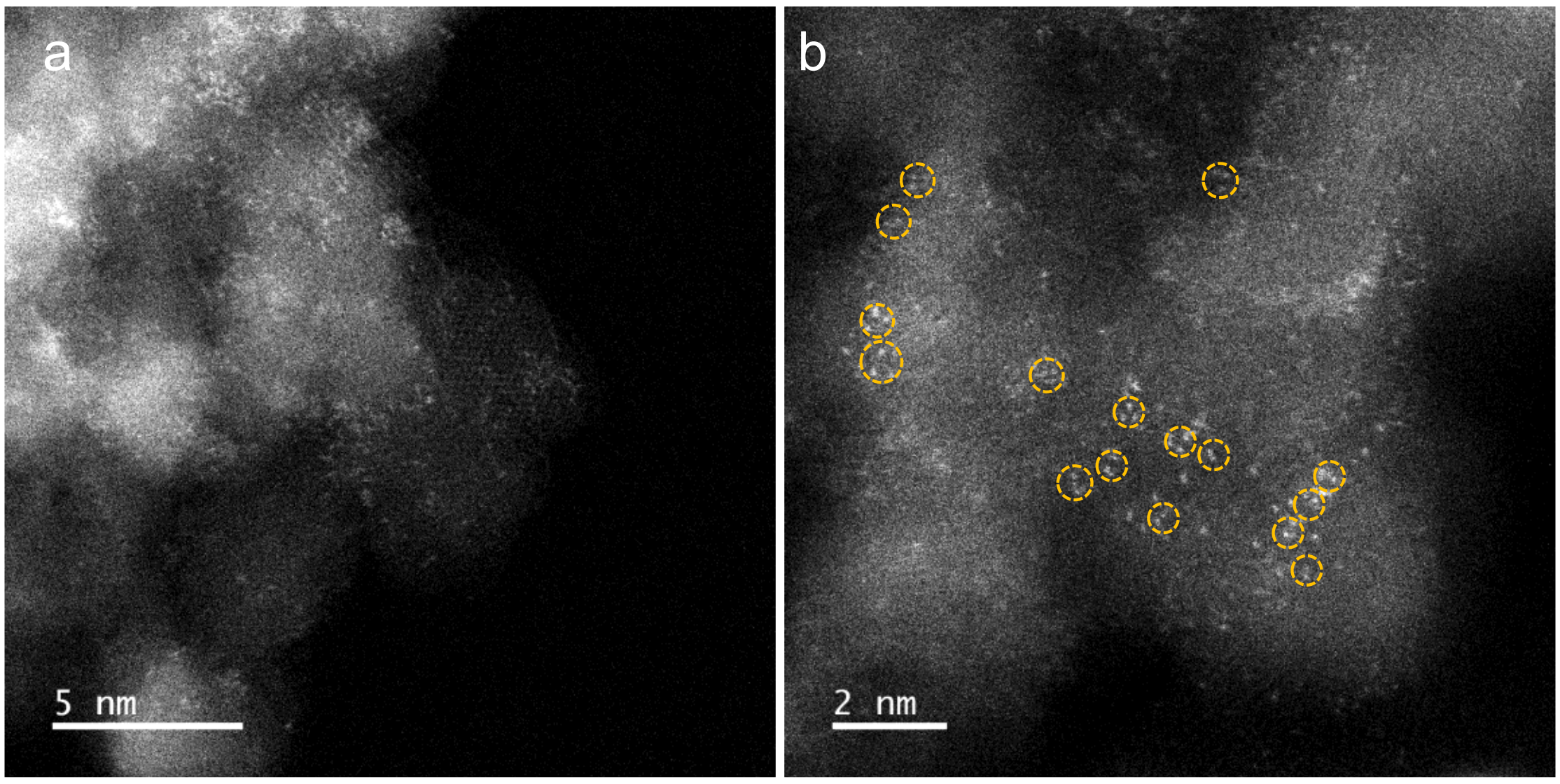

Figure S6. Additional HAADF-STEM images of Ru/ND@G catalyst. a HAADF-STEM image of Ru/ND@G. **b** the high-resolution image of Ru/ND@G, where Ru clusters are highlighted by yellow circles.

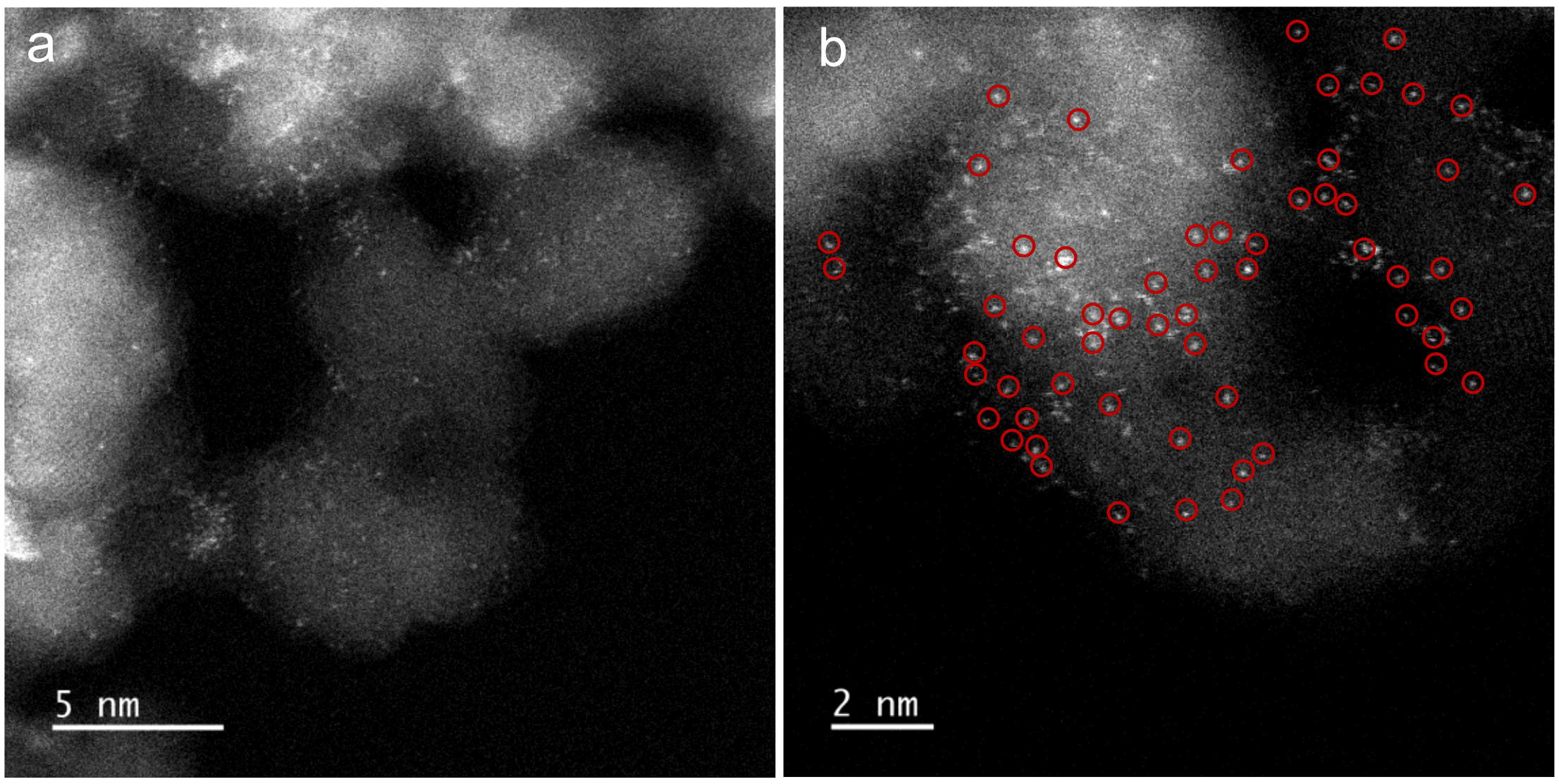

Figure S7. Additional HAADF-STEM images of Pd/ND@G catalyst. a HAADF-STEM image of Pd/ND@G. **b** **t**he high-resolution image of Pd/ND@G, where Pd single atoms are highlighted by red circles.

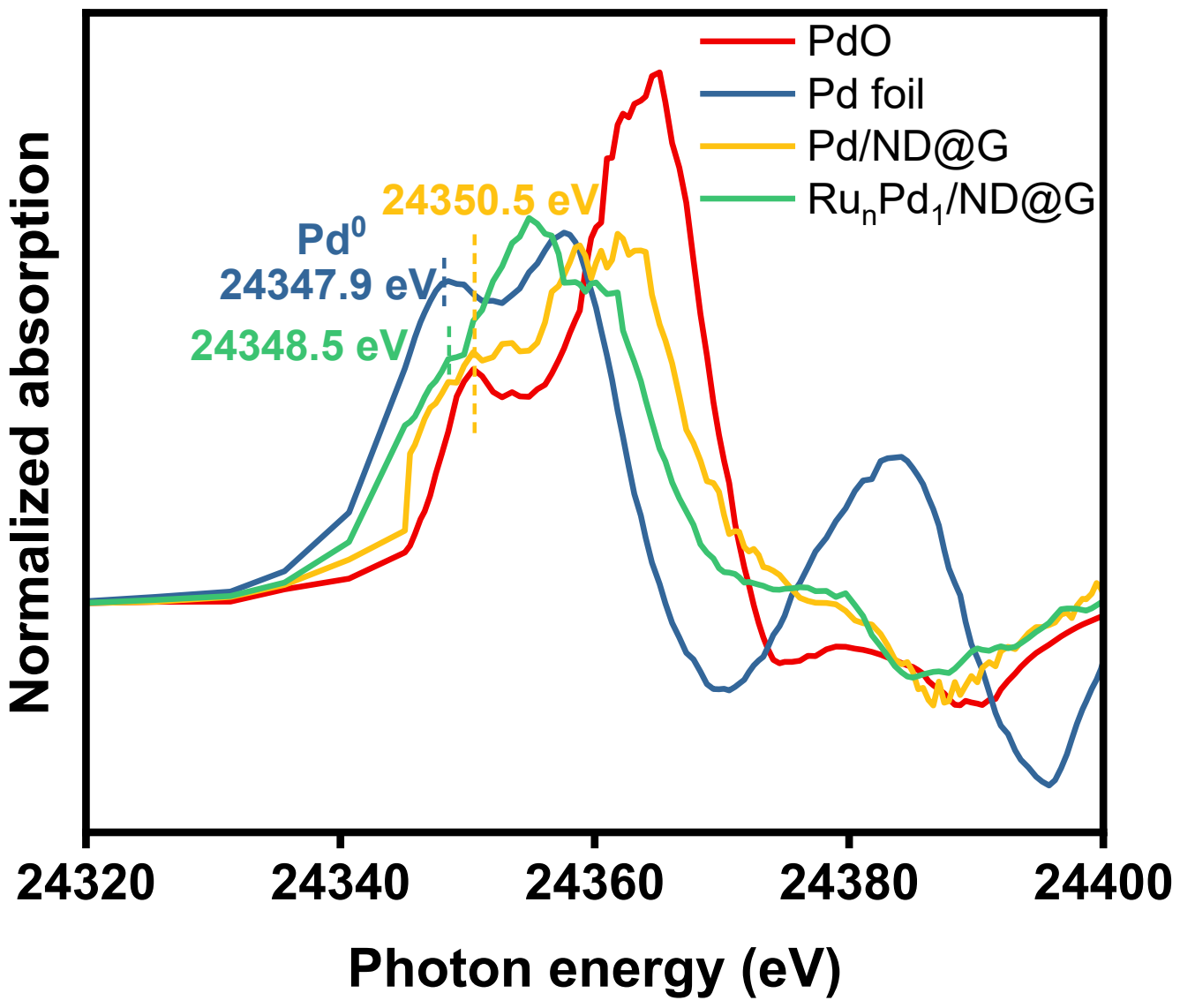

Figure S8. The first derivatives of XANES spectra of Pd/ND@G and Ru_nPd_1/ND@G.

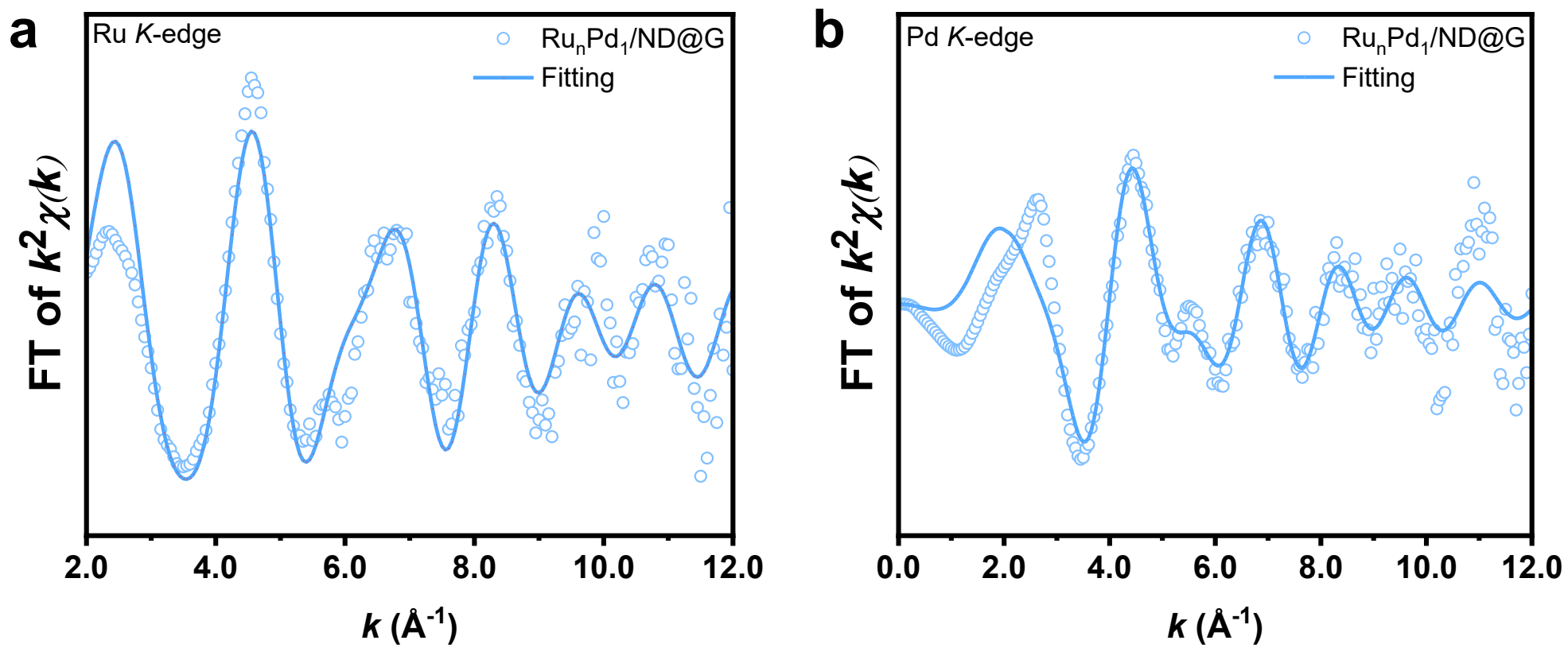

Figure S9. EXAFS fitting results of Ru_nPd_1/ND@G. **a** EXAFS fitting curve of Ru *K*-edge for Ru_nPd_1/ND@G in *k* space. **b** EXAFS fitting curve of Pd *K*-edge for Ru_nPd_1/ND@G in *k* space.

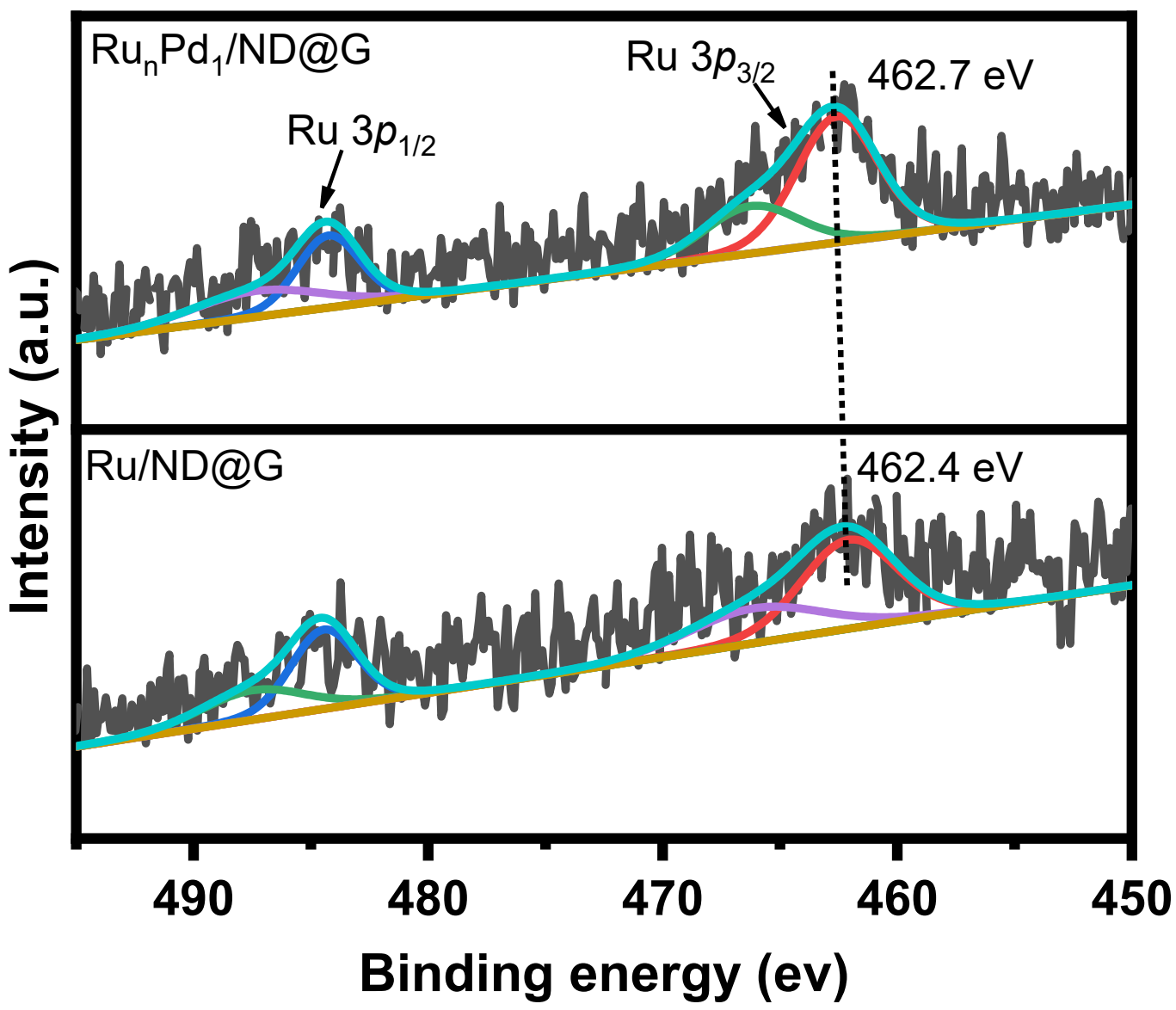

Figure S10. XPS spectra of Ru_nPd_1/ND@G and Ru/ND@G catalysts.

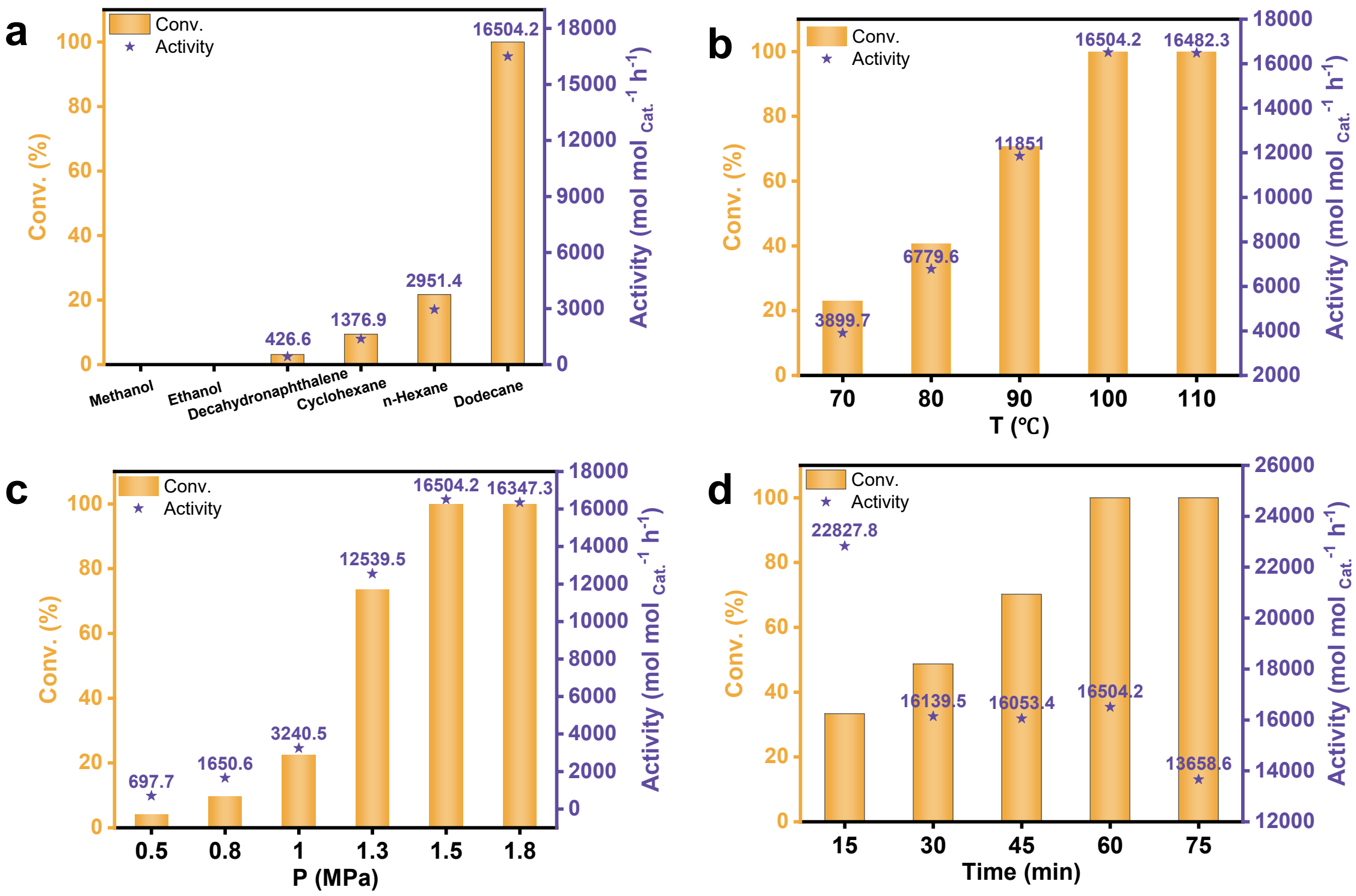

Figure S11. Catalytic performances of Ru_nPd_1/ND@G catalysts. a Catalytic result on Ru_nPd_1/ND@G in various solvents. Reaction conditions: Cat. :10 mg; toluene: 5 mmol, solvent: Dodecane; T=100°C, $P(H_2)$ = 1.5 MPa, t=1 h. Catalytic performances for the hydrogenation of toluene by Ru_nPd_1/ND@G over **b** temperature, **c** pressure and **d** time.

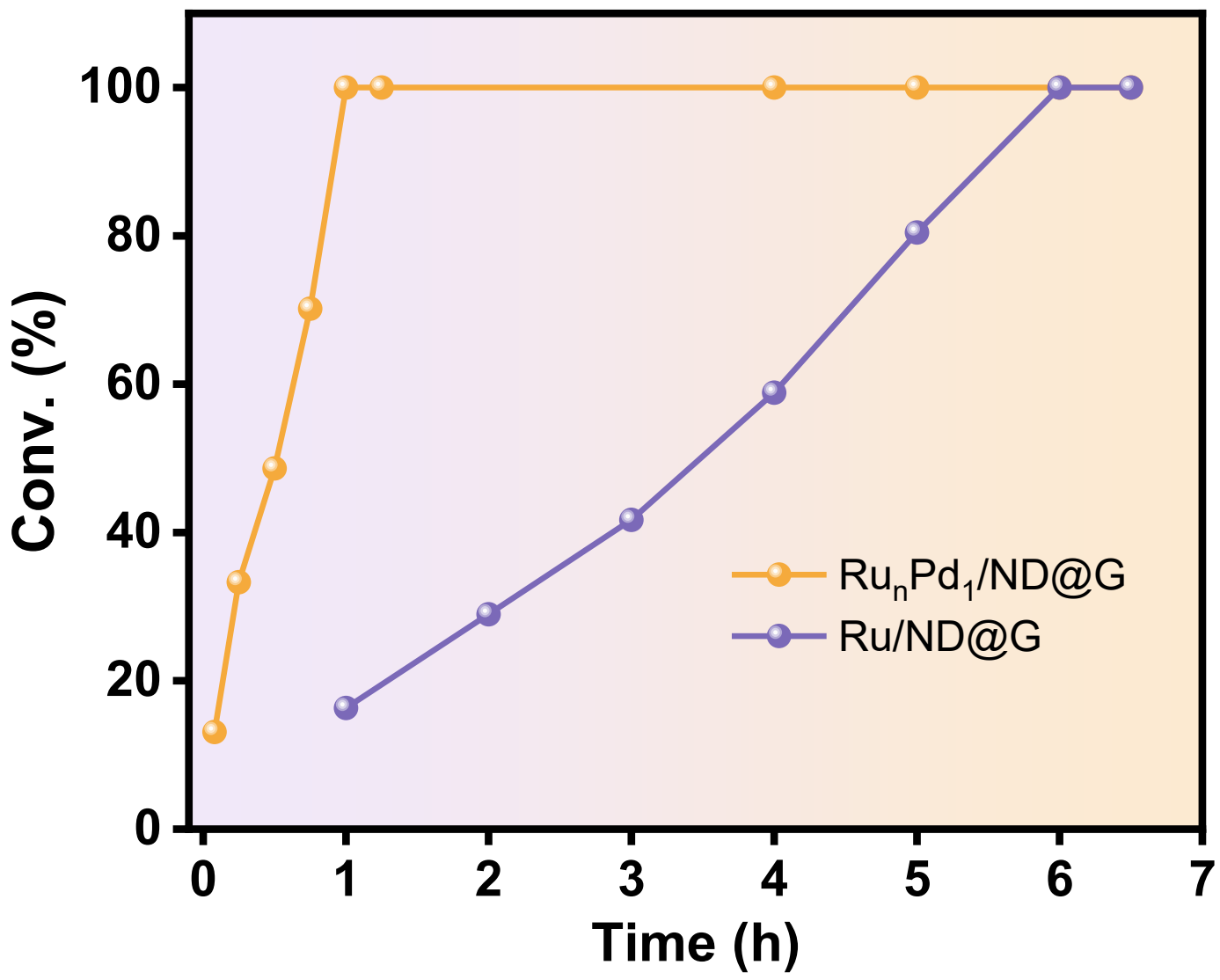

Figure S12. Catalytic performances for the hydrogenation of TOL by different catalysts over time. Reaction conditions: solvent: dodecane 10 mL; TOL: 5 mmol; Catalyst:10 mg; H_2 pressure: 1.5 MPa; temperature: 100°C.

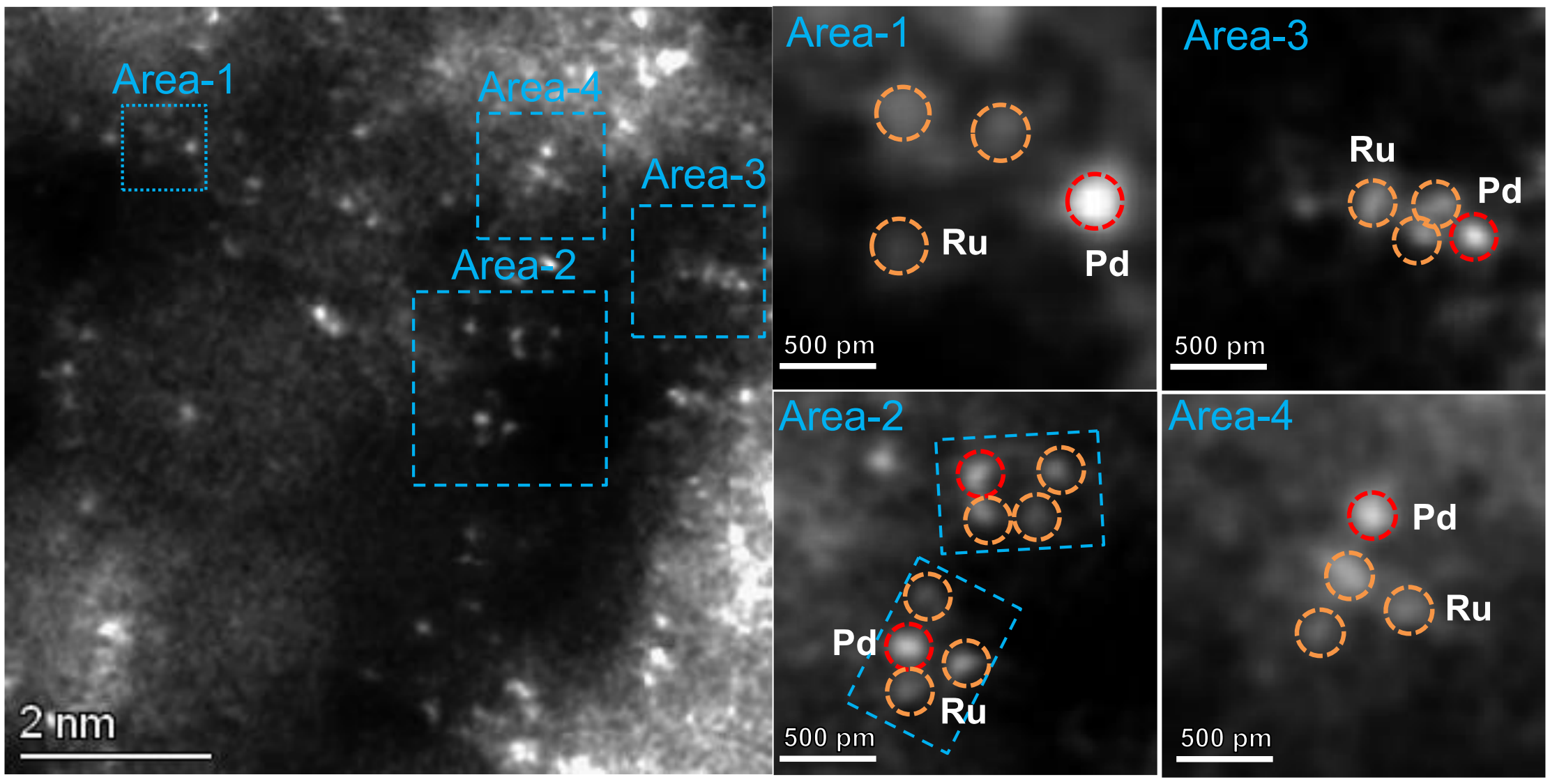

Figure S13. The high resolution HAADF-STEM image of the Ru_nPd_1/ND@G after 5 cycles.

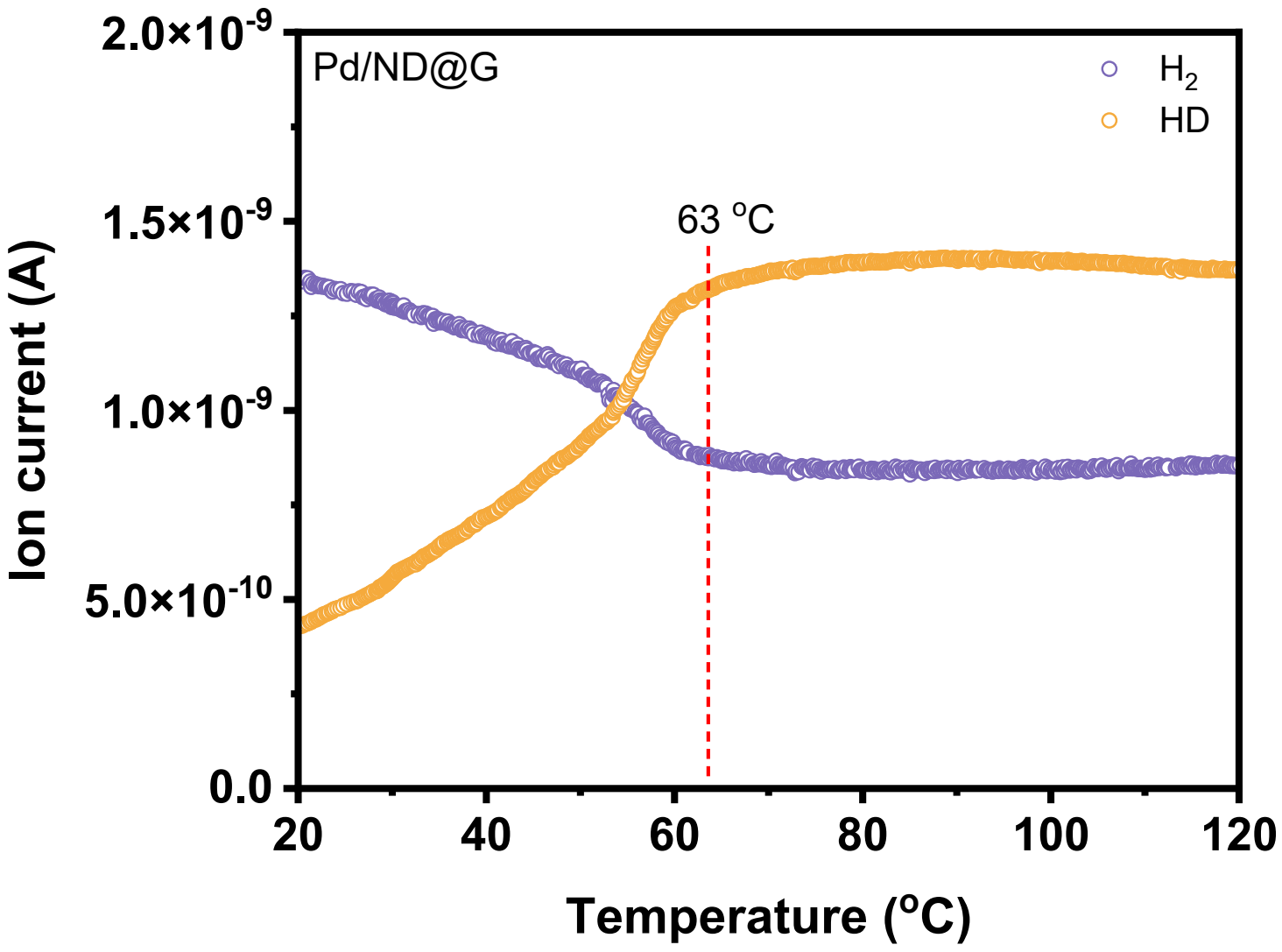

Figure S14. H_2-D_2 exchange experiments for Pd/ND@G.

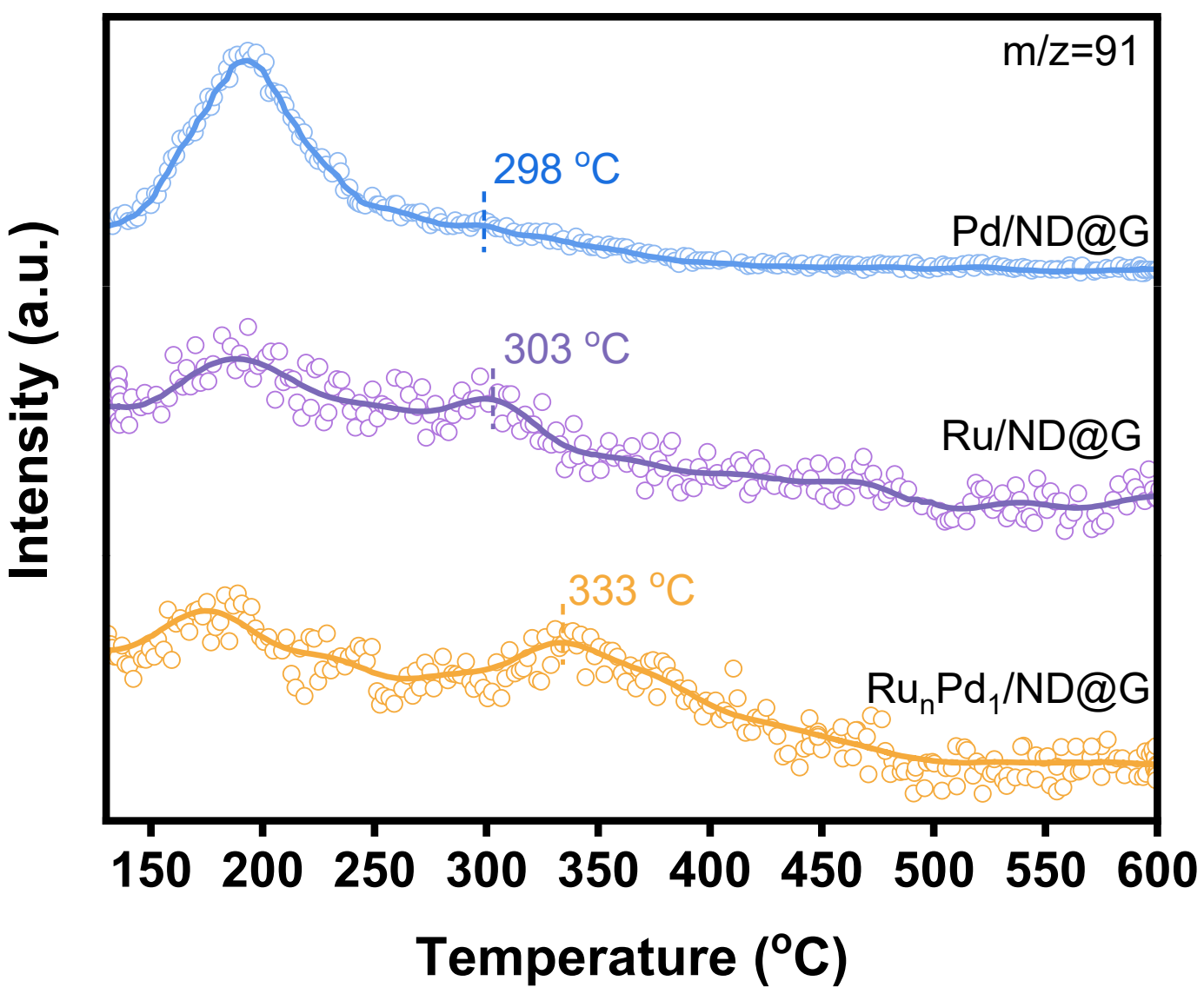

Figure S15. TOL-TPD profiles of the Pd/ND@G, Ru/ND@G and Ru_nPd_1/ND@G catalysts.

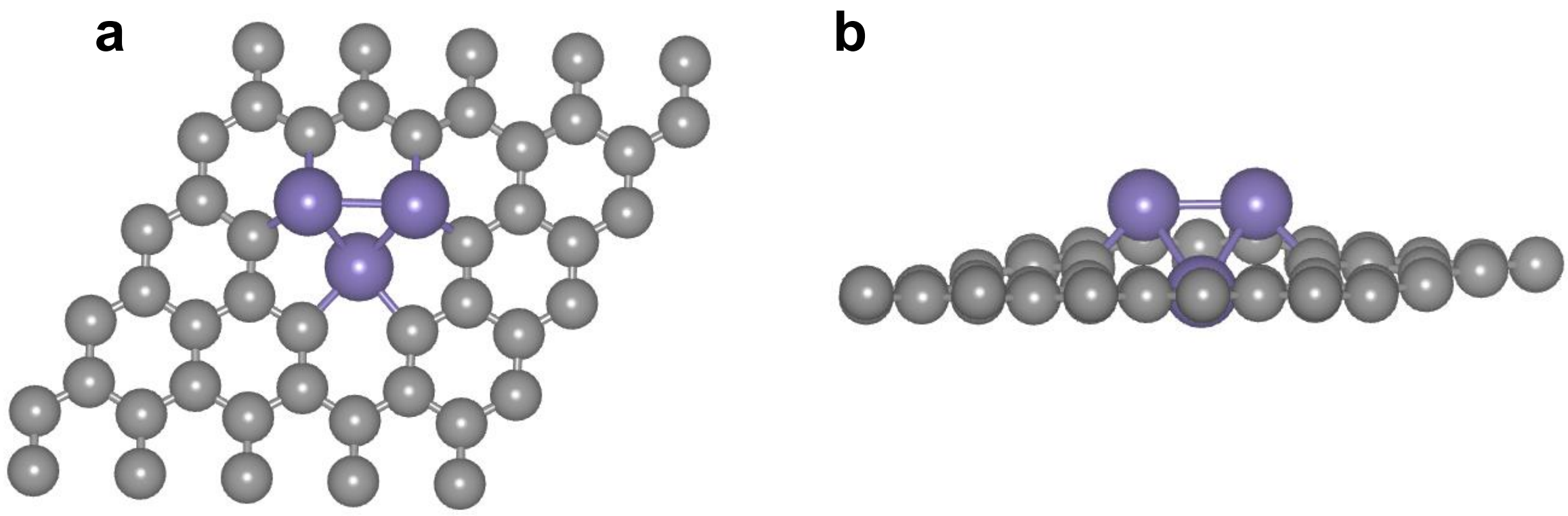

Figure S16. The optimized structures of Ru/ND@G. a The top view of Ru_3/ND@G structures. **b** The side view of Ru_3/ND@G structures. Color code: Ru (purple), C (gray).

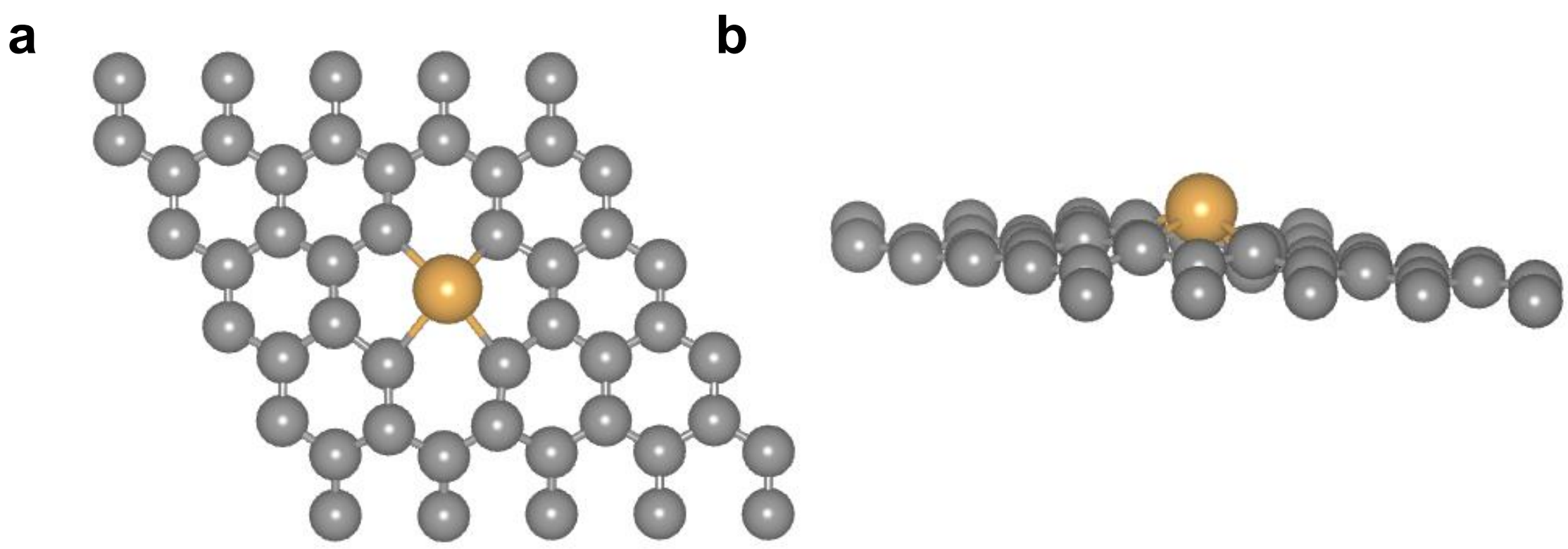

Figure S17. The optimized structures of Pd/ND@G. a The top view of Pd_1/ND@G structures. **b** The side view of Pd_1/ND@G structures. Color code: Pd (yellow), C (gray).

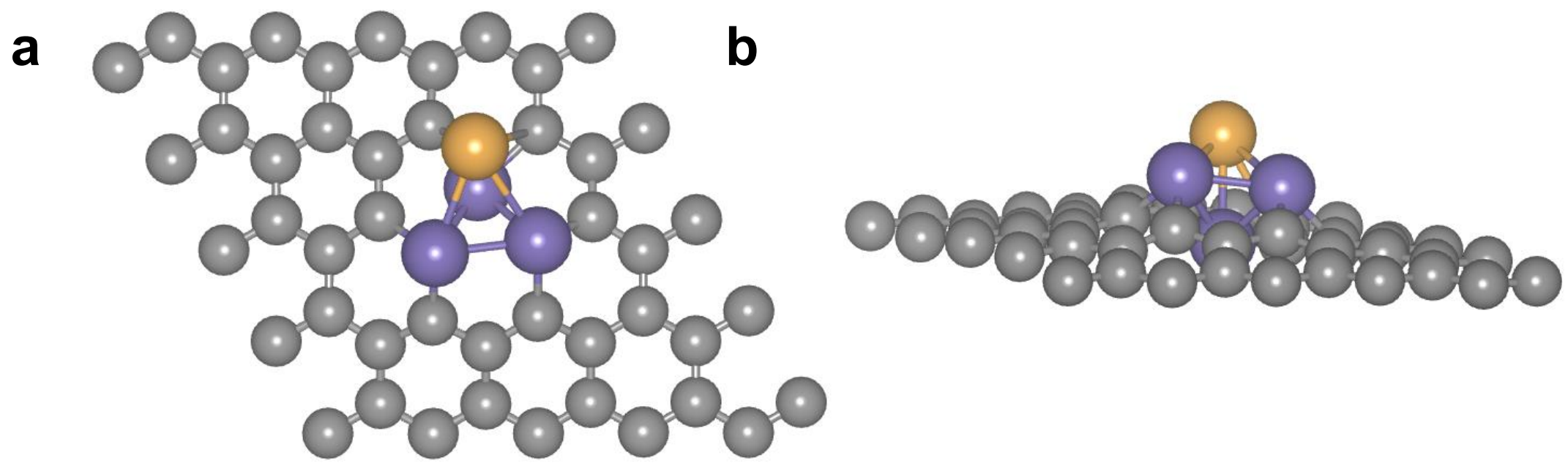

Figure S18. The optimized structures of Ru_nPd_1/ND@G. a The top view of Ru_3Pd_1/ND@G structures. b The side view of Ru_3Pd_1/ND@G structures. Color code: Ru (purple), Pd (yellow), C (gray).

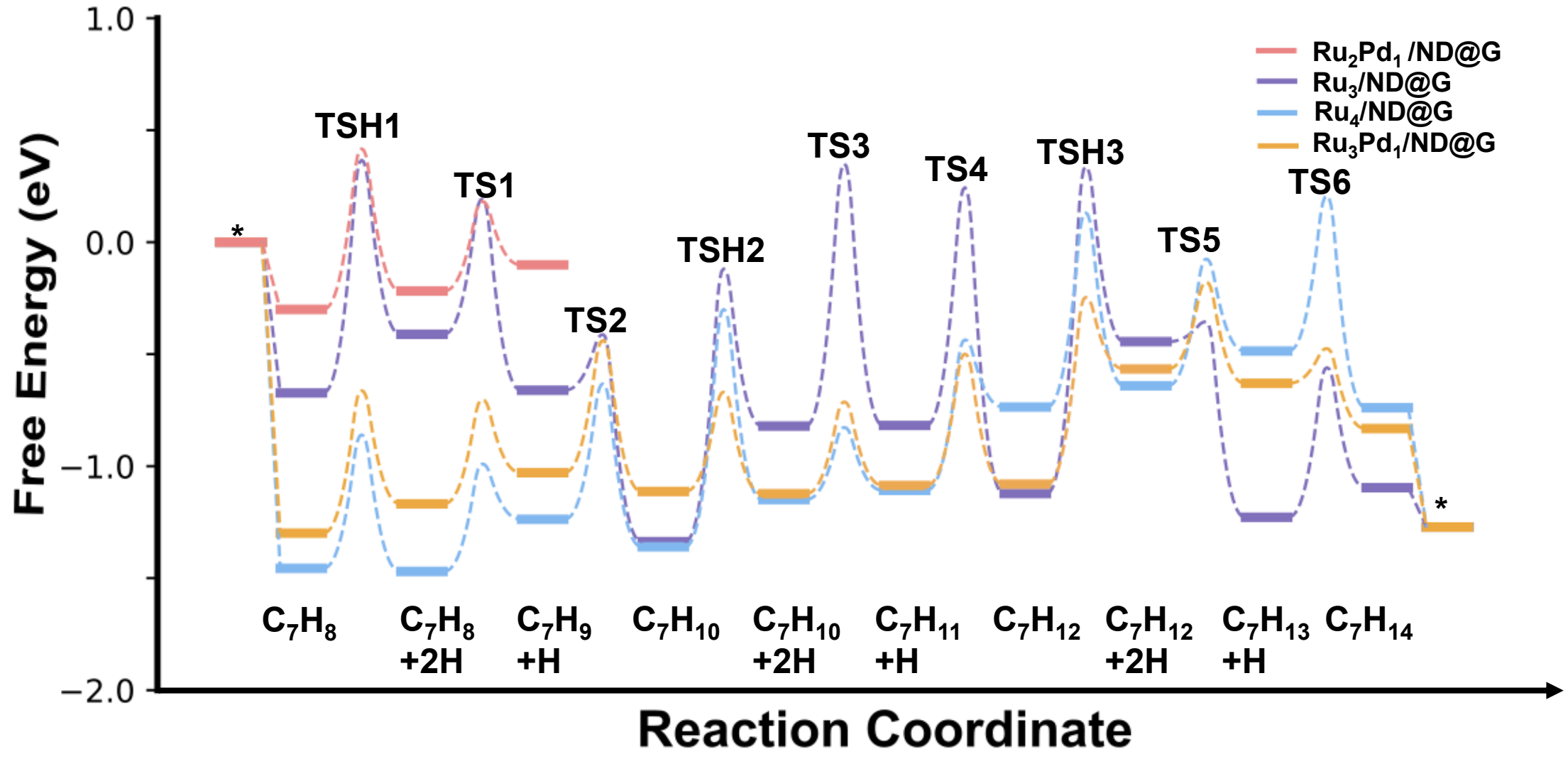

Figure S19. Free energy profiles for TOL hydrogenation to MCH on Ru_2Pd_1/ND@G, Ru_3Pd_1/ND@G, Ru_3/ND@G, Ru_4/ND@G.

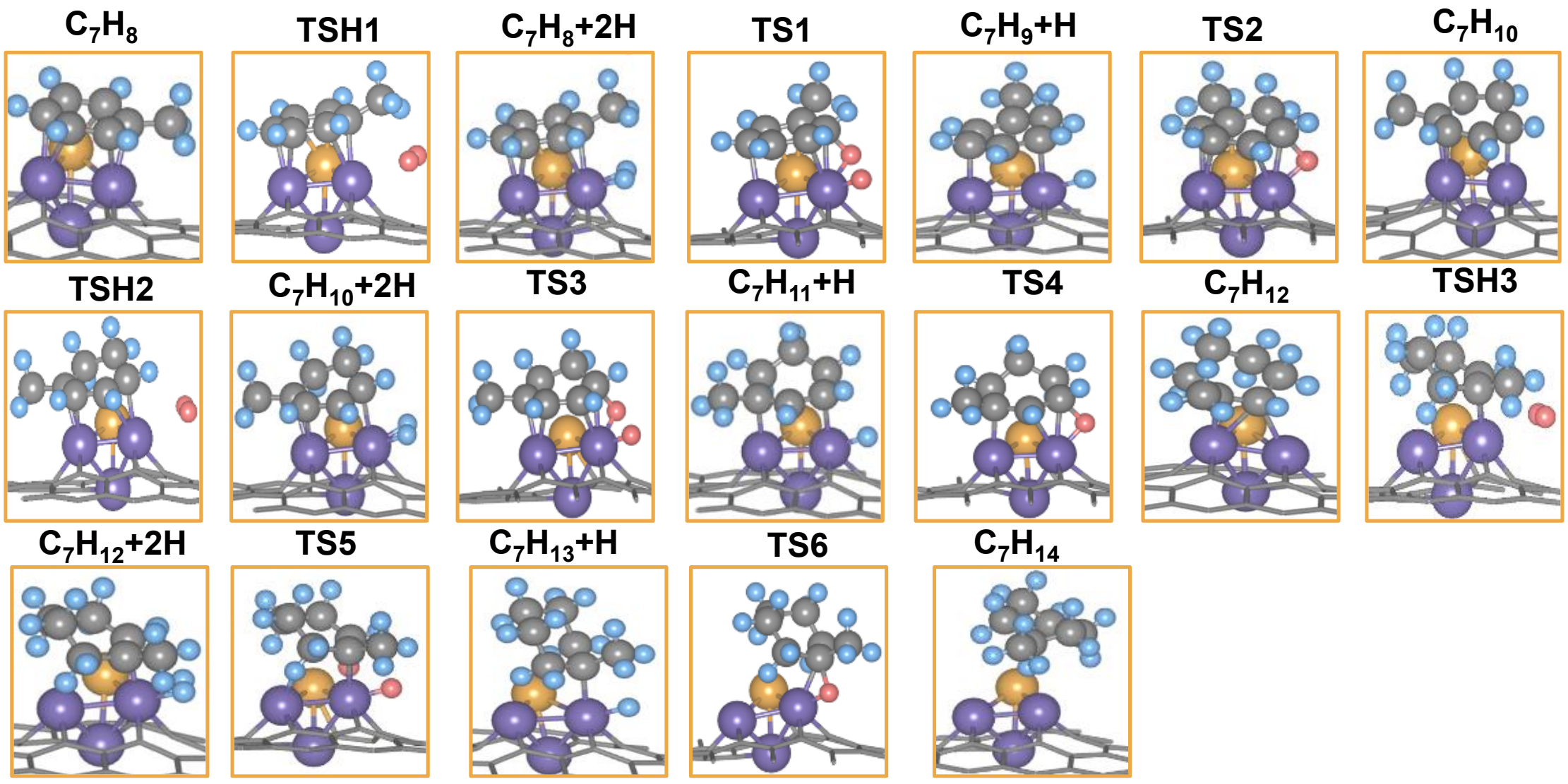

Figure S20. Structure of TOL hydrogenation to MCH on Ru_3Pd_1/ND@G. Carbon atoms in reactant are shown with gray balls, and carbon atoms on graphene are only indicated by lines for simplicity. activating H atoms in red, other H atoms in blue. Ru atoms are shown with purple spheres, and Pd atoms are shown with yellow spheres.

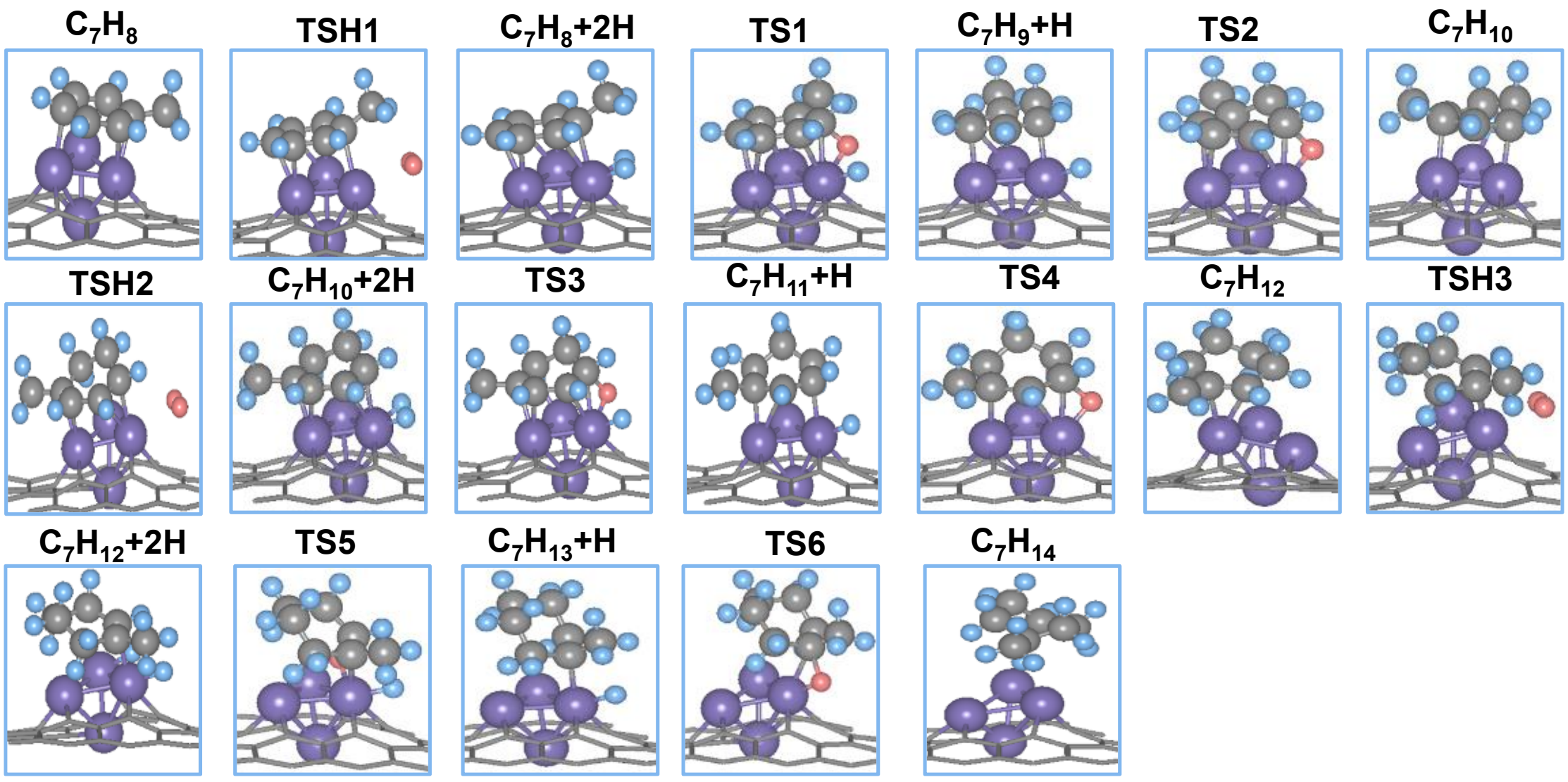

Figure S21. Structure of TOL hydrogenation to MCH on Ru_4/ND@G. Carbon atoms in reactant are shown with gray balls, and carbon atoms on graphene are only indicated by lines for simplicity. activating H atoms in red, other H atoms in blue. Ru atoms are shown with purple spheres, and Pd atoms are shown with yellow spheres.

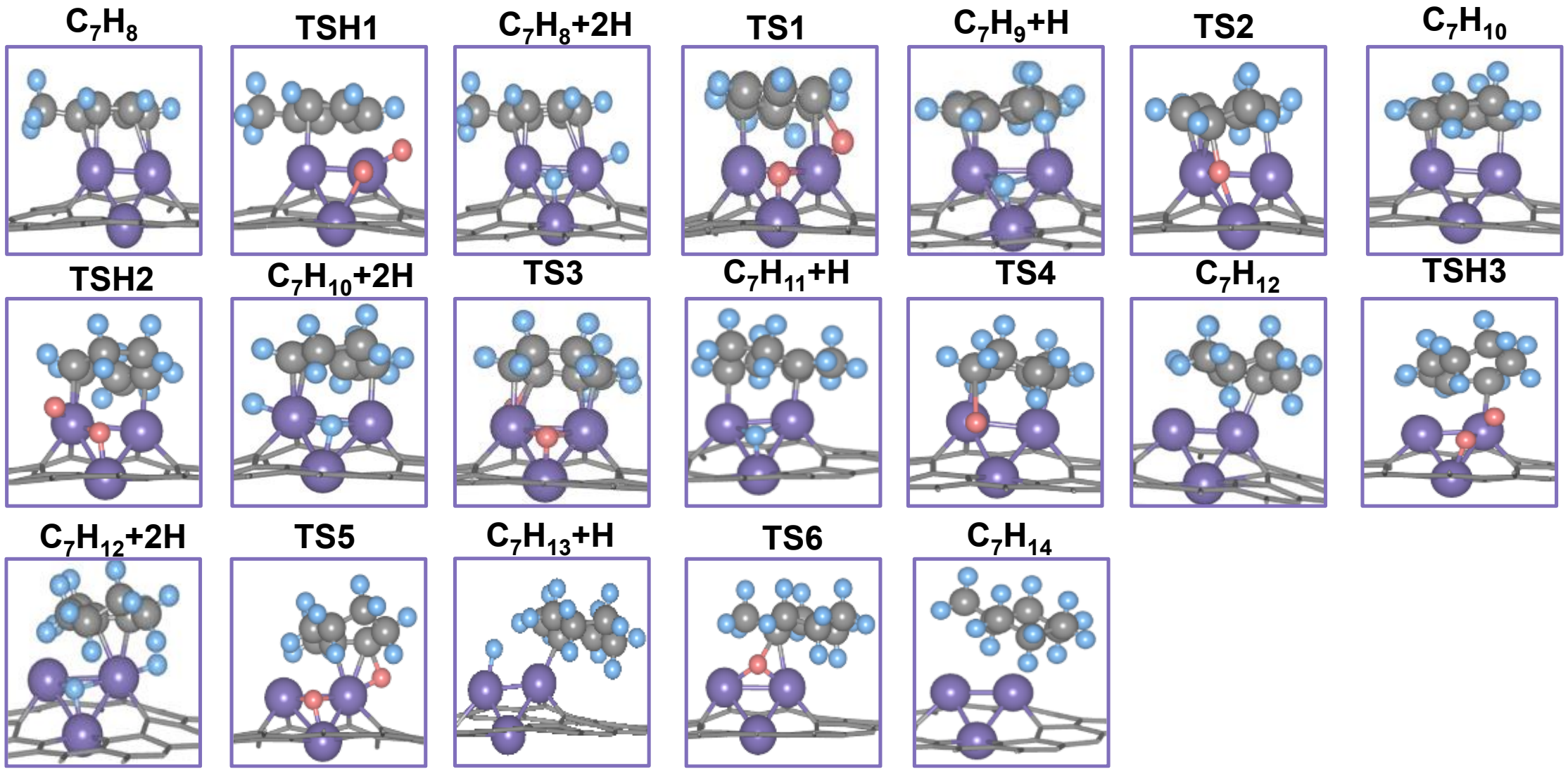

Figure S22. Structure of TOL hydrogenation to MCH on Ru_3/ND@G. Carbon atoms in reactant are shown with gray balls, and carbon atoms on graphene are only indicated by lines for simplicity. activating H atoms in red, other H atoms in blue. Ru atoms are shown with purple spheres, and Pd atoms are shown with yellow spheres.

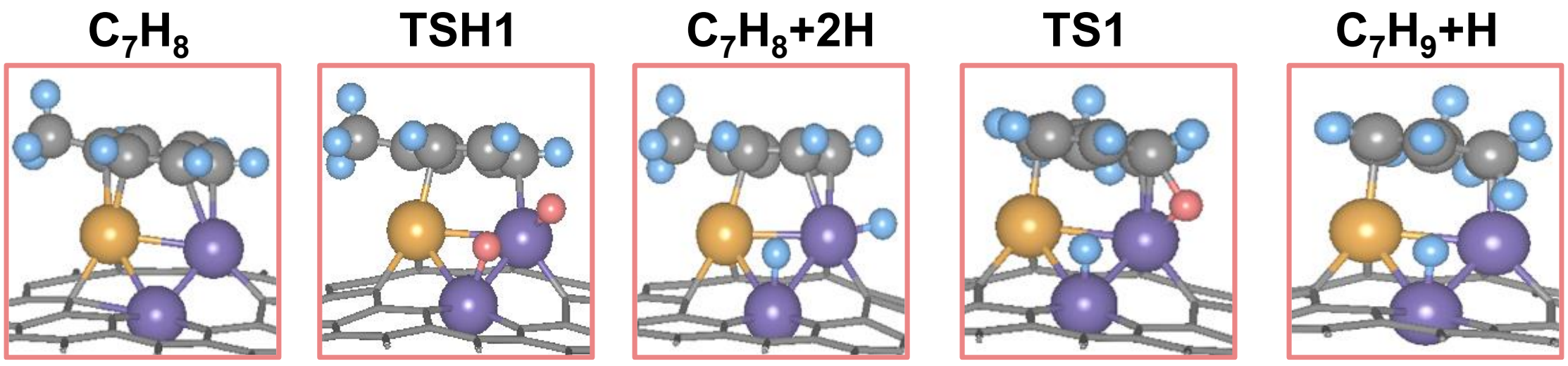

Figure S23. Structure of TOL hydrogenation to MCH on Ru_2Pd_1/ND@G. Carbon atoms in reactant are shown with gray balls, and carbon atoms on graphene are only indicated by lines for simplicity. activating H atoms in red, other H atoms in blue. Ru atoms are shown with purple spheres, and Pd atoms are shown with yellow spheres.

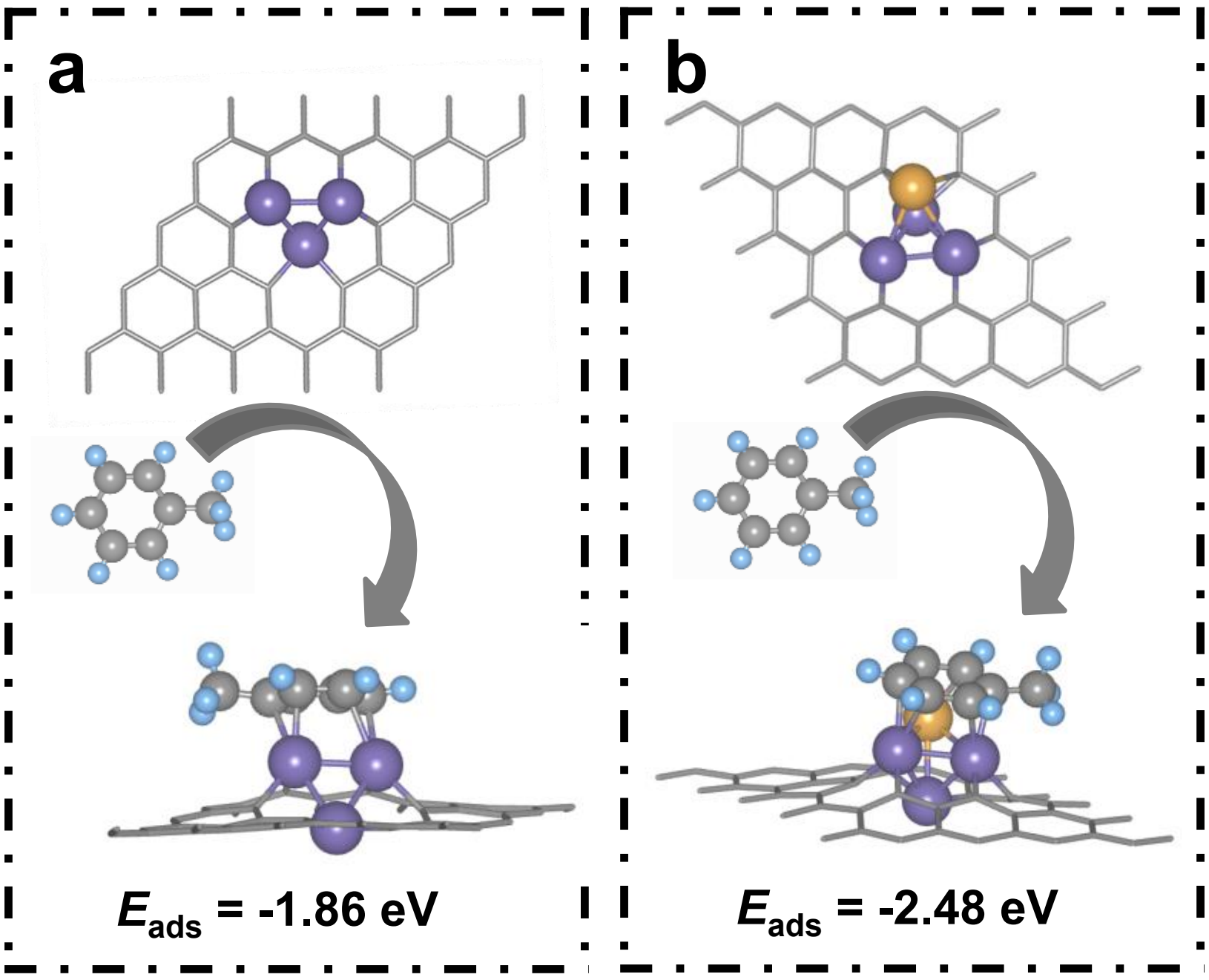

Figure S24. Adsorption energies and structures of toluene on different catalysts. a Ru_3/ND@G and **b** Ru_3Pd_1/ND@G models. Color code: Ru (purple), Pd (yellow), C (gray), H (blue).

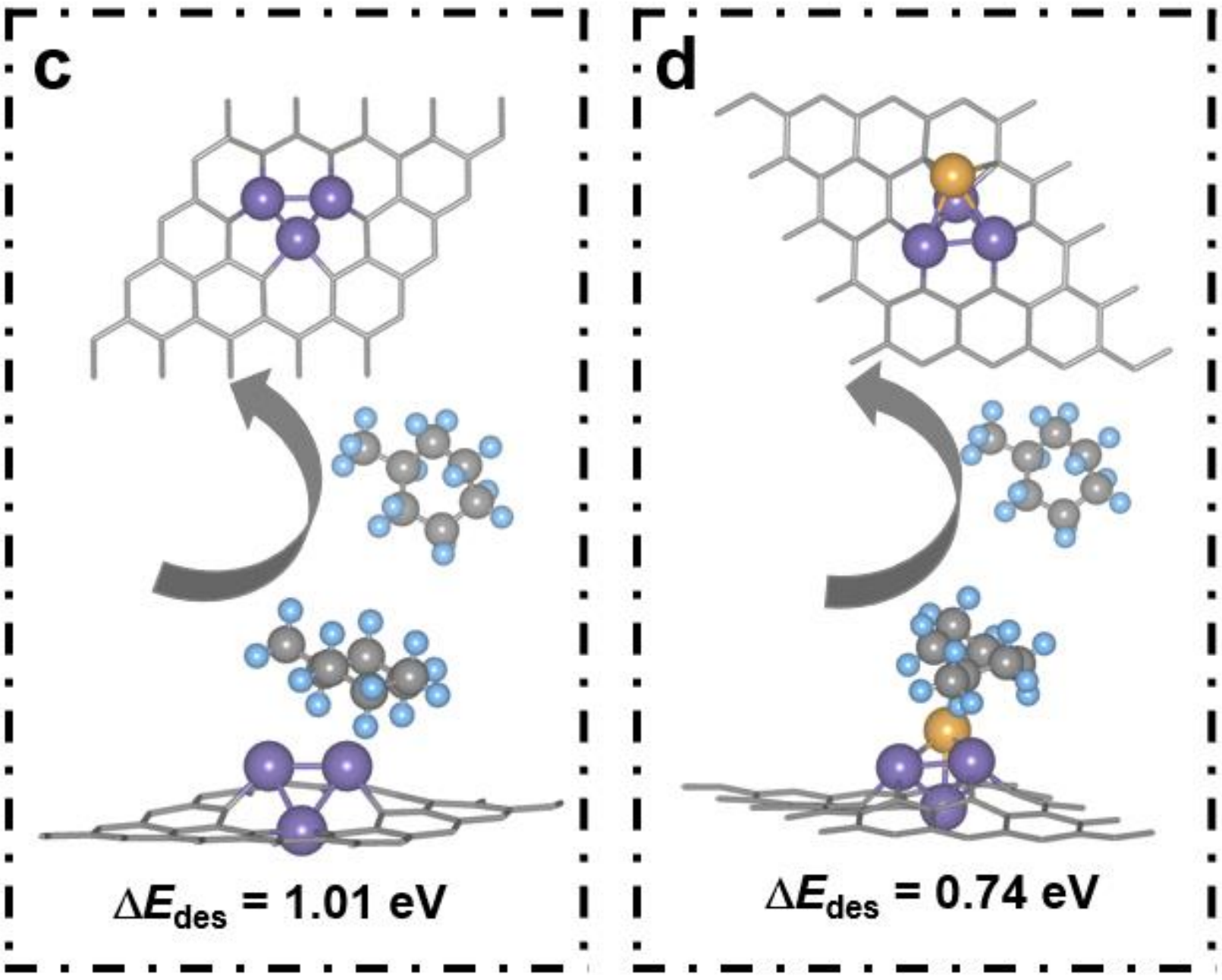

Figure S25. Desorption energies and structures of methylcyclohexane on different catalysts. a Ru_3/ND@G and **b** Ru_3Pd_1/ND@G models. Color code: Ru (purple), Pd (yellow), C (gray), H (blue).

Table S1. Physicochemical parameters of different catalysts.

**Sample**	**Ru loading**[a]	**Pd loading**[a]	S_{BET}[b] [m^2 g^{-1}]	V_{pore}[c] [cm^3 g^{-1}]	**Pore size**[d] [nm]
ND@G	—	—	366.6	1.51	16.5
Ru/ND@G	0.30	—	332.4	1.50	18.1
Pd/ND@G	—	0.050	334.3	1.62	18.6
Ru_nPd_1/ND@G	0.29	0.048	354.6	1.65	18.7

[a] Determined by ICP-AES. [b] BET method. [c] Volume of N_2 at $p/p^0 = 0.98$. [d] N_2 desorption.

Table S2. EXAFS parameters of Pd /ND@G, Ru/ND@G and Ru_nPd_1/ND@G catalysts.

Sample	Shell	Bond length (Å) [b]	CN [c]	σ^2 ($Å^2$) [d]	E_0 shift (eV) [e]	R-factor [f]
**Pd/ND@G**	Pd-O/C	1.97±0.01	1.7	0.003±0.002	-2.6±2.1	0.020
	Pd-Pd	2.80±0.01	0.9	0.013±0.005		
**Ru/ND@G**	Ru-O/C	2.03±0.02	3.9	0.009±0.003	3.2±2.2	0.019
	Ru-Ru	2.71±0.02	2.4	0.007±0.003		
**Ru_nPd_1/ND@G**	Pd-O/C	1.94±0.01	2.7	0.014±0.001	-6.5±1.3	0.012
	Pd-Ru	2.64±0.01	2.4	0.012±0.001		
	Ru-O/C	2.08±0.02	3.2	0.007±0.001	7.8±2.5	0.018
	Ru-Ru	2.73±0.02	1.5	0.006±0.002		

[a] The value of the amplitude reduction factor (S_0^2) lines between 0.7 and 1; [b] Bond length is the interatomic distance; [c] CN is the coordination number; [d] σ^2 is Debye-Waller factor (a measure of thermal and static disorder in absorber scatter distance); [e] E_0 shift is edge-energy shift (the difference between the zero kinetic energy value of the sample and that of the theoretical model); [f] R factor is used to value the goodness of the fitting.

Table S3. Catalytic performance over various catalysts.

Catalyst	T (^oC)	P (MPa)	Solvent	TOF (h^{-1})	Ref.
**Ru_nPd_1/ND@G**	**100**	**1.5**	**/**	**35199.5**	**This work**
**Ru_nPd_1/ND@G**	**100**	**1.5**	**Dodecane**	**24735.1**	**This work**
**Ru_n/ND@G**	**100**	**1.5**	**Dodecane**	**9580.3**	**Previous work**
Ru/NHPC	100	1.4	/	15792	1
Ru/CNF-P	100	3	/	14200	2
Ru/C-Silica	110	4	/	9800	3
Ru-OMC	110	4	/	16884	4
Ru/rGO	110	4	/	26000	5
Ru/NFPC	100	1.4	/	8752	6
Ru@C@YSN-500	50	2	/	5270	7
Ru/rp-Al_2O_3	80	3	n-hexane	735	8
Ru(0)/SiO_2	100	3	n-octane	360	9
Ru/PVPy	120	1	THF	69	10
Pd-Ni/SiO_2	150	7	/	11520	11
PdRu/SiO_2	130	2	THF	90	12
Ru/SiO_2	130	2	THF	1073	12
Ru-MWCNT	50	4	Ethanol	100	13
Pt/CeO_2-A-400	100	0.5	Isopropanol	2164	14
PtRh@HMSNs	30	0.1	2-propanol	99.3	15
Co/Al_2O_3	150	4	——	2016	16
$Rh_1Sn_{0.7}/Al_2O_3$	45	0.1	2-propanol	57.6	17

Supplementary References

1. Tang M, *et al.* 3D-interconnected hierarchical porous N-doped carbon supported ruthenium nanoparticles as an efficient catalyst for toluene and quinoline hydrogenation. *Green Chem* **18**, 6082-6090 (2016).

2. Takasaki M, Motoyama Y, Higashi K, Yoon S-H, Mochida I, Nagashima H. Ruthenium Nanoparticles on Nano-Level-Controlled Carbon Supports as Highly Effective Catalysts for Arene Hydrogenation. *Chemistry – An Asian Journal* **2**, 1524-1533 (2007).

3. Su F, Lv L, Lee FY, Liu T, Cooper AI, Zhao XS. Thermally reduced ruthenium nanoparticles as a highly active heterogeneous catalyst for hydrogenation of monoaromatics. *J Am Chem Soc* **129**, 14213-14223 (2007).

4. Lan G, *et al.* Direct Synthesis of Ruthenium-Containing Ordered Mesoporous Carbon with Tunable Embedding Degrees by Using a Boric Acid-Assisted Approach. *ChemCatChem* **6**, 353-360 (2014).

5. Yao KX, Liu X, Li Z, Li CC, Zeng HC, Han Y. Preparation of a Ru-Nanoparticles/Defective-Graphene Composite as a Highly Efficient Arene-Hydrogenation Catalyst. *ChemCatChem* **4**, 1938-1942 (2012).

6. Li M, Tang M, Deng J, Wang Y. Nitrogen-doped flower-like porous carbon materials directed by in situ hydrolysed MgO: Promising support for Ru nanoparticles in catalytic hydrogenations. *Nano Research* **9**, 3129-3140 (2016).

7. Guo M, Lan G, Peng J, Li M, Yang Q, Li C. Enhancing the catalytic activity of Ru NPs deposited with carbon species in yolk–shell nanostructures. *J Mater Chem A* **4**, 10956-10963 (2016).

8. Tang N, Cong Y, Shang Q, Wu C, Xu G, Wang X. Coordinatively unsaturated Al^{3+} sites anchored subnanometric ruthenium catalyst for hydrogenation of aromatics. *ACS Catal* **7**, 5987-5991 (2017).

9. Bianchini C, *et al.* A comparison between silica-immobilized ruthenium(II) single sites and silica-supported ruthenium nanoparticles in the catalytic hydrogenation of model hetero- and polyaromatics contained in raw oil materials. *J Catal* **213**, 47-62

(2003).

10. Fang M, Machalaba N, Sánchez-Delgado RA. Hydrogenation of arenes and N-heteroaromatic compounds over ruthenium nanoparticles on poly(4-vinylpyridine): a versatile catalyst operating by a substrate-dependent dual site mechanism. *Dalton Trans* **40**, 10621-10632 (2011).

11. Yang J*, et al.* Bimetallic Pd-M (M = Pt, Ni, Cu, Co) nanoparticles catalysts with strong electrostatic metal-support interaction for hydrogenation of toluene and benzene. *Molecular Catalysis* **492**, 110992 (2020).

12. Zhao S, Cai J, Hou Q, Zhao D, Shen J. Microcalorimetric adsorption and infrared spectroscopic studies of supported Pd, Ru and Pd–Ru catalysts for the hydrogenation of aromatic rings with carboxyl groups. *Catalysis Science & Technology* **11**, 3070-3083 (2021).

13. Jahjah M, Kihn Y, Teuma E, Gómez M. Ruthenium nanoparticles supported on multi-walled carbon nanotubes: Highly effective catalytic system for hydrogenation processes. *J Mol Catal A: Chem* **332**, 106-112 (2010).

14. Yang Y, Lin X, Tang J, Zhang J, Liu C, Huang J. Supported mesoporous Pt catalysts with excellent performance for toluene hydrogenation under low reaction pressure. *Molecular Catalysis* **524**, 112341 (2022).

15. Li K*, et al.* Hydrogenation of toluene to methyl cyclohexane over PtRh bimetallic nanoparticle-encaged hollow mesoporous silica catalytic nanoreactors. *ACS Omega* **6**, 5846-5855 (2021).

16. Zuo L*, et al.* Highly loaded and dispersed cobalt catalysts for the hydrogenation of toluene with triethylamine. *Ind Eng Chem Res* **58**, 19456-19464 (2019).

17. Zhang H, Wang Y, Wang Z, Xu C, Zhou S. Synthesis of Alumina-Supported RhSn Alloy Nanocatalysts by Using Rh@Sn Core–Shell Nanoparticle Precursors for Toluene Catalytic Hydrogenation. *Ind Eng Chem Res* **62**, 6626-6633 (2023).
