# Supplementary material for: RunPd1 fully-exposed clusters for solvent-free hydrogenation of toluene toward hydrogen storage
Source: Nat Commun. 2026 Aug 24;17:10157. doi: 10.1038/s41467-026-75738-7 (PMC13601588; doi:10.1038/s41467-026-75738-7)
Supplement: Supplementary file 3 [file 41467_2026_75738_MOESM3_ESM.pdf]

nature portfolio

Peer Review File

RunPd1 fully-exposed clusters for solvent-free hydrogenation of toluene toward hydrogen storage

Corresponding Author: Professor Ding Ma

This file contains all reviewer reports in order by version, followed by all author rebuttals in order by version.

Version 0:

Reviewer comments:

Reviewer #1

(Remarks to the Author)
In this paper, the authors reported the synthesis of a novel fully-exposed Ru-Pd bimetallic catalyst and its application to solvent-free hydrogenation of toluene toward hydrogen storage. The as prepared RunPd1/ND@G fully-exposed cluster catalyst exhibits robust catalytic activity in the solvent-free hydrogenation of TOL to MCH. This represents a new class of catalyst for solvent-free toluene hydrogenation. Moreover, the obtained catalytic performance is attractive. Therefore, the paper is potentially suitable for publication in Nature Communications. The manuscript can be accepted after minor revision.
Comments:
1: In the manuscript, Page 5, line 111, the authors stated that “RunPd1/ND@G catalyst was prepared via a facile deposition precipitation method.” It is necessary to compare it with a control catalyst prepared using the conventional impregnation method, to assess which preparation method yields better performance, and then to conclude whether the simplified deposition-precipitation (DP) method is indeed superior.
2: As shown in Figure 2b, the differences in the Pd K-edge XANES spectra are very small. It is suggested that the author should also provide the first-derivative XANES spectra.
3: Toluene hydrogenation is a structure-sensitive reaction. Have you considered the fully exposed Ru clusters with other numbers of Ru atoms as the DFT models? Such as Ru2, Ru4 and Ru2Pd1 etc.
4: In the manuscript, the author used RunPd1/ND@G with a Ru loading of 0.3 wt% and a Pd loading of 0.05 wt%. Could other loading ratios lead to even better catalytic performance? A brief optimization study or discussion would be helpful.

Reviewer #2

(Remarks to the Author)
This is a highly original paper by a top team of researchers demonstrating the benefits of RunPd1 fully-exposed clusters synergy for the hydrogenation of toluene. The wisely fabricated RunPd1/ND@G catalysts and revealed that the introduction of Pd optimize toluene adsorption, facilitate TOL activation and enhances MCH desorption, resulting in the robust performance in the solvent-free hydrogenation process. It is very nice to see that the Pd single atom and fully exposed Ru clusters preserve an outstanding toluene hydrogenation activity under the solvent-free conditions, which has been rationalized with experimental and theoretical calculations. I strongly recommend acceptance of this paper in Nature Communications and request the following revision:
1: The authors should provide more scientific justification for selecting ND@G as the support.
2: In line 239, the author mentioned “after 5 cycles the RunPd1/ND@G catalyst also exhibit stability without significantly activity degradation (Figure 3e)”. The structure of the used catalyst should also be confirmed to be stable.
3: The performances of common reference catalysts (e.g., commercial Ru/C, Pd/C) under identical reaction conditions are not included. Such a comparison would help readers better assess the practical advancement represented by RunPd1/ND@G.
4: In the manuscript, line 293 “The results indicated that the Ru3Pd1/ND@G catalyst deliver better reactant adsorption and the product desorption kinetics” toluene adsorption was not experimentally verified.
5: In the manuscript, line 91 “to efficiently of TOL hydrogenation to MCH using H2” should be corrected to “to achieve efficient TOL hydrogenation to MCH using H2”.

Reviewer #3

(Remarks to the Author)
The authors report a catalyst for the hydrogenation of toluene with mixed Ru and Rd metals on a graphene/nanodiamond support material. The catalyst exhibits impressive turnover frequencies and stability under optimized conditions. The catalyst is well characterized and the proposed structure consisting of small ensembles of Ru and Pd atoms is interesting. I expect the understanding gleaned from this study regarding the catalytic properties of these small ensembles, which are larger than single-atom centers yet smaller than nanoclusters in typical supported metal catalysts, will be valuable to the community.

Nevertheless, there are instances where the evidence provided do not fully support the claims and conclusions. There are also aspects of the analysis and interpretations that are not fully articulated, challenging the reader to follow the flow of logic. I recommend that the authors address these aspects by revising the article according to the major and minor comments below, before the article is reconsidered for publication.

Major comments:

1. Some terms are used without a clear definition, making it challenging to follow the claims made in the article. These include:

a. "…fully exposed cluster catalysts (FECCs) with atomic dispersion and ensemble sites" (line 62) and "fully-exposed Ru cluster" (line 147). I recommend clarifying precisely what characterizes a "fully exposed cluster". For instance, is it a cluster with a specific range of atoms in which all atoms are coordinately unsaturated (i.e., a nanocluster with all metal atoms on the surface)?

b. "…large-scale organic catalysis…" (line 229) and "reaction scale tolerance" (line 234). I recommend clarifying the definition of large-scale and reaction scale, and how the reaction of 100 mmol of TOL reported in Table 1 corresponds to that definition.

2. The interpretations of Figure 1 are hard to follow. Specifically:

a. The authors write that "… RunPd1/ND@G cluster catalyst was well dispersed on the ND@G surface". Here, it is unclear what aspect of the catalyst is well dispersed. From the context, I think this refers to Ru atoms, Pd atoms, or clusters of Ru and Pd atoms. I recommend the authors clarify precisely to avoid misinterpretations.

b. It is not clear what evidence the authors have to assign the blue highlighted boxes in Figure 1c to Ru-Pd clusters. I recommend the authors provide and justify the classification criteria used. For instance, is there an interatomic distance cutoff?

c. It is not clear how it is deduced from the STEM images that these atoms are "fully exposed" and not present as small nanoclusters with some constituent atoms inaccessible to reactants (here, the precise definition of "fully exposed" as mentioned above would be helpful).

3. I recommend that the conditions, conversions, and reaction times for the apparent activation energy measurement (Fig. 3b) be reported. Please clarify if any of the conversions are near 100% (as in Fig. 3a), in which case the rate may be artificially underestimated by normalizing rates by a time that is longer than needed for complete reaction based on the TOF definition in line 399.

4. The authors report in Fig. 3a that the TOF on the RunPd1/ND@G catalyst (35199.5) is ~6-fold larger than on Ru/ND@G (5928.9). In Fig. 3f, however, the TOF for Ru/ND@G is reported to be 9580.3. Please explain this discrepancy.

5. The authors report that the incorporation of Pd into Ru clusters in RunPd1/ND@G improves the performance of the Ru catalyst. While this may be the case when compared with Ru/ND@G catalyst, there are several Ru-based catalysts with similar TOFs, including Ru/rGO, Ru-OMC, etc. Please explain the high activity of these monometallic Ru-based catalysts compared with Ru/ND@G.

6. The authors report that H_2-D_2 exchange occurs at a lower temperature on RunPd1/ND@G than on Ru/ND@G (Fig. 3g-h). This is used as evidence that Pd promotes H_2 activation facilitating hydrogenation (presumably in ensembles with Ru). Yet, Fig. S12 shows that H_2-D_2 exchange occurs at an even lower temperature on Pd/ND@G. This implies that the H_2-D_2 exchange observed on RunPd1/ND@G might be occurring on single-atom Pd sites that are not part of Ru-Pd clusters; thus, the detection of H_2-D_2 exchange at lower temperatures is not evidence that Pd incorporation into clusters with Ru helps to activate H_2 at lower temperatures than an Ru cluster alone. With this context, please clarify the interpretation of these H_2-D_2 exchange data in lines 253-254, specifically how the "introduction of Pd could promoter H_2 activation" for hydrogenation.

7. The authors calculate energies along reaction coordinate using DFT considering an Ru3/ND@G and Ru3Pd1/ND@G cluster. It is not clear how these models help understand the experimentally observed role of Pd in the RunPd1/ND@G catalyst. I recommend firstly that the authors rationalize the choice of these specific structures as representative of active sites in the synthesized catalyst. Then, the authors need to rationalize why one fewer metal atom is included in the Ru model than the RuPd model. The as-prepared Ru3Pd1 model shows a four atom cluster with one Ru embedded in the graphene layer and a 3-fold site exposed that resembles a 3-fold hollow on a FCC(111) surface which interacts strongly with aromatic rings (e.g., J. Phys. Chem. B 2002, 106, 7489-7498). The Ru3 model shows a three-atom cluster with one Ru embedded in the graphene and two Ru exposed at the surface—this does not correspond to a common stable site for aromatic rings. By

making this comparison between two clusters that have not only different speciation (Ru vs Ru/Pd), but also different size and shape, it makes it unclear whether adding Pd is affecting energies simply by adding another atom to expose the right site motif or by electronic differences compared to an Ru atom. To address this concern, I recommend that the authors repeat the calculates with an Ru4 cluster to isolate the electronic effects of Pd.

8. The authors are missing essential computational details, including the method used to calculate vibrational frequencies, the k-point mesh used, vacuum used in z-coordinate, the DFT functional used, the supercell size for the graphene substrate, and energy convergence in self-consistency steps.

Minor comments:

9. There are several sentences with unclear meaning, perhaps due to typographical error. I recommend the authors carefully proofread the article. Some include:
• Line 91: "…solvent to efficiently of TOL…"
• Line 258: "…temperature indicated facilitate MCH adsorption…"
• Line 336: "The ND power powder…"

10. The "Catalytic performances" section (line 191) begins by introducing TOL hydrogenation as a "model reaction" for the RunPd1/ND@G catalyst. Given that the title, abstract, and introduction discuss improvements to TOL hydrogenation as a main motivation for the study, calling it a "model reaction" at this stage is a bit confusing. The authors might consider a different transition sentence to avoid this confusion.

11. The EXAFS spectra were interpreted as including a Ru-O/C coordination. Since the catalyst comprises Ru, Pd, and C, it is not clear where the O is coming from. Please clarify this point.

Version 1:

Reviewer comments:

Reviewer #1

(Remarks to the Author)
The authors have carefully and thoroughly revised the manuscript in response to all of my previous comments and suggestions. Every point I raised has been addressed in a satisfactory manner, and the revisions have notably improved the clarity and quality of the paper. I have no further concerns and am pleased to recommend that the manuscript be accepted for publication in its current form.

Reviewer #2

(Remarks to the Author)
The authors have addressed my concerns. It is now in a suitable form for publication.

Reviewer #3

(Remarks to the Author)
I agree with the changes made by the authors and find that many of the comments from the previous review cycle were very well addressed. I generally find the work to be interesting and compelling. There are three follow-up points that I believe should be addressed to better support the conclusions in the study.

3. I appreciate the clarification on this point. I recommend that the authors specify in the methods section that the conversions were controlled to <20% for the activation energy measurement, and quantify the conversion for the TOF measurement (currently, the text reports "low conversion" without further details).

4. The authors mentioned that "We have revised" the manuscript to explain the TOF data in Fig. 3f are not directly comparable. However, I was not able to find this revision in the highlighted text. Could the authors please specify the revision made?

Furthermore, I see that Table S3 details the original studies and conditions used for the TOF reported in Fig. 3f. I recommend clarifying in lines 256-259 that (i) the TOF on the "other catalysts reported in the literatures" are reported for different conditions/solvents and (ii) that Table S3 details those conditions.

5. I appreciate the point that these catalysts are reported at different conditions making direct comparisons challenging. However, I think some further discussion is warranted, especially comparing the present catalyst to the Ru/rGO catalyst. This catalyst shows a TOF of 26000 under solvent free conditions, which is comparable to the RunPd1/ND@G. The temperature is only 10oC larger, and it is not clear at these high pressures how sensitive rates are to H2 pressures. This would make the point more convincing that the alloying of Pd and Ru is crucial to the catalytic enhancement, rather than the specific size of the Ru cluster exposed on the catalyst.

Open Access This Peer Review File is licensed under a Creative Commons Attribution 4.0 International License, which permits use, sharing, adaptation, distribution and reproduction in any medium or format, as long as you give appropriate credit to the original author(s) and the source, provide a link to the Creative Commons license, and indicate if changes were made.
In cases where reviewers are anonymous, credit should be given to 'Anonymous Referee' and the source.
The images or other third party material in this Peer Review File are included in the article's Creative Commons license, unless indicated otherwise in a credit line to the material. If material is not included in the article's Creative Commons license and your intended use is not permitted by statutory regulation or exceeds the permitted use, you will need to obtain permission directly from the copyright holder.
To view a copy of this license, visit https://creativecommons.org/licenses/by/4.0/

REVIEWER COMMENTS

Reviewer #1 (Remarks to the Author):

In this paper, the authors reported the synthesis of a novel fully-exposed Ru-Pd bimetallic catalyst and its application to solvent-free hydrogenation of toluene toward hydrogen storage. The as prepared RunPd1/ND@G fully-exposed cluster catalyst exhibits robust catalytic activity in the solvent-free hydrogenation of TOL to MCH. This represents a new class of catalyst for solvent-free toluene hydrogenation. Moreover, the obtained catalytic performance is attractive. Therefore, the paper is potentially suitable for publication in Nature Communications. The manuscript can be accepted after minor revision.

We sincerely thank the reviewer for the thorough evaluation of our work. We deeply value your insights and have addressed each concern as follows.

Comments:

1: In the manuscript, Page 5, line 111, the authors stated that "RunPd1/ND@G catalyst was prepared via a facile deposition precipitation method." It is necessary to compare it with a control catalyst prepared using the conventional impregnation method, to assess which preparation method yields better performance, and then to conclude whether the simplified deposition-precipitation (DP) method is indeed superior.

Response: We thank the reviewer for pointing out this issue. We prepared the catalysts via the general impregnation method. All preparation parameters (total metal loading, Ru/Pd ratio) were maintained consistent with DP method to ensure comparability. Under identical reaction conditions, the catalytic performance in toluene hydrogenation was evaluated for both the DP catalyst and the catalyst prepared by conventional impregnation. As shown in **Figure R1**, the Ru-Pd catalyst prepared by the impregnation method exhibited a much lower (5.3%) toluene conversion than that prepared by the DP method. ND@G, composed of the sp^3 diamond core and highly defective sp^2 graphene outer shells. The unique surface with abundant defects can trap and stabilize metal atoms (M=Ru, Pd) via the formation of M-C bonds, which firmly immobilized them and effectively suppresses the migration and agglomeration of metal atoms during subsequent reduction processes.

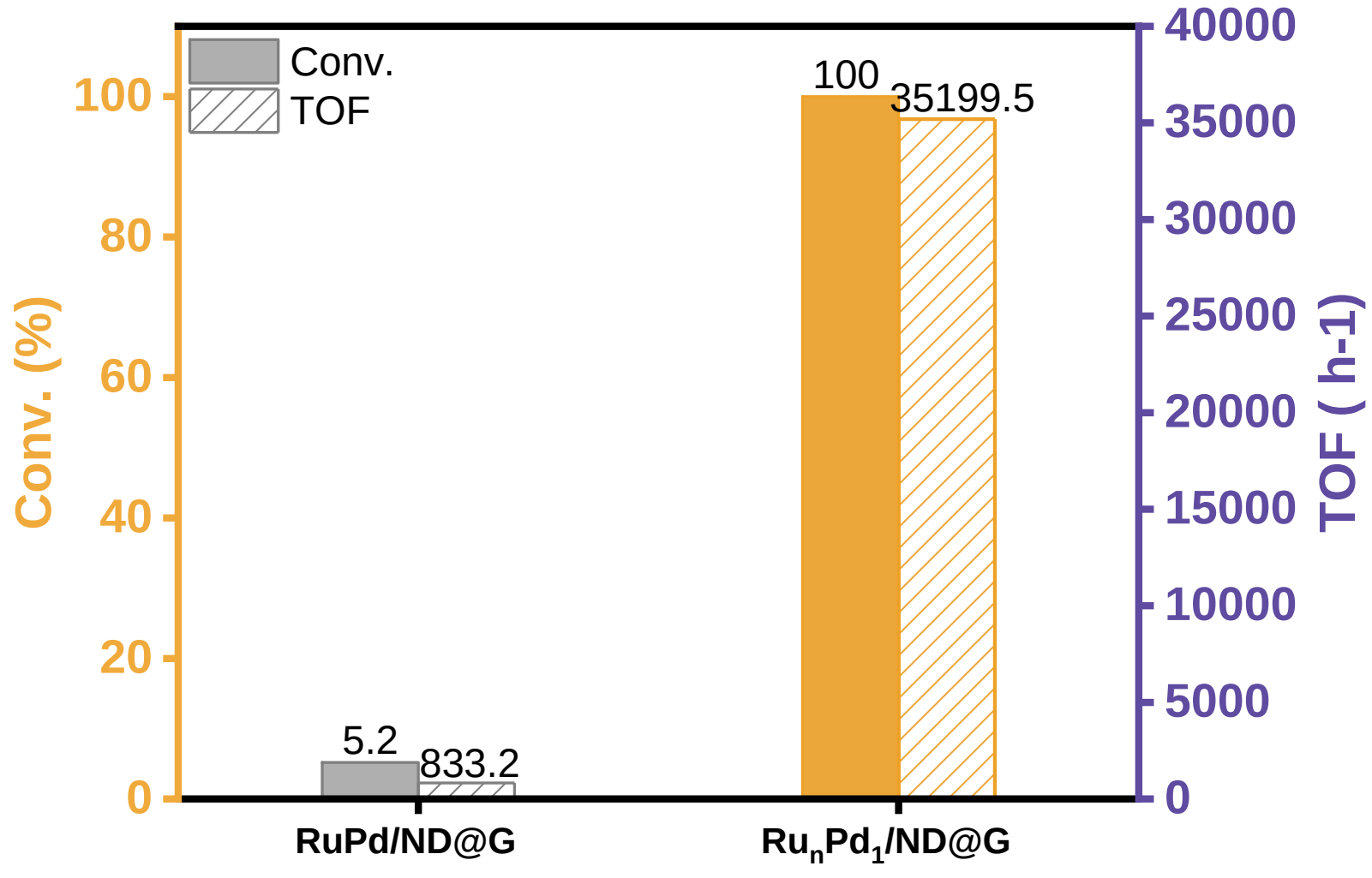

Figure R1 TOF values and TOL conversion (1 h) for the catalysts prepared by conventional impregnation method (RuPd/ND@G) and by deposition-precipitation (Ru_nPd_1/ND@G) under solvent-free conditions. (Reaction conditions: H_2 pressure, 1.5 MPa; temperature, 100 °C; reaction time, 1 h.)

2: As shown in Figure 2b, the differences in the Pd K-edge XANES spectra are very small. It is suggested that the author should also provide the first-derivative XANES spectra.

Response: We are grateful for the suggestion from the reviewer. We add the first-derivative XANES of Pd K-edge (**Figure R2**). The Pd species in Pd/ND@G exhibited the state close to Pd^{2+} valence. In contrast, the Pd species in Ru_nPd_1/ND@G display a lower valence state than that in Pd/ND@G. The results reveal that the electron transfer from Ru to Pd. The Ru_3Pd cluster model was constructed based on the averaged coordination number in EXAFS and HAADF-STEM image analysis. We added and rewrite "According to the differences in the Pd K-edge XANES are very small, the first-derivative XANES spectra was revealed to distinct Pd valence states between the Ru_nPd_1/ND@G and Pd/ND@G catalysts (Figure S8). The Pd species in Pd/ND@G exhibited the state close to Pd^{2+} valence. In contrast, the Pd species in Ru_nPd_1/ND@G display a lower valence state than that in Pd/ND@G." (Please see Line 163-167)

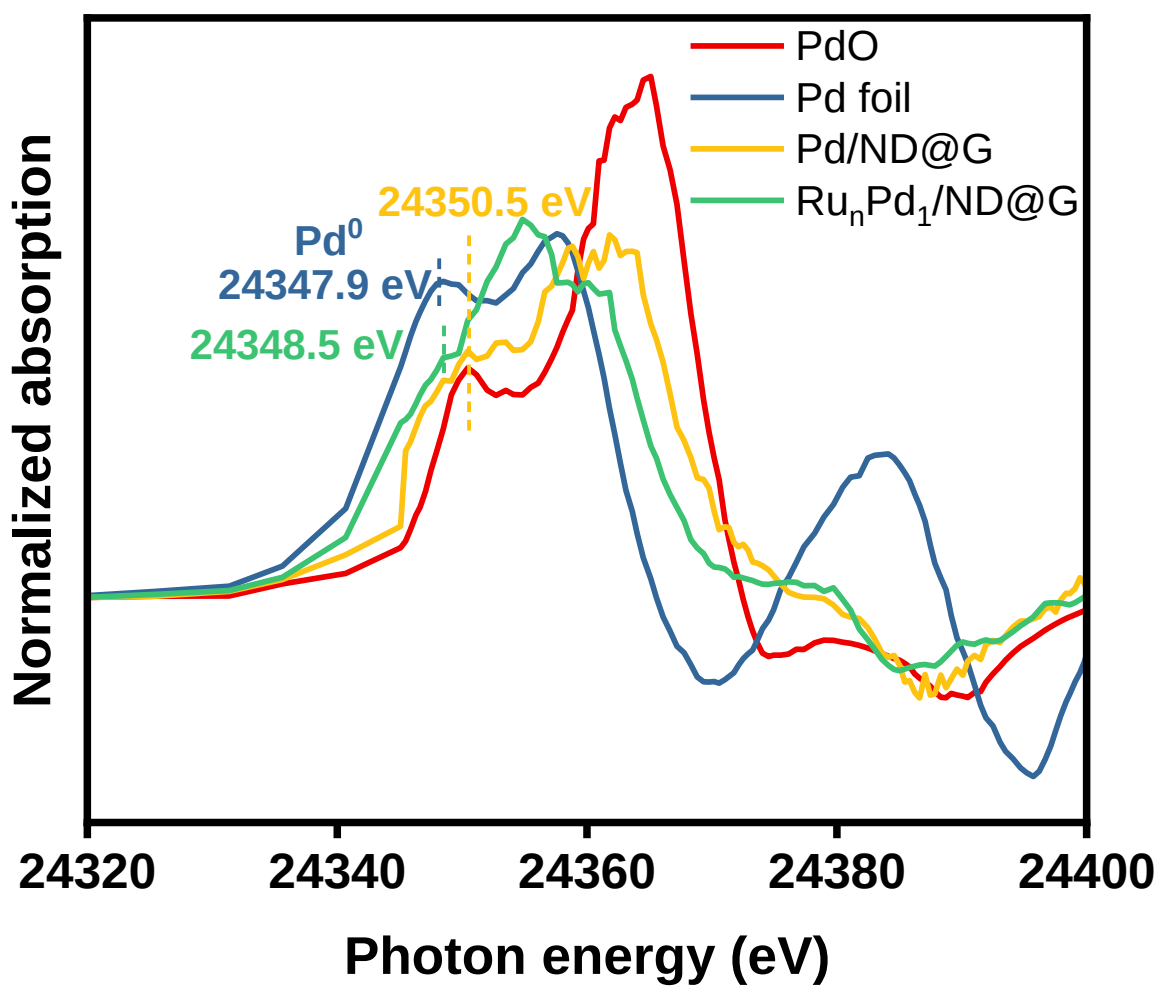

Figure R2. The first derivatives of XANES spectra of Pd/ND@G and Ru_nPd_1/ND@G.

3: Toluene hydrogenation is a structure-sensitive reaction. Have you considered the fully exposed Ru clusters with other numbers of Ru atoms as the DFT models? Such as Ru2, Ru4 and Ru2Pd1 etc.

Response: We thank the reviewer for the question. We also investigated the activities of Ru_2Pd, Ru_4. The results are shown in **Figure R3**, **R4, and R5.** The results show that Ru_2Pd, Ru_3 and Ru_4 present slower reaction kinetics toward TOL hydrogenation compared with Ru_3Pd. They suffer from weakened adsorption capability (Ru_2Pd_1/ND@G and Ru_3/ND@G) or slow hydrogenation kinetics (Ru_4/ND@G and Ru_3/ND@G). In experiments, those clusters present slightly different Ru-Pd and Ru-Ru coordination and may also exits in the mixtures of the catalytic systems Nevertheless, the proposed Ru_3Pd_1/ND@G catalyst not only exhibits the best consistency with experimental characterizations but also demonstrates optimal catalytic activities among the mixture of small-sized, fully exposed Ru-Pd clusters.

Please note that for Ru_3Pd_1/ND@G, Ru_3/ND@G, in response to the third reviewer's question regarding the verification of structural vibrational frequencies, we identified some structures with soft rotational degrees of freedom, which led to additional imaginary frequencies. Therefore, we re-optimized these structures. These optimizations resulted in very small changes to the structures and energies, and we have reflected these changes in the figures. These changes do not affect any of the previously proposed conclusions.

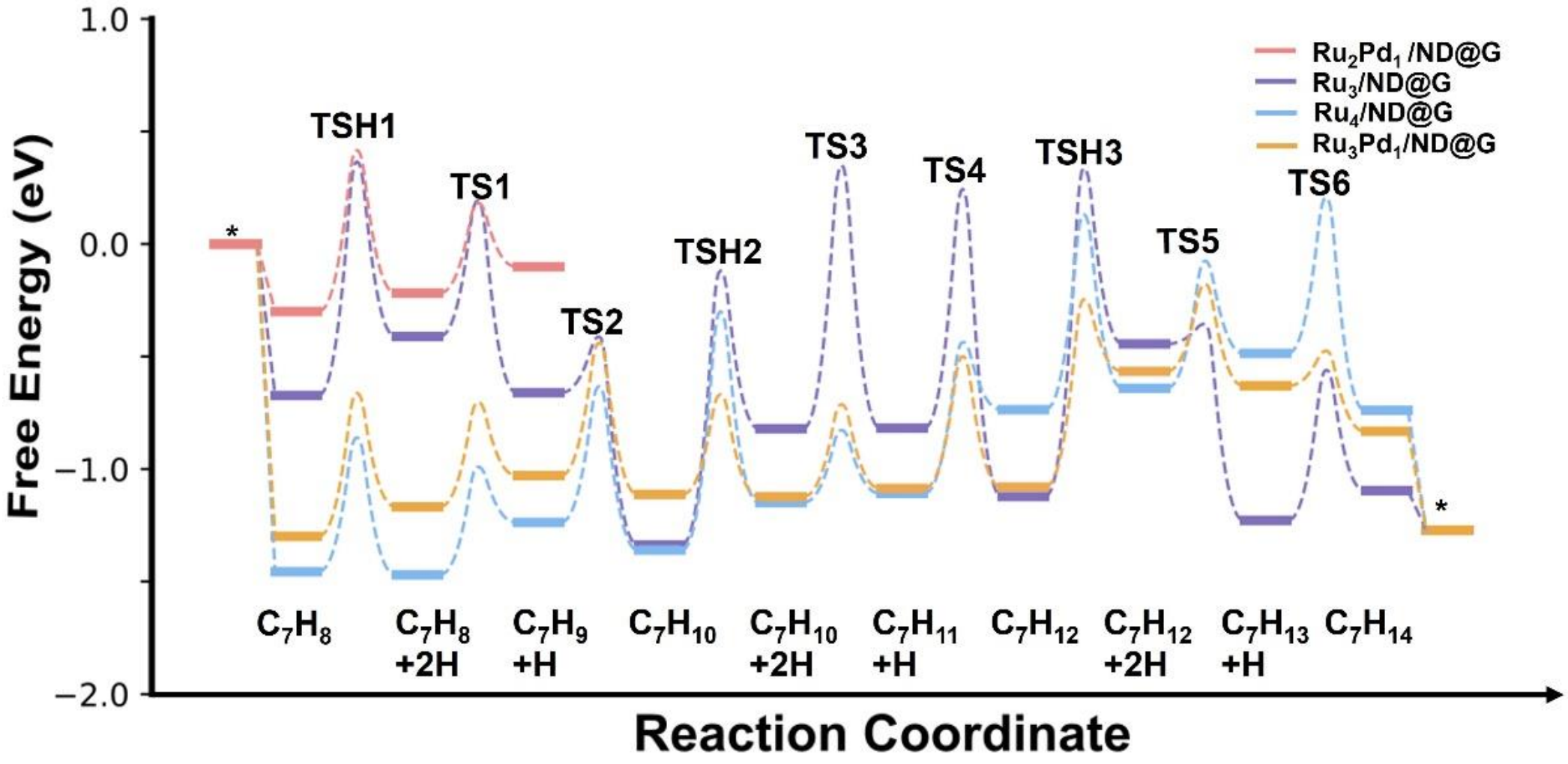

Figure R3. Free energy profiles for TOL hydrogenation to MCH on Ru_2Pd_1/ND@G, Ru_3Pd_1/ND@G, Ru_3/ND@G, Ru_4/ND@G.

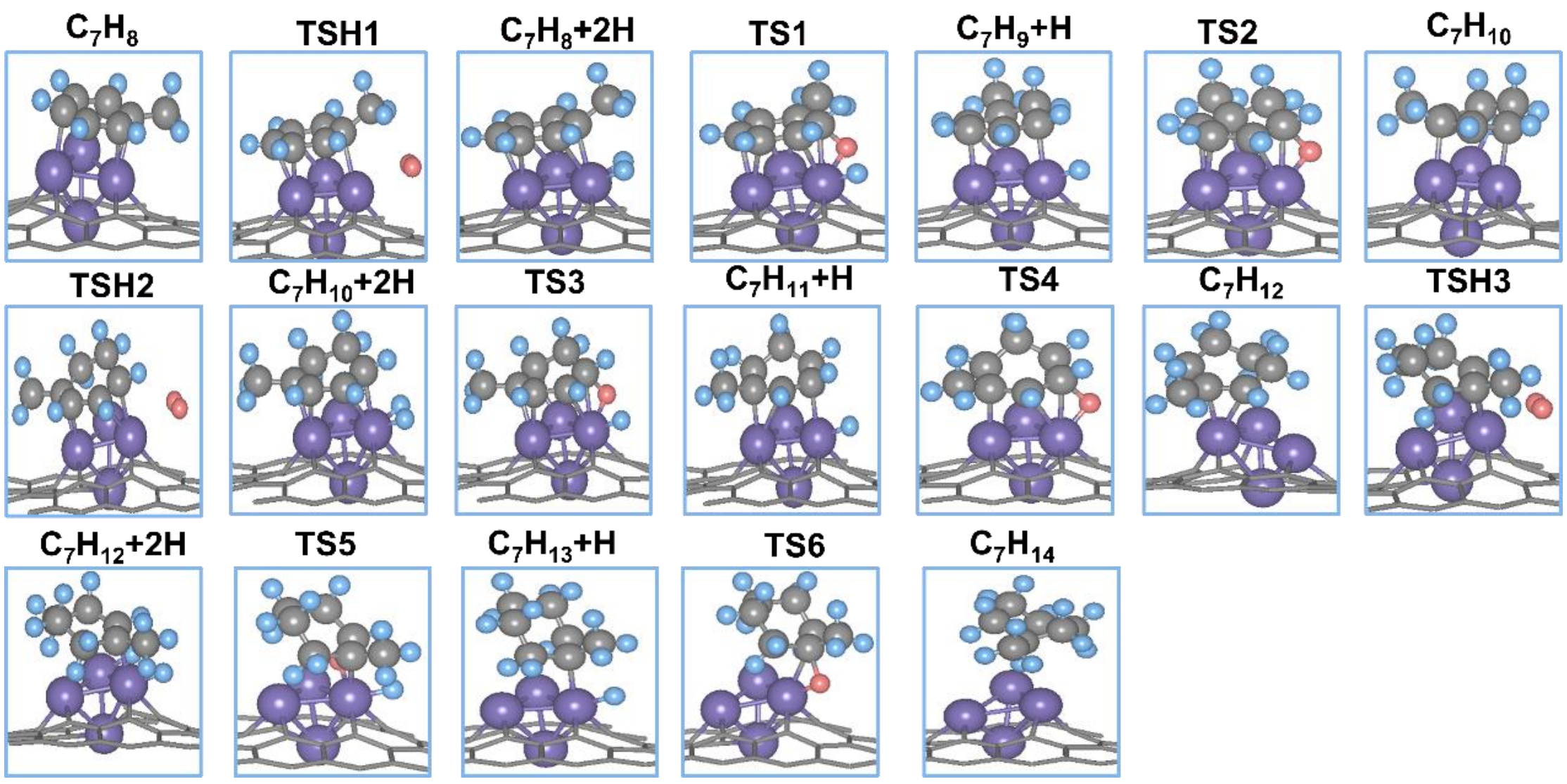

Figure R4. Structure of TOL hydrogenation to MCH on Ru_4/ND@G, Carbon atoms in reactant are shown with gray balls, and carbon atoms on graphene are only indicated by lines for simplicity. activating H atoms in red, other H atoms in blue. Ru atoms are shown with purple spheres, and Pd atoms are shown with yellow spheres.

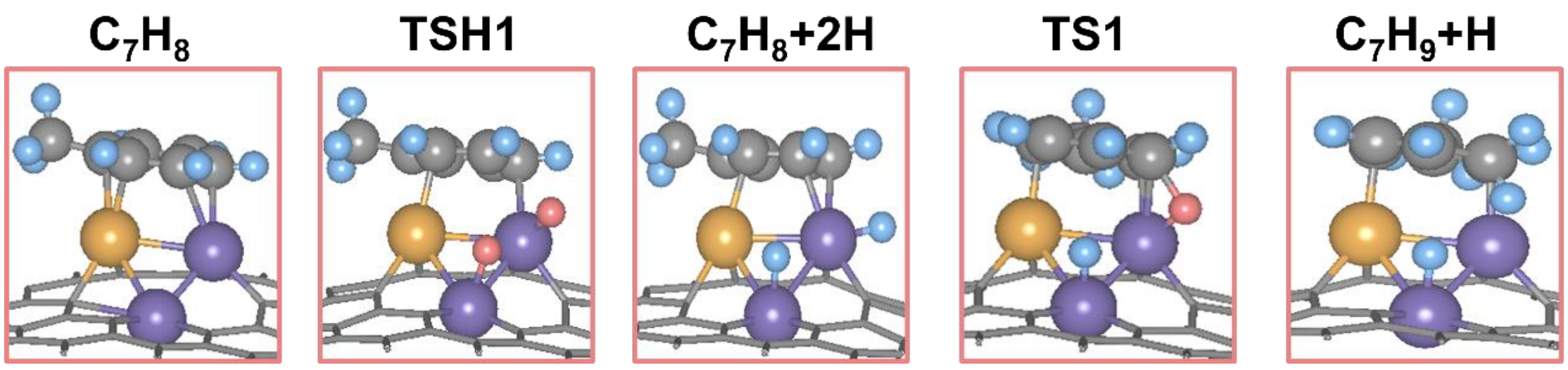

Figure R5. Structure of TOL hydrogenation to MCH on Ru_2Pd_1/ND@G, Carbon atoms in reactant are shown with gray balls, and carbon atoms on graphene are only indicated by lines for simplicity. activating H atoms in red, other H atoms in blue. Ru atoms are shown with purple spheres, and Pd atoms are shown with yellow spheres.

4: In the manuscript, the author used RunPd1/ND@G with a Ru loading of 0.3 wt% and a Pd loading of 0.05 wt%. Could other loading ratios lead to even better catalytic performance? A brief optimization study or discussion would be helpful.

Response: We thank the reviewer for this valuable suggestion. We explored different metal loading ratios while keeping the total molar amount of noble metals constant across all experiments., including 0.5 wt% Ru + 0.5 wt% Pd, 0.5 wt% Ru + 0.1 wt% Pd, 0.5 wt% Ru + 0.05 wt% Pd and 0.1 wt% Ru + 0.05 wt% Pd. As shown in **Figure R6**, under identical reaction conditions, only the Ru_nPd_1/ND@G catalyst with 0.3 wt% Ru and 0.05 wt% Pd exhibited both excellent activity and lower total metal loading, making it more cost-effective.

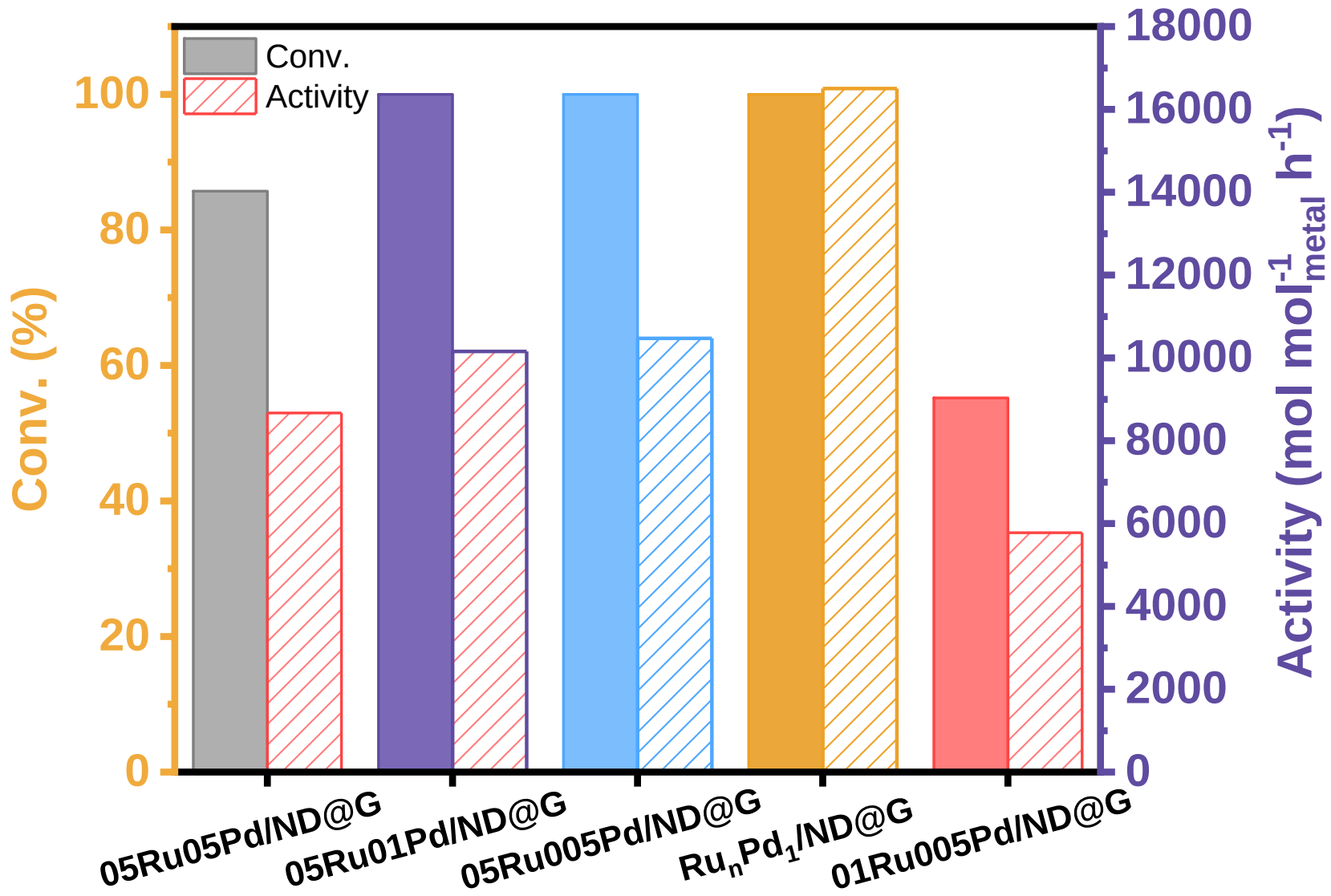

Figure R6. The activity and TOL conversion (1 h) of different metal loading ratios catalysts.

Reviewer #2 (Remarks to the Author):

This is a highly original paper by a top team of researchers demonstrating the benefits of RunPd1 fully-exposed clusters synergy for the hydrogenation of toluene. The wisely fabricated RunPd1/ND@G catalysts and revealed that the introduction of Pd optimize toluene adsorption,

facilitate TOL activation and enhances MCH desorption, resulting in the robust performance in the solvent-free hydrogenation process. It is very nice to see that the Pd single atom and fully exposed Ru clusters preserve an outstanding toluene hydrogenation activity under the solvent-free conditions, which has been rationalized with experimental and theoretical calculations. I strongly recommend acceptance of this paper in Nature Communications and request the following revision:

We appreciate the reviewer for the valuable suggestions. The following are point-to-point responses based on the reviewer's comments and suggestions.

Comments:

1: The authors should provide more scientific justification for selecting ND@G as the support.

Response: We thank the reviewer for this valuable suggestion. The nanodiamond/graphene hybrid carbon material (ND@G) is an intriguing support that our team has studied for many years. We can systematically control the microstructure and surface chemistry of nanodiamond (ND) through annealing treatments in an inert atmosphere. After a thermal treatment at 1100°C, the surface of ND can be reconstructed into an ultrathin, curved, and defect-rich sp^2 graphene nano-shell reinforced by a sp^3 diamond nano-core (**Figure R7**) that could anchor isolated metal atoms through Metal–C bond to obtain atomically dispersed catalysts. The role of nanodiamond is that it is a perfect substrate for the grown of highly defective few-layer graphene outer sheet with curvature, which is pivotal to preparation of the catalysts. Therefore, for this sp^3/sp^2 structure with pronounced curvature， we denoted it as ND@G. And the relevant details of this interesting material could be followed in our previous publications. (J. Am. Chem. Soc. 2018, 140, 13142-13146, Nat. Commun., 2023, 14, 2588, Nat. Commun., 2024, 15, 4887, Nat. Cat., 2025, 8, 436-447.)

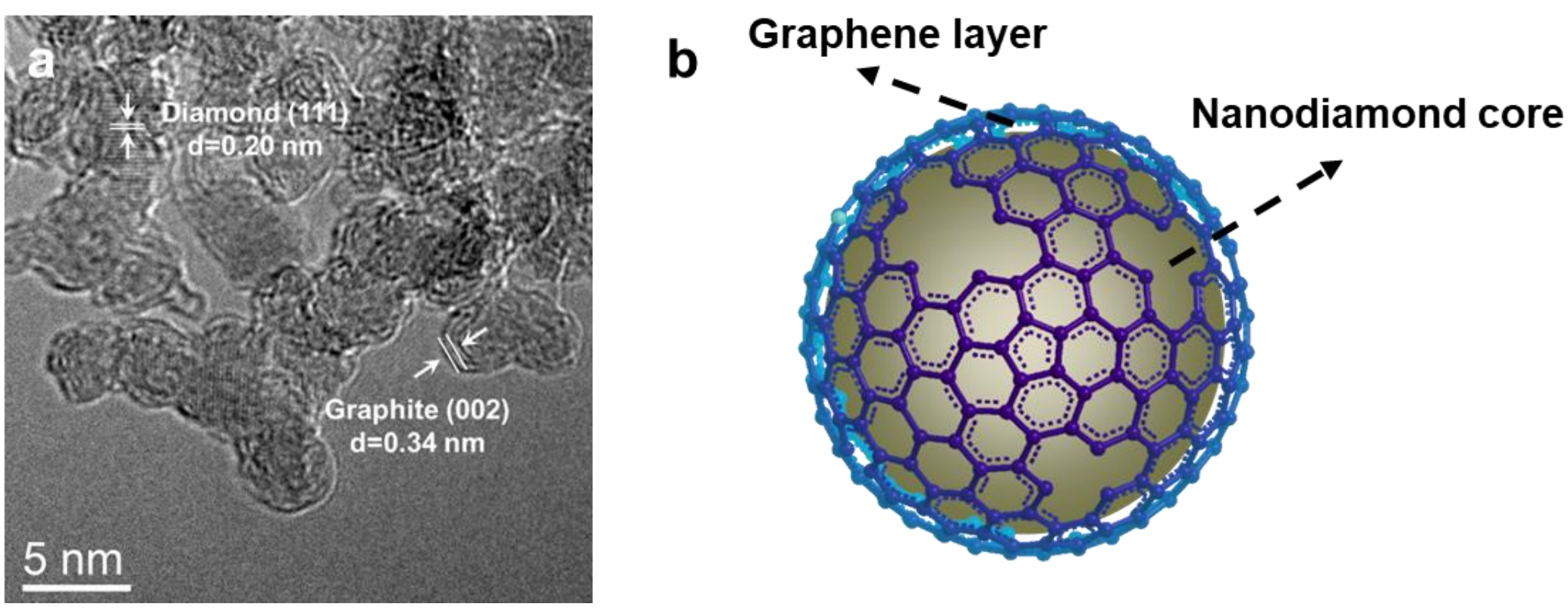

Figure R7. (a) HRTEM image of the as-prepared ND@G support with nanodiamond core and defective graphene shell, (b) Structure diagram of ND@G.

2: In line 239, the author mentioned "after 5 cycles the RunPd1/ND@G catalyst also exhibit

stability without significantly activity degradation (Figure 3e)". The structure of the used catalyst should also be confirmed to be stable.

Response: We appreciate the reviewer's suggestion. To verify the structural stability of the catalyst after cycling, we conducted HAADF-STEM image of the Ru_nPd_1/ND@G catalyst after 5 cycles. As shown in **Figure R8**, the morphology and nanostructure of the used catalyst remain nearly identical to those of the fresh catalyst, confirming that the catalyst is structurally stable under the tested conditions. We added in manuscript "HAADF-STEM images revealed that the fully exposed bimetallic Pd-Ru clusters in the recycled catalyst remained highly dispersed on the ND@G surface without agglomeration (Figure S13), indicating the excellent structural stability of Ru_nPd_1/ND@G." (Please see Lines 248-251)

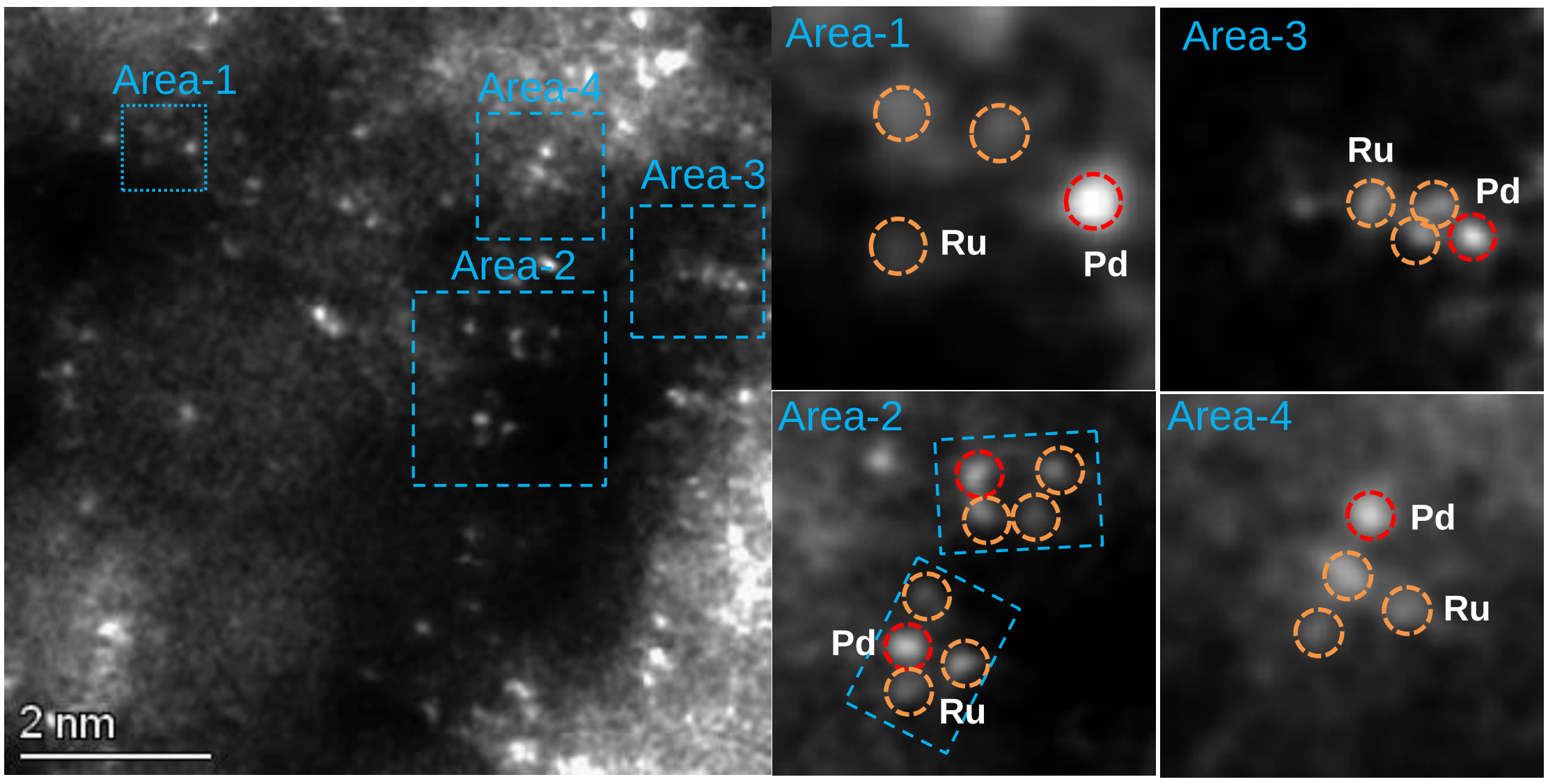

Figure R8. The high resolution HAADF-STEM image of the Ru_nPd_1/ND@G after 5 cycles.

3: The performances of common reference catalysts (e.g., commercial Ru/C, Pd/C) under identical reaction conditions are not included. Such a comparison would help readers better assess the practical advancement represented by RunPd1/ND@G.

Response: We thank the reviewer for this valuable suggestion. We have conducted comparative experiments using commercial Ru/C and Pd/C under identical reaction conditions as Ru_nPd_1/ND@G. Importantly, the molar number of noble metals (Ru or Pd) was kept the same for all catalysts to ensure a fair comparison. As shown in **Figure R9**, both commercial Ru/C and Pd/C exhibited much lower activity compared to Ru_nPd_1/ND@G.

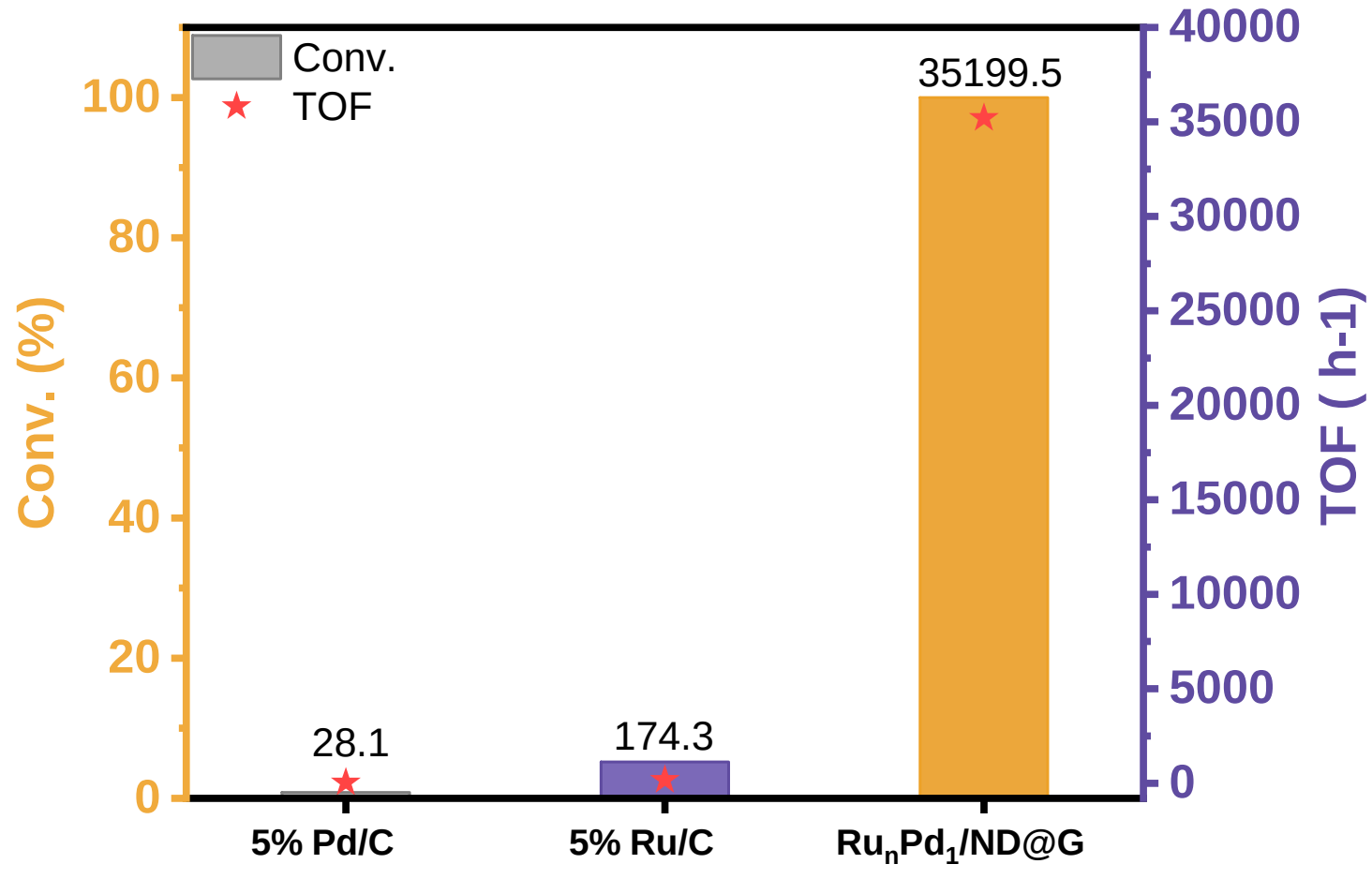

Figure R9. TOF values and TOL conversion (1 h) for the commercial Pd/C, Ru/C and Ru_nPd_1/ND@G with the same molar number of noble metal under solvent-free conditions. (Reaction conditions: H_2 pressure, 1.5 MPa; temperature, 100 °C; reaction time, 1 h.)

4: In the manuscript, line 293 "The results indicated that the Ru3Pd1/ND@G catalyst deliver better reactant adsorption and the product desorption kinetics" toluene adsorption was not experimentally verified.

Response: We thank the reviewer for the nice suggestion. Following the reviewer's suggestion, we add the toluene adsorption by TPD (**Figure R10**). The TOL adsorption temperature on Ru_nPd_1/ND@G catalyst was higher than those on Ru/ND@G catalyst and Pd/ND@G, which enhance the TOL adsorption. We add and rewrite "Furthermore, TOL-TPD were performed to probe the adsorption ability of toluene on the three catalyst surfaces. The desorption temperature of toluene was 333 °C on Ru_nPd_1/ND@G and 303 °C on Ru/ND@G (Figure S15), indicating the enhanced adsorption of toluene on Ru-Pd sites, which is consistent with the DFT result (Figure S18). The weakened desorption of MCH and the enhanced adsorption of TOL synergistically contribute to the high reactivity of the Ru_nPd_1/ND@G catalyst." (Please see Lines 274-279)

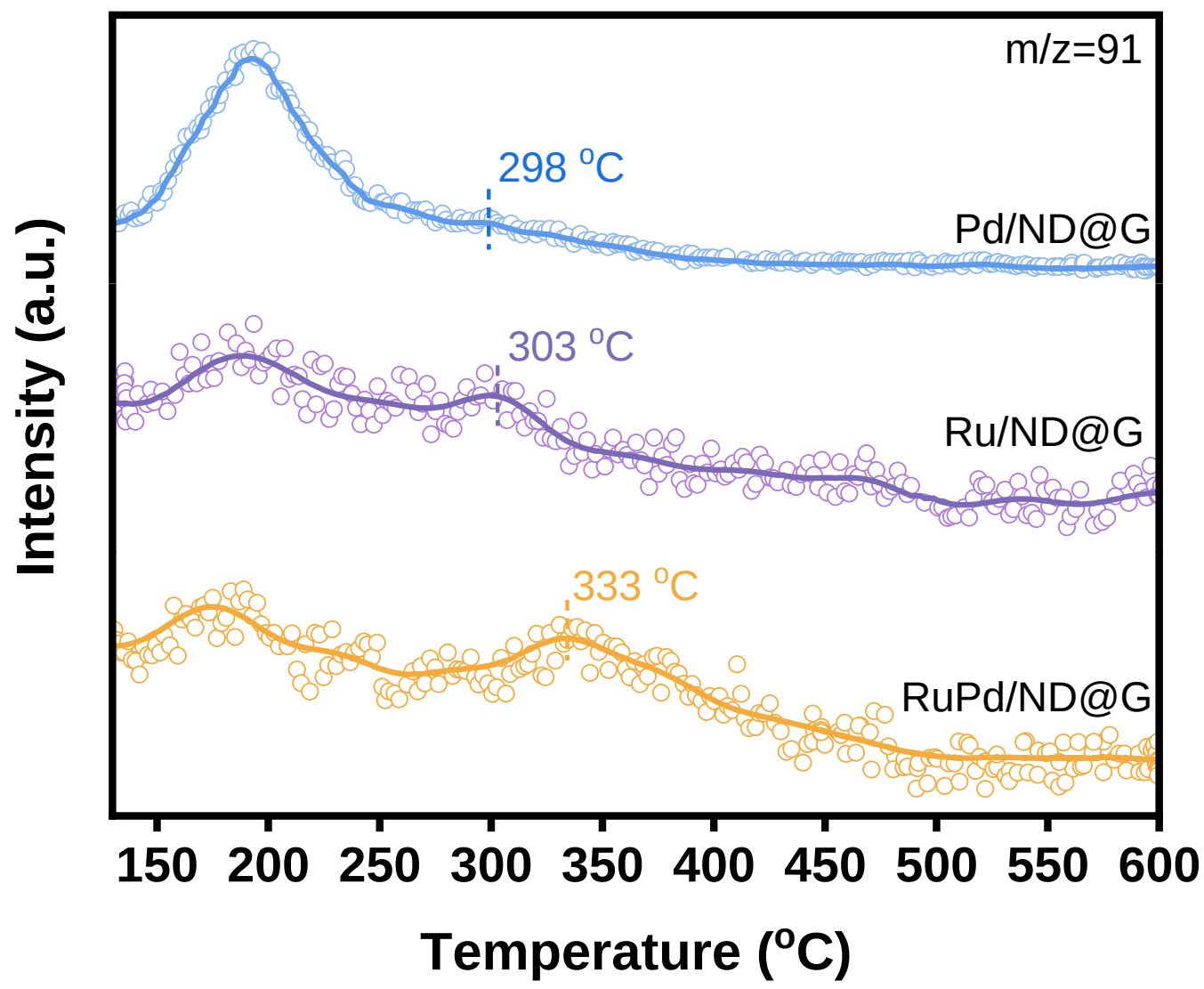

Figure R10. TOL-TPD profiles of the different catalysts.

5: In the manuscript, line 91 “to efficiently of TOL hydrogenation to MCH using H2” should be corrected to “to achieve efficient TOL hydrogenation to MCH using H2”.

Response: We wholeheartedly thank the reviewers for spotting the mistakes in our original submission. We corrected the manuscript “Therefore, there is strong incentive to construct highly active catalyst systems that do not require the use of solvent for the efficient hydrogenation of TOL to MCH using H_2.” (Please see Line 92-94)

Reviewer #3 (Remarks to the Author):

The authors report a catalyst for the hydrogenation of toluene with mixed Ru and Rd metals on a graphene/nanodiamond support material. The catalyst exhibits impressive turnover frequencies and stability under optimized conditions. The catalyst is well characterized and the proposed structure consisting of small ensembles of Ru and Pd atoms is interesting. I expect the understanding gleaned from this study regarding the catalytic properties of these small ensembles, which are larger than single-atom centers yet smaller than nanoclusters in typical supported metal catalysts, will be valuable to the community.

Nevertheless, there are instances where the evidence provided do not fully support the claims and conclusions. There are also aspects of the analysis and interpretations that are not fully articulated, challenging the reader to follow the flow of logic. I recommend that the authors address these aspects by revising the article according to the major and minor comments below, before the article is reconsidered for publication.

We sincerely thank the reviewer for the thorough evaluation of our work. We deeply value your insights and have addressed each concern as follows.

Major comments:

1. Some terms are used without a clear definition, making it challenging to follow the claims made in the article. These include:

a. "…fully exposed cluster catalysts (FECCs) with atomic dispersion and ensemble sites" (line 62) and "fully-exposed Ru cluster" (line 147). I recommend clarifying precisely what characterizes a "fully exposed cluster". For instance, is it a cluster with a specific range of atoms in which all atoms are coordinately unsaturated (i.e., a nanocluster with all metal atoms on the surface)?

Response: We thank the reviewer for this careful comment. Fully exposed cluster catalysts (FECCs) feature an ultrasmall size below 1 nm, which eliminates bulk atoms and reduces the average coordination number of metal atoms, as well as a small contact angle with the support that forms a layered structure to enhance metal-support interaction and cluster stability. We added the sentence in the manuscript "fully exposed cluster catalysts (FECCs) emerge as a type of catalyst have attracted growing attention as highly active species for various catalytic reactions. The catalysts feature an ultrasmall size below 1 nm, which eliminate bulk atoms and reduce the average coordination number of metal atoms, as well as a small contact angle with the support that form a layered structure to enhance metal-support interaction and cluster stability." (Please see Line 62-67) And the detailed description of fully exposed clusters could be followed in our review article (ACS Cent. Sci. 2021, 7, 262-273).

b. "…large-scale organic catalysis…" (line 229) and "reaction scale tolerance" (line 234). I recommend clarifying the definition of large-scale and reaction scale, and how the reaction of 100 mmol of TOL reported in Table 1 corresponds to that definition.

Response: We appreciate the reviewer for the very nice comment. We have now defined "large-scale" as reactions using ≥10 mmol of substrate (gram-scale), which is substantially larger than typical screening tests (0.1-1 mmol). The 100 mmol reaction in Table 1 (9.2 g of toluene) fully meets this definition. We added this clarification in the revised manuscript "To verify the practicality of Ru_nPd_1/ND@G catalyst for large-scale organic catalysis (substrate amount ≥10mmol), the reaction was scaled up by 20 times relative to the laboratory-scale conditions. As shown in Figure 3c and entries 3-5 in Table 1, the catalytic performance nearly identical to that observed at the lab scale, highlighting the excellent reaction scale tolerance of Ru_nPd_1/ND@G. Notably, catalysts used in industrial processes often suffer from loss of activity

and stability due to metal leaching or sintering, which can be exacerbated by strong coordination of substrates or products with the metal surfaces. In contrast, our catalyst maintains its performance under scaled-up conditions, underscoring its potential for practical applications." (Please see Line236-244)

2. The interpretations of Figure 1 are hard to follow. Specifically:

a. The authors write that "… RunPd1/ND@G cluster catalyst was well dispersed on the ND@G surface". Here, it is unclear what aspect of the catalyst is well dispersed. From the context, I think this refers to Ru atoms, Pd atoms, or clusters of Ru and Pd atoms. I recommend the authors clarify precisely to avoid misinterpretations.

Response: We thank the reviewer for the comment. We agree that the original sentence "…Ru_nPd_1/ND@G cluster catalyst was well dispersed on the ND@G surface" is ambiguous. To avoid misinterpretation, we have revised the manuscript "…the Pd-Ru bimetallic clusters in Ru_nPd_1/ND@G were well dispersed on the ND@G surface". (Please see Line138)

b. It is not clear what evidence the authors have to assign the blue highlighted boxes in Figure 1c to Ru-Pd clusters. I recommend the authors provide and justify the classification criteria used. For instance, is there an interatomic distance cutoff?

Response: We sincerely thank you for the insightful and constructive comments. We are sorry about that direct EELS line scanning across the individual, fully exposed clusters proved exceptionally challenging. Therefore, we employed extensive the HAADF-STEM images at different regions and magnifications to demonstrate the special distribution of Pd and Ru species, which was shown in **Figure R11**. We add the images in Supporting information and add "The extensive HAADF-STEM images at different regions and magnifications were shown in Figure S5." (Please see Line145)

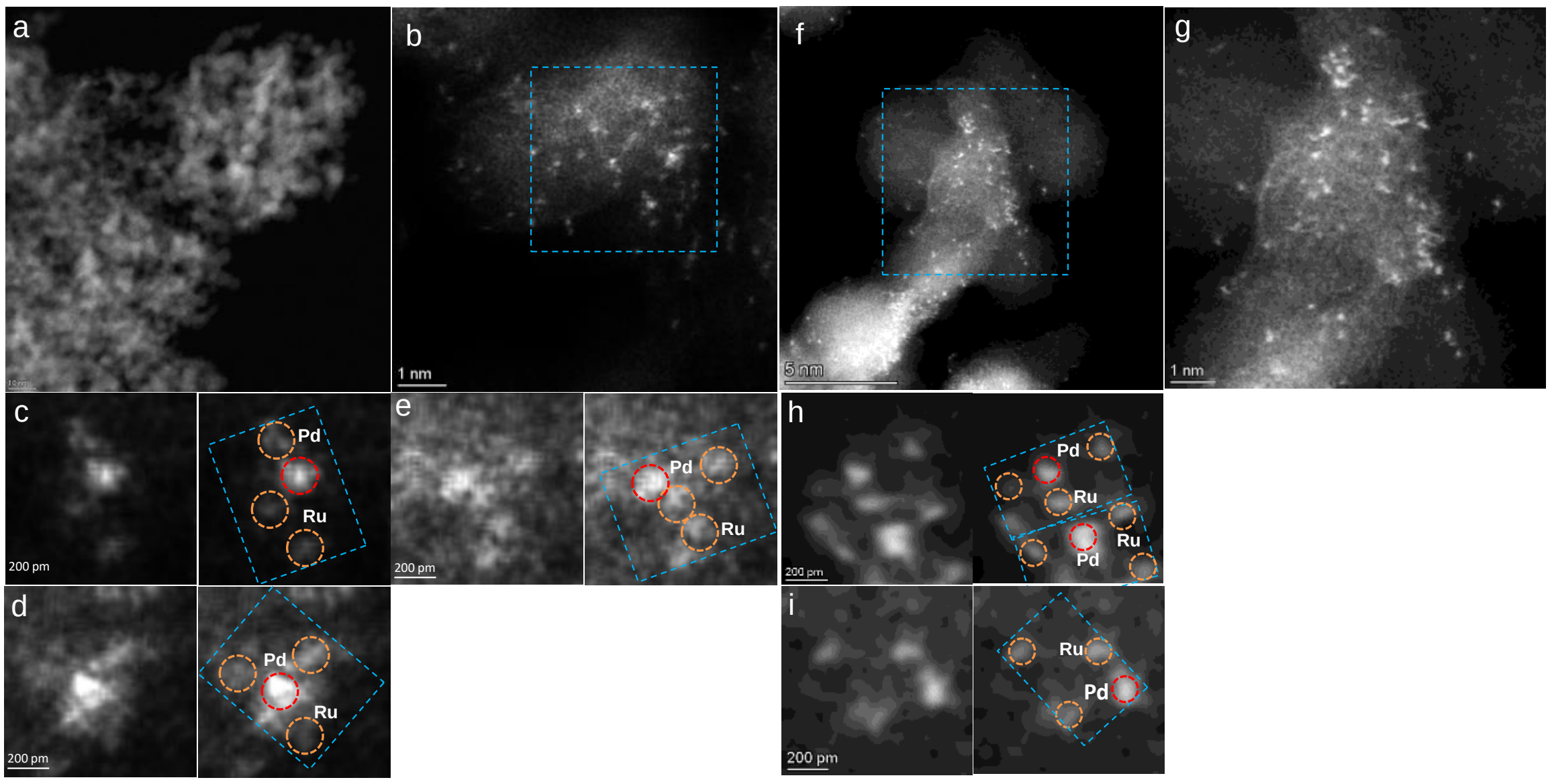

Figure R11. The HAADF-STEM image of the Ru_nPd_1/ND@G.

c. It is not clear how it is deduced from the STEM images that these atoms are "fully exposed" and not present as small nanoclusters with some constituent atoms inaccessible to reactants (here, the precise definition of "fully exposed" as mentioned above would be helpful).

Response: We thank the reviewer for the comment. We have explicitly defined a "fully exposed cluster" in the revised manuscript (Line227-236). Our conclusion is not based solely on STEM observations, but on the combination of multiple characterization results. The HAADF-STEM images showed atomically dispersed bright spots without observable nanoparticles. If there were lager clusters with buried atoms, the images would show multiple atomic layers or a thicker profile, which is not observer. Moreover, EXAFS analysis of Ru_nPd_1/ND@G revealed the coordination number of 2.4 for Pd-Ru and 1.5 for Ru-Ru. The results were significantly lower than Ru or Pd nanoparticles (typically>8), consistent with a fully exposed cluster rather than a particle containing interior atoms

3. I recommend that the conditions, conversions, and reaction times for the apparent activation energy measurement (Fig. 3b) be reported. Please clarify if any of the conversions are near 100% (as in Fig. 3a), in which case the rate may be artificially underestimated by normalizing rates by a time that is longer than needed for complete reaction based on the TOF definition in line 399.

Response: We thank the reviewer for this careful comment. For the apparent activation energy measurement shown in Figure 3b, all reactions were controlled to achieve low conversions (<20%) at each temperature. Moreover, the TOF value was calculated at a toluene conversion rate controlled between 10-15% to calculate the active site.

4. The authors report in Fig. 3a that the TOF on the RunPd1/ND@G catalyst (35199.5) is ~6-fold larger than on Ru/ND@G (5928.9). In Fig. 3f, however, the TOF for Ru/ND@G is reported to be 9580.3. Please explain this discrepancy.

Response: We appreciate the reviewer for the insightful comment. The TOF of value of 5928.9 h^{-1} in manuscript Figure 3a was obtained under solvent-free conditions with a Ru loading of 0.3 wt% (identical to that in Ru_nPd_1/ND@G). In contrast, the value of 9580.3 h^{-1} shown in Figure 3f was taken from a previous work, in which the Ru_n/ND@G with a loading of 0.5 wt% was tested in dodecane as a solvent. Therefore, these two TOF values are not directly comparable. We have revised

5. The authors report that the incorporation of Pd into Ru clusters in RunPd1/ND@G improves the performance of the Ru catalyst. While this may be the case when compared with Ru/ND@G catalyst, there are several Ru-based catalysts with similar TOFs, including Ru/rGO, Ru-OMC, etc. Please explain the high activity of these monometallic Ru-based catalysts compared with Ru/ND@G.

Response: We thank the reviewer for this comment. While some monometallic Ru catalysts supported on other materials (e.g., Ru/rGO, Ru-OMC) show high TOFs in the literatures, those values were typically obtained under different reaction conditions, such as high temperature, high pressure, the presence of solvents, fix-bed reactors or high Ru loadings. In the manuscript, Ru/ND@G was tested under solvent-free, mild conditions with a low Ru loading of only 0.3 wt%. Therefore, a direct comparison across studies is not valid. What matters is that under identical mild conditions with solvent-free, the incorporation of Pd into Ru clusters boost the TOF of Ru/ND@G by approximately 6-fold, clearly demonstrating the synergistic effect between Ru and Pd.

6. The authors report that H2-D2 exchange occurs at a lower temperature on RunPd1/ND@G than on Ru/ND@G (Fig. 3g-h). This is used as evidence that Pd promotes H2 activation facilitating hydrogenation (presumably in ensembles with Ru). Yet, Fig. S12 shows that H2-D2 exchange occurs at an even lower temperature on Pd/ND@G. This implies that the H2-D2 exchange observed on RunPd1/ND@G might be occurring on single-atom Pd sites that are not part of Ru-Pd clusters; thus, the detection of H2-D2 exchange at lower temperatures is not evidence that Pd incorporation into clusters with Ru helps to activate H2 at lower temperatures than an Ru cluster alone. With this context, please clarify the interpretation of these H2-D2 exchange data in lines 253-254, specifically how the "introduction of Pd could promoter H2

activation" for hydrogenation.

Response: We thank the reviewer for this careful analysis. The lowest H_2-D_2 exchange temperature of Pd/ND@G reflects the intrinsic H_2 dissociation ability of Pd. As shown in **Table S2**, EXAFS analysis of Ru_nPd_1/ND@G revealed the coordination number of Pd-Ru was 2.4, while no Pd-Pd or Pd-C/O coordination was detected, indicating that not existing isolated Pd single atoms. In addition, we conducted the control experiments to exclude each contribution of these two centers from Ru clusters and Pd single atoms. The catalytic of these catalysts for hydrogenation of toluene is shown in **Figure 3a**. It was found that the catalytic activity of toluene hydrogenation was negligible for Pd single atom catalysts, while Ru clusters showed low activity. The physical mixture of Ru clusters and Pd single atoms exhibited toluene hydrogenation activity comparable to that of Ru clusters alone, suggesting that monometallic Pd catalysts was not responsible for the enhanced catalytic performance. These indicated that high performance attribute to the Ru-Pd bimetallic clusters. DFT showed that the energy barrier for H_2 dissociation on Ru_nPd_1/ND@G was significantly lower that on monometallic Ru/ND@G, further supporting the synergistic interaction between Ru and Pd. We added in paper "Although Pd/ND@G exhibited the lowest H_2-D_2 exhange temperature (63°C), the Ru_nPd_1/ND@G showed no Pd- C/O or Pd-Pd bonds but contained Pd-Ru coordination. Furthermore, Pd/ND@G displayed negligible activity in toluene hydrogenation. Hence, the promotion of H_2 dissociation is attributed to the incorporation of Pd and its synergistic interaction with Ru." (Please Line 264-268)

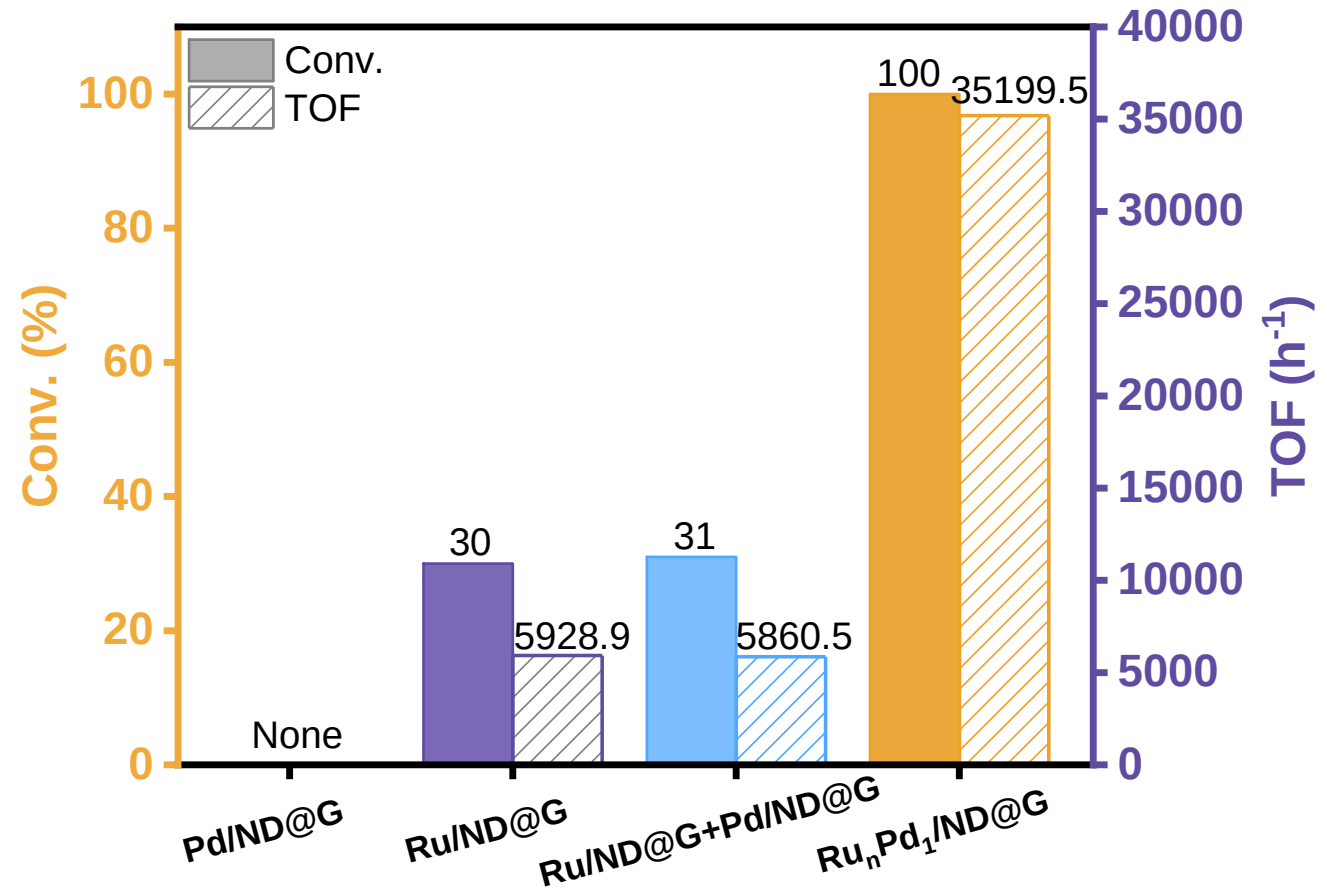

Figure 3a. TOF values and TOL conversion (1 h) of different catalysts under solvent-free conditions. (Reaction conditions: H_2 pressure, 1.5 MPa; temperature, 100 °C; reaction time, 1 h.)

Table S2. EXAFS parameters of Pd /ND@G, Ru/ND@G and Ru_nPd_1/ND@G catalysts.

Sample	Shell	Bond length (Å)	Coordination Number	σ^2 (Å²)	E0 shift (eV)	R-factor
**Pd/ND@G**	Pd-O/C	1.97±0.01	1.7	0.003±0.002	-2.6±2.1	0.020
	Pd-Pd	2.80±0.01	0.9	0.013±0.005		
**Ru/ND@G**	Ru-O/C	2.03±0.02	3.9	0.009±0.003	3.2±2.2	0.019
	Ru-Ru	2.71±0.02	2.4	0.007±0.003		
**Ru_nPd_1/ND@G**	Pd-O/C	1.94±0.01	2.7	0.014±0.001	-6.5±1.3	0.012
	Pd-Ru	2.64±0.01	2.4	0.012±0.001		
	Ru-O/C	2.08±0.02	3.2	0.007±0.001	7.8±2.5	0.018
	Ru-Ru	2.73±0.02	1.5	0.006±0.002		

[a]. The value of the amplitude reduction factor (S_0^2) lines between 0.7 and 1; [b]. Bond length is the interatomic distance; [c]. CN is the coordination number; [d]. σ^2 is Debye-Waller factor (a measure of thermal and static disorder in absorber scatter distance); [e]. E_0 shift is edge-energy shift (the difference between the zero kinetic energy value of the sample and that of the theoretical model); [f]. R factor is used to value the goodness of the fitting.

7. The Ru3 model shows a three-atom cluster with one Ru embedded in the graphene and two Ru exposed at the surface—this does not correspond to a common stable site for aromatic rings. By making this comparison between two clusters that have not only different speciation (Ru vs Ru/Pd), but also different size and shape, it makes it unclear whether adding Pd is affecting energies simply by adding another atom to expose the right site motif or by electronic differences compared to an Ru atom. To address this concern, I recommend that the authors repeat the calculates with an Ru4 cluster to isolate the electronic effects of Pd.

Response: We thank the reviewer for the insightful question. To address it, we will provide two additional results:

1. Our model shows good agreement with experimental observations in terms of coordination numbers and oxidation states.

2. Following the reviewer's suggestion, we calculated the reaction pathways on the Ru_4 cluster to demonstrate that the electronic effects of Pd are pivotal to the enhanced activity.

Regarding the rationale for selecting these specific structures:

We constructed DFT models of Ru_3/ND@G, Pd_1/ND@G and Ru_3Pd_1/ND@G to represent Ru/ND@G (**Figure 4b**), Pd/ND@G (**Figure 4b**) and Ru_nPd_1/ND@G (**Figure 4b**), respectively. The proposed catalyst structures agree well with the EXAFS determined coordination numbers as well as the representative STEM images for the catalysts.

On the other hand, the varying electronic structures of Ru_3/ND@G, Pd_1/ND@G and Ru_3Pd_1/ND@G catalysts (**Figure 4b**) also matched the while line positions from XANES and XPS. In particular, the Ru is positively charged in Ru_3Pd_1/ND@G with an average Bader charge of 0.4 |e|, being slightly larger than that in Ru_3/ND@G 0.37 |e| being in line with the slight right shifting of the white line positions of the Ru-K edge XANES (Figure 2a). Conversely, the Bader charge of Pd in Ru_3Pd_1/ND@G was 0.04 |e|, significantly lower than that of in Pd_1/ND@G 0.36 |e|. The results confirmed the enhanced Pd metallicity upon formation of Ru_3Pd_1/ND@G.

Regarding the critical role of electronic effects of Pd alloying

Following the reviewer's suggestion, along with other comments from Reviewer #1, we also investigated the catalytic activity of Ru_4 toward methylbenzene hydrogenation. The corresponding results and structures are shown in Figure R3 and Figure R4, respectively. It is evident from the Gibbs energy profiles that Ru_2Pd, Ru_3, and Ru_4 imply slower reaction kinetics for toluene (TOL) hydrogenation compared to Ru_3Pd. These systems suffer either from weakened adsorption capability (Ru_2Pd_1/ND@G and Ru_3/ND@G) or slow hydrogenation kinetics (Ru_4/ND@G and Ru_3/ND@G). Notably, while Ru_4 exhibits geometric similarities to Ru_3Pd-both possessing a three-fold hollow site resembling an FCC (111) surface, as noted by the reviewer-Ru_4 shows lower activity, reflected by its significantly higher free energy barriers (TSH3 and TS6 in Figure R3). This result confirmed the critical role of electronic effects by introducing Pd.

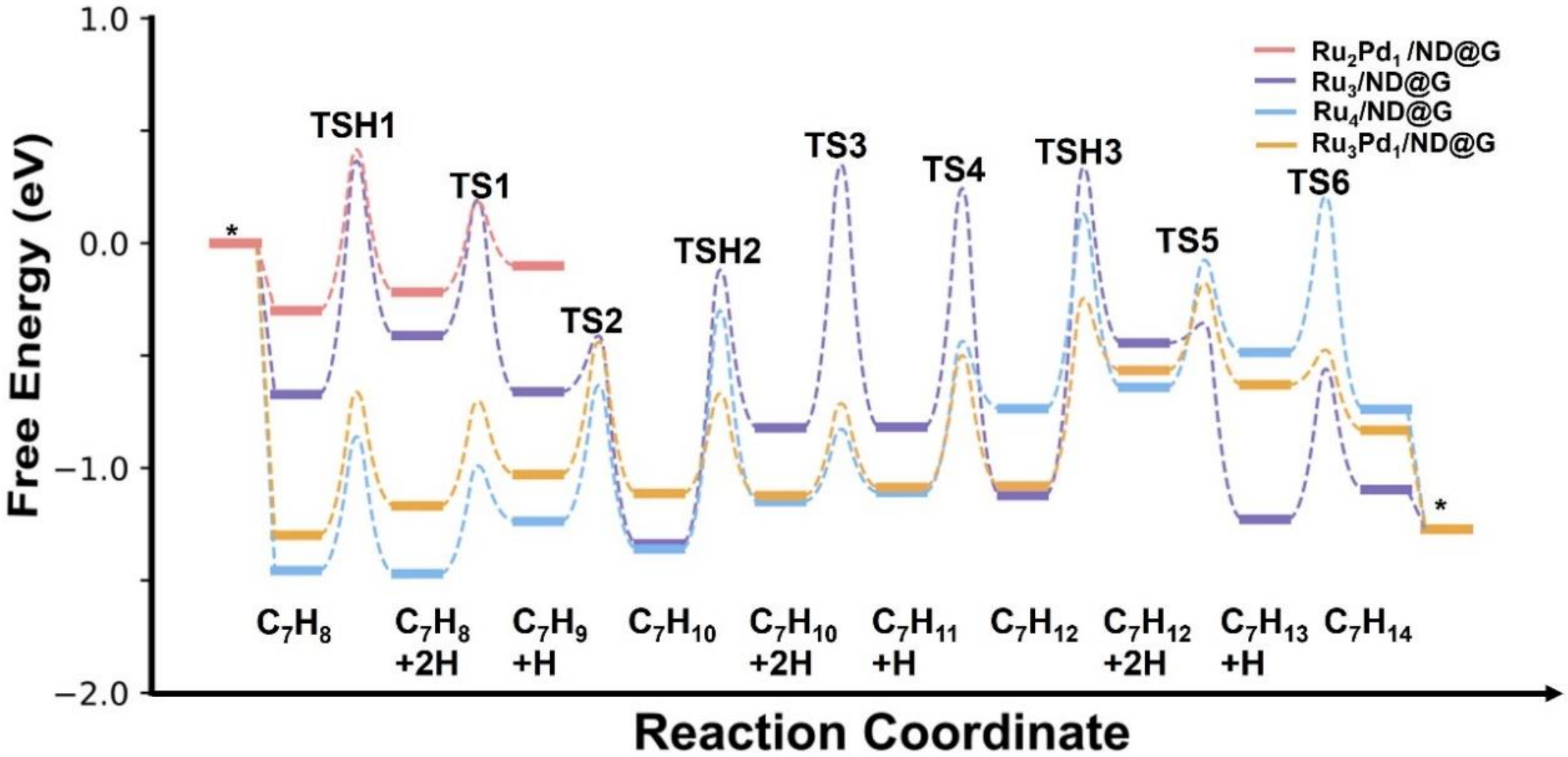

Figure R3. Free energy profiles for TOL hydrogenation to MCH on Ru_2Pd_1/ND@G, Ru_3Pd_1/ND@G, Ru_3/ND@G, Ru_4/ND@G.

Please note that for Ru_3Pd_1/ND@G, Ru_3/ND@G, in response to the reviewer's question regarding the verification of structural vibrational frequencies, we identified some structures with soft rotational degrees of freedom, which led to additional imaginary frequencies. Therefore, we re-

optimized these structures. These optimizations resulted in very small changes to the structures and energies, and we have reflected these changes in the figures. These changes do not affect any of the previously proposed conclusions.

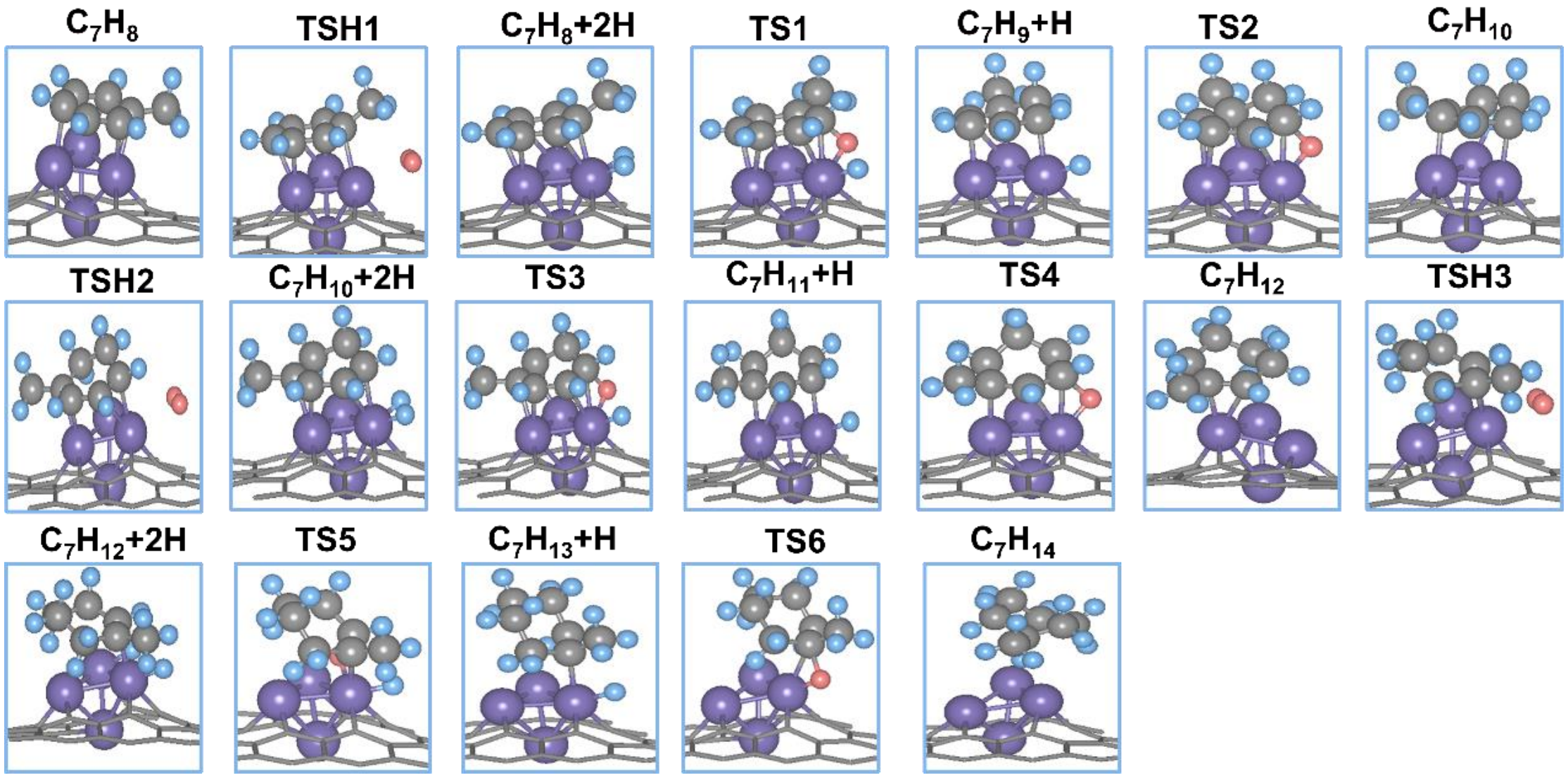

Figure R12. Structure of TOL hydrogenation to MCH on Ru_4/ND@G, Carbon atoms in reactant are shown with gray balls, and carbon atoms on graphene are only indicated by lines for simplicity. activating H atoms in red, other H atoms in blue. Ru atoms are shown with purple spheres, and Pd atoms are shown with yellow spheres.

8. The authors are missing essential computational details, including the method used to calculate vibrational frequencies, the k-point mesh used, vacuum used in z-coordinate, the DFT functional used, the supercell size for the graphene substrate, and energy convergence in self-consistency steps.

Response: We thank the reviewer for the nice suggestion. All density functional theory (DFT) calculations are carried out using the VASP (Vienna Ab initio Simulation Package)[1]. The Perdew–Burke–Ernzerhof (GGA-PBE) functional is adopted throughout this manuscript[2]. The total potential of the nucleus and core electrons is simulated by the projected augmented wave (PAW) approach, and the valence wave functions are expanded by plane waves up to a cutoff energy of 400 eV[3, 4].The Brillouin zone is sampled using a 2 × 2 × 1 k-point mesh for self-consistent calculations. To avoid artificial interactions between periodic images in the *z* direction, a 15 Å vacuum layer was applied along the *z* direction. A 5×5 supercell of graphene with vacancies was employed as the substrate for simulating the structure of ND@G support. Spin polarization calculations are always exploited. The dDsC method is used to account for the missing van der Waals interactions in PBE functional[5, 6].The self-consistent field (SCF) energy convergence threshold is set to 1.0×10^{-5} eV. Structure optimizations are conducted until the maximum residual force is less than 0.03 eV/Å or smaller. Transition states are obtained by

NEB method and then further refined with the DIMER method or CINEB method[7, 8] as implemented in ASE package.

In addition, we did not include the vibrational contribution to the Gibbs energy, assuming instead that the Gibbs energy of adsorbed species on the catalyst is approximately equal to the electronic energy of the system: $G \cong E$. This practical approach is motivated by the fact that vibrational frequencies in adsorbed and gas phases exhibit certain similarities that can partially cancel each other out. On the other hand, evaluating the vibrational entropies of adsorbed species using the commonly applied harmonic approximation can be misleading due to the presence of abundant soft modes. Only the translational and rotational entropy of gas-phase molecules is included when correcting their Gibbs energy, an approximation justified by the dominance of translational and rotational entropy in the total entropy of gas-phase species.

Please note that for Ru_3Pd_1/ND@G and Ru_3/ND@G, we calculated the frequencies of all structures. All intermediates possess only positive frequencies, while each transition state has exactly one imaginary frequency. During the frequency calculations, we identified some structures with soft rotational degrees of freedom, which led to additional imaginary frequencies. Therefore, we re-optimized these structures. These optimizations resulted in very small changes to the structures and energies, and we have reflected these changes in the figures. These changes do not affect any of the previously proposed conclusions.

Minor comments:

9. There are several sentences with unclear meaning, perhaps due to typographical error. I recommend the authors carefully proofread the article. Some include:

• Line 91: "…solvent to efficiently of TOL…"
• Line 258: "…temperature indicated facilitate MCH adsorption…"
• Line 336: "The ND power powder…"

Response: We thank the reviewer for the nice suggestion. We have carefully proofread the manuscript and revised any inadvertent errors to ensure clarity and accuracy. We corrected the manuscript "…for the efficient hydrogenation of TOL…" (Please see Line 94) "The lower desorption temperature indicates that MCH adsorption is facilitated, which contributes to the high reactivity of the Ru_nPd_1/ND@G catalyst." (Please see Line 272-274) "The ND powder was…" (Please see Line 358)

10. The "Catalytic performances" section (line 191) begins by introducing TOL hydrogenation

as a “model reaction” for the RunPd1/ND@G catalyst. Given that the title, abstract, and introduction discuss improvements to TOL hydrogenation as a main motivation for the study, calling it a “model reaction” at this stage is a bit confusing. The authors might consider a different transition sentence to avoid this confusion.

Response: We thank the reviewer for this suggestion. We revised in manuscript “The hydrogenation of TOL was used to study the catalytic performance of Ru_nPd_1/ND@G catalyst.” (Please see Line 198)

11. The EXAFS spectra were interpreted as including a Ru-O/C coordination. Since the catalyst comprises Ru, Pd, and C, it is not clear where the O is coming from. Please clarify this point.

Response: We thank the reviewer for this insightful question. In the manuscript, we used the notation “Ru-O/C” for the coordination because carbon and oxygen cannot be reliably distinguished by EXAFS due to their similar atomic numbers and backscattering amplitudes. Moreover, the surface of ND@G contained the oxygen-containing functional groups (J. Am. Chem. Soc. 2018, 140, 13142-13146). Therefore, the observed coordination could originate from either Ru-C (direct interaction with the ND@G support) or Ru-O (interaction with oxygen-containing species).

Reference:

1.Kresse G, Furthmuller J. Efficient iterative schemes for ab initio total-energy calculations using a plane-wave basis set. *PHYSICAL REVIEW B* **54**, 11169-11186 (1996).

2.Ernzerhof M, Scuseria GE. Assessment of the Perdew–Burke–Ernzerhof exchange-correlation functional. *The Journal of Chemical Physics* **110**, 5029-5036 (1999).

3.Kresse G, Joubert D. From ultrasoft pseudopotentials to the projector augmented-wave method. *Phys Rev B* **59**, 1758-1775 (1999).

4.Perdew JP, Wang Y. Accurate and simple analytic representation of the electron-gas correlation energy. *Phys Rev B* **45**, 13244-13249 (1992).

5.Steinmann SN, Corminboeuf C. A generalized-gradient approximation exchange hole model for dispersion coefficients. *The Journal of Chemical Physics* **134**, (2011).

6.Steinmann SN, Corminboeuf C. Comprehensive Benchmarking of a Density-Dependent Dispersion Correction. *Journal of Chemical Theory and Computation* **7**, 3567-3577 (2011).

7.Jonsson H, Mills G, Jacobsen KW. Nudged elastic band method for finding minimum energy paths of transitions.). WORLD SCIENTIFIC (1998).

8.Henkelman G, J nsson H. A dimer method for finding saddle points on high dimensional potential surfaces using only first derivatives. *The Journal of Chemical Physics* **111**, 7010-7022 (1999).

Reviewer #1 (Remarks to the Author):

The authors have carefully and thoroughly revised the manuscript in response to all of my previous comments and suggestions. Every point I raised has been addressed in a satisfactory manner, and the revisions have notably improved the clarity and quality of the paper. I have no further concerns and am pleased to recommend that the manuscript be accepted for publication in its current form.

Response: Thank you for your positive feedback and for recommending our manuscript for publication. We are very pleased to hear that you find it acceptable in its current version.

Reviewer #2 (Remarks to the Author):

The authors have addressed my concerns. It is now in a suitable form for publication.

Response: Thank you for your positive feedback and for recommending our manuscript for publication. We are very pleased to hear that you find it acceptable in its current version.

Reviewer #3 (Remarks to the Author):

I agree with the changes made by the authors and find that many of the comments from the previous review cycle were very well addressed. I generally find the work to be interesting and compelling. There are three follow-up points that I believe should be addressed to better support the conclusions in the study.

We sincerely thank the reviewer for the thorough evaluation of our work. We deeply value your insights and have addressed each concern as follows.

3. I appreciate the clarification on this point. I recommend that the authors specify in the methods section that the conversions were controlled to <20% for the activation energy measurement, and quantify the conversion for the TOF measurement (currently, the text reports "low conversion" without further details).

Response: We thank the reviewer for this careful comment. We added in paper "For the apparent activation energy measurement, all reactions were controlled to achieve low conversions (<20%) at each temperature." (Please see Line 416-417) "…at low conversion (<20%) of the substrate." (Please see Line 420-421)

4. The authors mentioned that "We have revised" the manuscript to explain the TOF data in Fig.

3f are not directly comparable. However, I was not able to find this revision in the highlighted text. Could the authors please specify the revision made? Furthermore, I see that Table S3 details the original studies and conditions used for the TOF reported in Fig. 3f. I recommend clarifying in lines 256-259 that (i) the TOF on the "other catalysts reported in the literatures" are reported for different conditions/solvents and (ii) that Table S3 details those conditions.

Response: We thank the reviewer for the insightful question. The TOF of value of 5928.9 h^{-1} in manuscript Figure 3a was obtained under solvent-free conditions with a Ru loading of 0.3 wt% (identical to that in Ru_nPd_1/ND@G). In contrast, the value of 9580.3 h^{-1} shown in Figure 3f was taken from a previous work, in which the Ru_n/ND@G with a loading of 0.5 wt% was tested in dodecane as a solvent. Therefore, these two TOFs are not directly comparable. We added in paper "…at the same solvent-free reaction condition (Figure 3a)." (Please see Line 219-220) We also added "The TOF of the other catalysts reported in the literature were obtained under different reaction conditions and solvents, as detailed in Table S3." (Please see Line 259-260)

5. I appreciate the point that these catalysts are reported at different conditions making direct comparisons challenging. However, I think some further discussion is warranted, especially comparing the present catalyst to the Ru/rGO catalyst. This catalyst shows a TOF of 26000 under solvent free conditions, which is comparable to the RunPd1/ND@G. The temperature is only 10°C larger, and it is not clear at these high pressures how sensitive rates are to H2 pressures. This would make the point more convincing that the alloying of Pd and Ru is crucial to the catalytic enhancement, rather than the specific size of the Ru cluster exposed on the catalyst.

Response: We thank the reviewer for this careful comment. The Ru_nPd_1/ND@G catalyst exhibited a TOF of 35199.5 h^{-1} under solvent-free conditions, 1.4-fold higher than that of Ru/rGO, despite having a much lower metal loading (0.35 wt% vs. 3.4 wt%) and operating under milder reaction conditions.
